# Supplementary material for: Sensory training system for use at home by people with complex regional pain syndrome in England: protocol for a proof-of-concept study
Source: BMJ Open. 2023 May 30;13(5):e070920. doi: 10.1136/bmjopen-2022-070920 (PMC10254991; doi:10.1136/bmjopen-2022-070920)
Supplement: Author's manuscript [file bmjopen-2022-070920.draft_revisions.pdf]

BMJ Open

BMJ Open is committed to open peer review. As part of this commitment we make the peer review history of every article we publish publicly available.

When an article is published we post the peer reviewers' comments and the authors' responses online. We also post the versions of the paper that were used during peer review. These are the versions that the peer review comments apply to.

The versions of the paper that follow are the versions that were submitted during the peer review process. They are not the versions of record or the final published versions. They should not be cited or distributed as the published version of this manuscript.

BMJ Open is an open access journal and the full, final, typeset and author-corrected version of record of the manuscript is available on our site with no access controls, subscription charges or pay-per-view fees (http://bmjopen.bmj.com).

If you have any questions on BMJ Open's open peer review process please email info.bmjopen@bmj.com

BMJ Open

A Sensory Training System (STS) for use at home by people with persistent limb pain: protocol for a proof-of-concept study

Journal:	*BMJ Open*
Manuscript ID	bmjopen-2022-070920
Article Type:	Protocol
Date Submitted by the Author:	13-Dec-2022

Complete List of Authors:	Coggins, Jessica; University of the West of England, School for Health and Social Wellbeing; Royal United Hospital Bath NHS Trust, CRPS Service<br>Grieve, Sharon; Royal United Hospital Bath NHS Trust, CRPS Service; University of the West of England, School of Health and Social Wellbeing<br>Hart, Darren; Royal United Hospital Bath NHS Trust, Medical Physics & Bioengineering Department<br>Llewellyn, Alison ; University of the West of England Bristol, School of Health and Social Wellbeing; Dorothy House Hospice<br>Palmer, Mark; University of the West of England, Department of Computer Science and Creative Technologies<br>Boichat, Charlotte; The Open University, School of Psychology and Counselling<br>McCabe, Candy; University of the West of England, School of Health and Social Wellbeing; Dorothy House Hospice

Keywords:	Electric Stimulation Therapy, PAIN MANAGEMENT, REHABILITATION MEDICINE, Clinical trials < THERAPEUTICS

SCHOLARONE™
Manuscripts

Title page

Title: A Sensory Training System (STS) for use at home by people with persistent limb pain: protocol for a proof-of-concept study

Corresponding author: Jessica Coggins, University of the West of England, Glenside Campus, Blackberry Hill, Bristol, BS16 1DD, Jessica.Coggins@uwe.ac.uk

Author's institutional affiliations:

Jessica Coggins, School of Health and Social Wellbeing, University of the West of England, Blackberry Hill, Bristol, BS16 1DD and CRPS Service, Royal United Hospitals Bath NHS Foundation Trust, Combe Park, Bath, BA1 3NG

Dr Sharon Grieve, CRPS Service, Royal United Hospitals Bath NHS Foundation Trust, Combe Park, Bath, BA1 3NG and School of Health and Social Wellbeing, University of the West of England, Blackberry Hill, Bristol, BS16 1DD

Dr Darren Hart, Medical Physics & Bioengineering Department, Royal United Hospitals Bath NHS Foundation Trust, Combe Park, Bath, BA1 3NG

Dr Alison Llewellyn, School of Health and Social Wellbeing, University of the West of England, Blackberry Hill, Bristol, BS16 1DD and Dorothy House Hospice Care, Winsley, Bradford on Avon, BA15 2LE

Dr Mark Palmer, Department of Computer Science and Creative Technologies, University of the West of England, Coldharbour Lane, Bristol, BS16 1QY

Dr Charlotte Boichat, School of Psychology and Counselling, The Open University, Walton Hall, Milton Keynes, MK7 6AA

Professor Candida McCabe, School of Health and Social Wellbeing, University of the West of England, Blackberry Hill, Bristol, BS16 1DD and Dorothy House Hospice Care, Winsley, Bradford on Avon, BA15 2LE

Word count (excluding title page, abstract, references, acknowledgements, contributions, figures, and tables): 3770

Keywords: Complex Regional Pain Syndrome, Sensory Training System, Home use, Sensory discrimination, Electrical stimulation, Game-based therapy.

For peer review only - http://bmjopen.bmj.com/site/about/guidelines.xhtml

ABSTRACT

Introduction: Complex Regional Pain Syndrome (CRPS) is a disabling and distressing chronic pain condition characterised by a range of sensory, motor, autonomic and trophic symptoms. UK guidelines recommend therapy interventions to help normalise touch perception through self-administered tactile and thermal desensitisation activities. Interventions have been developed, aiming to help individuals broaden their sensory experience, therefore relieving chronic pain. However, therapy-led interventions often experience practical constraints and poor adherence. In response, a sensory training system (STS) device has been designed for unsupervised independent use at home.

Methods: This proof-of-concept study aims to explore whether people with CRPS will use the device in their own homes for 30 minutes a day for 30-days. The study also aims to determine whether the STS device will change tactile acuity and perceived levels of pain intensity, pain interference, sensitivity, or feelings towards the affected limb.

The study will seek to recruit 20 eligible participants. Participants will be asked to measure tactile acuity and complete an online questionnaire before and after use of the device. Participants will also be asked to complete a daily diary. Upon completion of the 30-day use, participants will be invited to take part in a semi-structured interview to explore their experiences of using the device.

Analysis: The questionnaire and device-use data will be analysed using descriptive statistics. PROMIS interference items will be scored using the online Assessment Center Scoring Service or using the look-up table in the PROMIS scoring manual. The qualitative data will be thematically analysed.

Ethics and dissemination: This study has been given a favourable opinion by London-Stanmore Research Ethics Committee (ref 21/LO/0200) on 19th April 2021 and approved by the NHS Health Research Authority, UK on 7th June 2021. Dissemination will include peer reviewed publications, presentations at conferences, social media, and reports to the funder and patient charities.

Trial registration: The trial was registered with ISRCTN registry on 28th May 2021 (ISRCTN89099843).

For peer review only

Article Summary

Strengths and limitations of this study

- The study team compromises a multidisciplinary team with varying backgrounds.
- Due to the pandemic the study was redesigned to be delivered remotely, broadening the geographic location of participants.
- Public contributors have been involved in all aspects of the study including design and monitoring.
- Medicines and Healthcare products Regulatory Agency approval gained for the clinical investigation of this non-CE marked STS device.
- The remote aspect of the study limits personal contact between researchers and participants, it also excludes individuals with limited or no access to the online technology required to participate, and excludes those who are unable to identify a second individual to complete the two-point discrimination assessment.

review only

For peer review only - http://bmjopen.bmj.com/site/about/guidelines.xhtml

INTRODUCTION

Background and rationale

Chronic pain affects around half of the adult population; of these, 5.5 million people experience neuropathic pain such as amputee phantom limb pain (PLP), post-stroke pain and Complex Regional Pain Syndrome (CRPS) [1]. Such conditions can be particularly challenging to treat. No more than half of patients treated with pharmacotherapy, experience clinically meaningful pain relief, and very few, if any, experience complete pain relief [2]. In addition, discontinuation of treatments is common due to unwanted adverse effects [2]. These populations experience high levels of disability, and care is primarily focused on reducing pain, preserving, or restoring function, and enabling patients to self-manage their condition [3].

The annual economic consequences of chronic pain are substantial for society and the individual, and higher than most other health conditions [4]. Data published in 2002, demonstrated General Practitioner (GP) attendances in the United Kingdom (UK) for chronic pain equated to 4.6 million appointments per annum, costing approximately £69 million for GP appointments alone [5]. In 2007, approximately £584 million was spent in the UK on prescription pain medications [6]. These figures are only likely to have increased in the intervening years with an ageing population. Therefore, there is an urgent need to find more effective therapies to alleviate the personal and societal burden of chronic pain.

CRPS is a chronic pain condition that usually develops after trauma. The condition is characterised by pain, changes in limb temperature, hair and nail growth, and sweating abnormalities [7]. One striking feature of CRPS is allodynia: pain due to a normally innocuous stimuli, that is experienced in the affected limb. This can mean, for example, clothing cannot be tolerated over the painful region. People with CRPS therefore often carefully protect their limb to prevent any physical contact to the allodynic site [8], and they can develop a strong dislike of the CRPS affected limb [9].

CRPS is associated with a decreased ability to determine the texture, temperature, and location of a stimulus to the painful area [10]. Clinically, the ability to discriminate sensations is assessed using a two-point discrimination test. Two probes are applied at the same time to the person’s skin, and they are asked to distinguish one or two contact points. The shorter the recorded two-probe distance, the better the sensory discrimination ability. Clinical studies have demonstrated that poor two-point discrimination is correlated with higher intensity of pain experienced and with altered brain representation of the affected body part [11, 12]. Interventions have

For peer review only - http://bmjopen.bmj.com/site/about/guidelines.xhtml

been developed which aim to improve two-point discrimination ability, and therefore reduce pain, by normalising the brain's representation of the painful area. Effectively these interventions seek to help the individual to broaden their sensory experience, thereby 're-finding' normal sensations and relieving chronic pain [13].

Sensory discrimination training which involves cognitive effort can improve two-point discrimination, therefore, reducing patient-reported pain. This has been evidenced for electrical stimulation in amputee PLP, mechanical stimuli, and touch tasks in CRPS, and touch stimuli in chronic low back pain [13-17]. It has been suggested that sensory discrimination training requiring cognitive effort can also have a positive effect on normalising the brain's representation of the painful area [14]. UK Guidelines for adults with CRPS recommend therapy interventions to help normalise touch perception via self-administered tactile and thermal desensitisation activities [3]. Current sensory discrimination training involves repetitive, manual application of different textiles or stimuli to the 'affected', and an 'unaffected' body site, normally by a therapist or carer [18, 19]. However, therapy is slow to deliver improvements, and adherence is poor with limited evidence for any therapist-led interventions that deliver sustained clinically important differences in the long term [20].

Mindful of the practical constraints of the NHS and poor adherence to sensory discrimination training in practice, a home-based sensory discrimination training intervention has been developed. It is essential that any device created for this purpose is well-developed, reliable, and suitable for unsupervised independent use to gain the expected benefit. Recent searches have not identified a commercially available device that encourages independent use to improve sensory discrimination within the home or hospital environment. There are, however, devices that passively stimulate areas of the body but do not include a training element in combination with an electrical stimulus. The training element is suggested to be an essential factor in improving two-point discrimination and pain [15, 21].

This current research project builds on 14 years of research and development, throughout which the authors have worked with people with CRPS to design a prototype sensory training system (STS) for use at home to improve two-point discrimination [22-24]. The overall goal is to develop a commercially available, clinically effective STS for independent home use by people who have a persistent limb pain, so they can re-gain normal limb sensation. This protocol describes the clinical testing of the next generation STS, that comprises a customised wearable electrode array and game-based training environment, which provides an easy to use and engaging electrical STS for use in the home. The device has received Medicines & Healthcare products Regulatory Agency (MHRA; ref: CI/2021/0019/GB) approval.

For peer review only - http://bmjopen.bmj.com/site/about/guidelines.xhtml

Research aim and objectives

This study aims to explore whether people with CRPS will use the STS device in their own homes adhering to the treatment plan of 30 minutes a day for 30 days (the therapeutic dose). Electrical stimulation of 30 minutes per day for a four-week period has been shown in a PLP population to deliver measurable improvements in pain intensity and changes on cortical body maps towards normalisation [14].

The objectives are:

1. To determine whether use of the STS device at the therapeutic dose changes tactile acuity (measured using two-point discrimination).

2. To determine whether use of the STS device at the therapeutic dose affects perceived levels of pain intensity, pain interference, sensitivity, or feelings towards the affected limb.

3. To establish whether participants have found using the STS device to be acceptable and feasible in terms of setting up the device, attaching and detaching the electrode array by themselves.

4. To establish whether participants have found using the STS device to be sufficiently engaging to encourage adherence to the therapeutic dose.

METHODOLOGY

This protocol is version 5.0 dated 24th February 2022.

Study design

This is a proof-of-concept study designed to explore the acceptability and useability of a customised STS.

Study setting

In response to the COVID-19 pandemic, this study will be conducted remotely across the United Kingdom. This is a single centre study based at the Royal United Hospitals Bath NHS Foundation Trust (RUH), in collaboration with the University of the West of England (UWE).

Participants and sample size

The study aims to recruit 20 adult participants who meet the inclusion criteria and who are willing to use the STS in their homes for 30 minutes per day for 30 days.

For peer review only - http://bmjopen.bmj.com/site/about/guidelines.xhtml

However, due to the severity of symptoms experienced with CRPS and the common occurrence of ‘flares’ of symptoms, we anticipate a number of withdrawals. All data will be reported, including incomplete data sets and reasons for withdrawal, if provided.

Adults meeting the Budapest clinical criteria [7] for CRPS in the upper or lower limb will be recruited from the CRPS UK Registry (www.crpsnetworkuk.org) or the national CRPS service at the RUH. The Registry Administrator will email a study invitation letter and the Participant Information Sheet (PIS) to potential participants. If the recruitment target is not met via the CRPS UK Registry, patients of the RUH national CRPS specialist service who have previously consented to be contacted regarding research will be emailed the study invitation letter and the PIS by the researchers. Patients attending the CRPS service will also be provided with the study information by a clinician. Potential participants from the CRPS UK Registry who wish to take part will have their contact details passed to the researcher by the Registry administrator. Potential participants recruited via the national CRPS service will contact the researcher directly by email if they wish to take part. The inclusion and exclusion criteria are detailed in Table 1.

For peer review only - http://bmjopen.bmj.com/site/about/guidelines.xhtml

Table 1. Inclusion and exclusion criteria.

**Inclusion criteria**
• Adults (18+) meeting the Budapest clinical criteria (Harden *et al., 2010)* for upper or lower limb CRPS Type I.<br>• An area on their limb where a wearable band with electrode array can be attached above their painful site.<br>• An average pain level in the last 7 days rated as ≥5 at rest on a 0-10 scale.<br>• Access to appropriate technology and willingness to use this technology, that enables full study participation (a smart phone, tablet computer or computer compatible with using the video call function on Microsoft Teams, internet access, an email address, and physical and mental capacity to tolerate use of technology).<br>• A person within their household, carer or a friend to carry out the two-point discrimination measurement.
**Exclusion criteria**
• Diagnosis of any other neurological, motor disorder or major nerve damage (including CRPS Type II).<br>• Any mental health condition which may detrimentally impede study participation, in the judgement of the patient or researcher.<br>• The presence of any other limb pathology or pain on the affected CRPS limb.<br>• Poor skin condition on the area to be stimulated.<br>• Poorly controlled epilepsy.<br>• Receiving intensive CRPS specific multidisciplinary team rehabilitation in an inpatient setting during their participation in the study or within the previous month.<br>• Unable to understand written or verbal English and give informed consent.<br>• Active medical implants such as cardiac pacemakers or other devices.<br>• Exposed orthopaedic metal work in the area of electrical stimulation.<br>• Pregnancy.<br>• Known allergy to acrylates.<br>• Those living alone who have not formed a 'support bubble' with another household *(applies only if social distancing measures, as recommended by the government, are still in place, as there would be no-one able to take the two-point discrimination measurement).*

For peer review only - http://bmjopen.bmj.com/site/about/guidelines.xhtml

Recruitment and consent process

Recruitment will commence with convenience sampling; however, purposeful sampling may be required to try to achieve heterogeneity within the CRPS sample population in terms of; range of reported pain intensity, participants with upper and with lower limb CRPS, and range of CRPS duration.

Once potential participants have expressed an interest after receiving the invitation and PIS, a telephone call will be organised by the research team (JC; SG) in which they will explain the details of the study, answer any questions, confirm eligibility and participant contact details, and explain how to provide online consent using the online consent form, provided using Qualtrics (www.qualtrics.com; Qualtrics, Provo, Utah, USA). All participants will be made aware that they are free to decline participation and that doing so will not affect their usual care. If participants would like to continue, a link to the Qualtrics consent form will be emailed to the participant. The consent form will include a copy of the full PIS. Participants will be able to download a PDF copy of the completed consent form for their own records.

Participants may withdraw from the study at any point if they wish. Upon withdrawal they will be invited to provide a reason if they choose, to help inform future development of the device. It will be made clear in the consent process that any data already collected will be included in the study. Study withdrawal will not affect their continuing care. It is possible that some participants may experience local irritation at the point at which the electrodes are applied to the skin, if this continues beyond an hour after use and causes discomfort, the participant will be advised to withdraw from the study. For minor, earlier indications of skin irritation, the usage and skin advice will be reviewed, with the aim that it is resolved without becoming an issue.

Intervention design

Upon receipt of completed consent, the STS will be posted to the participant using a secure courier service and a video-call will be arranged. During the video-call, the researcher will discuss the two-point discrimination measurement with the participant. Participants will also be guided through how to access two films: a short film about the two-point discrimination measurement, and an STS instructions for use video which will show them how to unpack and set up the device and start the game-based training. Both films will be available to access through a private YouTube link. Participants will also have access to paper instructions for the use of the device and how to conduct the two-point discrimination measurements.

For peer review only - http://bmjopen.bmj.com/site/about/guidelines.xhtml

Participants will be asked to use the STS device every day for a minimum of 30 minutes a day. This can be during a single session or divided across multiple sessions over the day as determined by patient preference. To prevent concentration fatigue, it will be recommended that participants do not exceed two hours' use in any one day. To provide support to participants, they will each be contacted weekly by a member of the research team, either by email or telephone, dependent on the participant's preference.

The STS device

The STS device compromises a pattern of four electrodes placed close to, but not on, the area of allodynia using a flexible wearable band. This positioning ensures that the intensity of stimulation is tolerable to the patient and will be less likely to evoke or exacerbate their pain. The electrode array is attached to a stimulator which attaches to a tablet computer. It is anticipated that the participants will be able to attach the electrode array themselves and they will be able to set the intensity of the stimulation at the start of each session.

The tablet computer provides a game-based training environment which aims to be easy to use and engaging for participants. The STS application has a choice of two training games (Blockbuster and Flower Tower) and one relaxation activity (Music Player). The training games require attention to the limb and ask participants to identify which electrode is delivering a buzzing sensation. Each electrode is a different colour and participants are asked to respond by selecting the corresponding colour on the tablet computer. Encouraging feedback messages are given by the tablet computer throughout game play. During the relaxation activity, participants can listen to music and experience the beat of the music as a sensation through the electrodes. The Music Player involves less interaction and therefore will only be accessible after 15 minutes of game play.

Each participant will receive five different sized electrode arrays and will be able to choose which size they wish to use at the start of training after a discussion with the researcher. The choice will be based on personal preference and practicalities of anatomy. The smaller the electrode array, the closer the electrodes are to each other and therefore, the more challenging the sensory discrimination task. Participants will be able to progress through from larger to smaller templates if they are scoring well as indicated by the in-play game feedback. Participants will be asked to record which electrode size is used within a daily participant diary. Once the participant has used the device for 30 days, the device will be collected and returned by a courier at the study's cost.

For peer review only - http://bmjopen.bmj.com/site/about/guidelines.xhtml

Data collection and analysis

Participants will be asked to complete a Time 1 Questionnaire 48 hours prior to the planned video call. The questionnaire will be hosted by Qualtrics and will include date of birth, gender, which limb(s) is/are affected, duration of CRPS and current medications. The questionnaire will also include five assessments:

1. Assessment of pain intensity. A self-report rating of average pain intensity over the past seven days will be given by participants on an 11-point numerical rating scale (0-10) using the Patient Reported Outcomes Measurement Information System (PROMIS®) Numeric Rating Scale v.1.0 -Pain Intensity 1a.
2. Assessment of pain interference. A self-report rating of pain interference over the past seven days will be given by participants on an 11-point numerical rating scale (0-10). This comprises four items in the PROMIS Item Bank v1.1 – Pain Interference – Short Form 4a [25].
3. Assessment of sensitivity. A self-report rating of average sensitivity over the past seven days will be given by participants on an 11-point numerical rating scale (0-10).
4. Assessment of emotional feeling about their affected limb. A self-report rating ranging from strongly positive to strongly negative will be given by participants on an 11-point numerical rating scale (0-10). This is informed by the Bath CRPS Body Perception Disturbance scale [26].
5. The result of the sensory discrimination assessment will also be entered by the participant on to the Qualtrics survey at Time 1. The measurement will be carried out by a friend or family member who will be guided through this with a short film created by the research team, and by an information booklet. The participant will be positioned comfortably with the area to be tested exposed. A two-point discrimination threshold test using a measurement tool will be carried out close to the area of allodynia. This assessment measures the ability to discriminate sensation at two points. The smallest distance the participant can correctly identify on three occasions out of three trials, whether they were touched with one or two points, will be the recorded measurement.

At the end of the 30 days device use, the five assessments will be repeated. Participants will be sent another link via email to complete the Time 2 Questionnaire online using Qualtrics. There will be four additional assessment questions which will measure overall perception of change of pain intensity, pain interference, sensitivity, and emotional feelings towards the affected limb. All will be measured on a 7-point Likert scale ranging from 'very much improved' to 'very much worse'. The sensory

For peer review only - http://bmjopen.bmj.com/site/about/guidelines.xhtml

discrimination test will again be carried out by a family member or friend and entered into the final section of the Qualtrics survey by the participant.

The questionnaire data will be exported from Qualtrics into Microsoft Excel for the purposes of analysis. Associations between use of the STS device, changes in participants' sensory discrimination, perceived pain intensity, pain interference, sensitivity, emotional feelings about their affected limb, and self-report questionnaire data will be investigated. PROMIS interference items will be scored using the online 'Assessment Center Scoring Service' for assessment of fully anonymised data or using the raw score look-up table in the PROMIS scoring manual (Scoring Instructions (healthmeasures.net)). This service is provided under the auspices of the USA National Institute for Health. Results will be conservatively interpreted in light of the small sample size in this proof-of-concept study.

The primary research aim of investigating whether participants will adhere to using the STS device for the therapeutic dose of 30 minutes a day for 30 days will be addressed by examining data captured by the device. The frequency and duration of use for each participant will be recorded by the device.

Participants will be provided with a simple paper diary in which to record changes in array size throughout the 30 days. This will provide an option for them to record any comments on their experience.

Within two weeks of each participant completing the 30 days STS device use at home, the researcher will conduct a telephone interview to ask them about their experiences of participating in the study and of using the device. These qualitative data will be recorded with the participant's consent so that it can be transcribed verbatim. The interview schedule will be semi-structured with open-ended questions and will aim to generate qualitative data about the usability and acceptability of the device. Participants will be informed they do not have to answer any questions they do not wish to. Qualitative data from interviews with participants, will be analysed using thematic analysis [27] utilising QSR International NVivo software (2018). The summarised study design can be found in figure 1.

Adverse events

Any adverse events (AE) will be reported to the Chief Investigator and documented within the participant's study documentation. If necessary, the AE will be discussed with the research team to decide on the most appropriate action. In the unlikely event of a serious adverse event (SAE) or serious adverse reaction (SAR), the study team will immediately inform the Chief Investigator, sponsor and the MHRA. Any SAE's that are related to the study and unexpected will be reported to the Research Ethics Committee within 15 days of the Chief Investigator becoming aware of the event (as per the Health Research Authority guidance).

For peer review only - http://bmjopen.bmj.com/site/about/guidelines.xhtml

Data management

Participant identifiable information, including contact details, will only be seen by the study team. This information will only be used for the purposes of conducting the online surveys and facilitating study contact.

The e-consent and e-questionnaires will be hosted on the Qualtrics as provided and licensed by the University of the West of England, UK. Data on the tablets, which record the participants' device use, will be transferred from the tablet by the study team. The data will be deleted after each individual's participation in the study is complete. Patient confidentiality will be maintained, and data will be collected and retained in accordance with the Data Protection Act, 2018 [28].

Paper-based data will be kept securely in locked cabinets at the RUH or UWE and only accessible to the research team. All electronic personal data will be stored on RUH/UWE password-protected computer systems and managed by the research team. At the end of the study, these documents will be destroyed within six months.

Anonymised research data in the study database will be stored at UWE or RUH until the study has been analysed and written for publication. All electronic data will be destroyed within 12 months of study end. Only authorised members of the study team will have access to any electronic data. Data may also be examined by regulatory authorities to check that the study is being carried out correctly. Following the creation of the audio recording from the telephone interview, the file will be password protected and uploaded to a UWE Bristol approved and recommended transcription service. Audio recordings from telephone interviews will be password protected. Once transcribed, the audio recording will be deleted. The transcriptions will be stored in the same way as other anonymised research data.

Study monitoring

The Research and Development office at the RUH monitors projects which they sponsor on an annual basis. At UWE, a Research Governance record is audited quarterly. In addition to a UWE ethics application, UWE also require a study risk assessment to be submitted. This is held by UWE.

Patient and public involvement

Patient and public involvement (PPI) has been an integral part of the project at every stage. It was the patients' desire for a more effective and efficient means of improving sensation in their painful limbs which inspired the original idea and design of the research. Patients have continued to shape the direction of our project, and

the design of the device through its different developmental stages. Two focus groups have been held and patients strongly endorsed the importance of this device, emphasising the considerable impact CRPS has on people's lives and the need for new therapeutic approaches. Public Contributors have provided critical feedback to the study design, including recruitment and data capture processes.

Public contributors will be invited to join the Project Management Group (PMG) and will collaborate with the study team to design any patient-related documents. They will also assist in interpretation of findings and dissemination of results arising from the research. Public Contributors will contribute to the preparation of future funding applications as co-designing the device with patients has been central to this work from the outset.

ETHICAL APPROVAL AND DISSEMINATION

This study has been reviewed and given a favourable ethical opinion by London-Stanmore Research Ethics Committee, UK (ref 21/LO/0200) on 19th April 2021 and approved by the NHS Health Research Authority, UK on 7th June 2021. The Medicines and Healthcare Products Regulatory Agency provided a no grounds for objection decision on 4th June 2021. If an amendment to the study protocol is required, the Chief Investigator will ensure that a valid notice of amendment is submitted, and approval obtained.

On completion of the study a report will be prepared which will be submitted to the Research Ethics Committee and the funders, Versus Arthritis (Award Ref:22029). Dissemination will include peer reviewed publications, presentations at national/international conferences, social media, and reports for relevant patient charities (CRPS UK and Burning Nights). The funder will be acknowledged in the publications and presentations. At the end of the study, the results will be available on the CRPS UK Clinical & Research Network website, and a written summary will be available on request.

Study status: Recruitment and data collection is currently underway and will be completed in April 2023. Data analysis will take place from April 2023 until May 2023. The study is schedule to end in June 2023.

Acknowledgments: We are very grateful to Evey Watson and Krzysztof Chwiolkam, Games Technology students at UWE, who worked with MP to design the STS application used within this study; and to the CRPS UK registry who will be involved in the recruitment of participants.

Competing interests statement: The authors declare no conflicts of interest.

For peer review only - http://bmjopen.bmj.com/site/about/guidelines.xhtml

Sources of funding: This work is supported by Versus Arthritis grant number 22029.

Trial Sponsor: Professor Olena Doran, University of the West of England, Bristol, BS16 1QY, Olena.Doran@uwe.ac.uk.

Role of the trial Sponsor and funder: The sponsor and funder have no role in the study design; collection, management, analysis, and interpretation of data; writing of the report; and the decision to submit the report for publication.

Author contributions: All authors qualify for authorship in accordance with the definition provided by the International Committee of Medical Journal Editors (ICMJE). CM, DH, AL and SG conceptualised and designed the study and were involved with funding acquisition, making substantial contributions. DH and MP developed the software. All authors were involved with drafting and revising the study protocol. JC wrote the first draft of the manuscript based on the study protocol. All authors approved the final manuscript before submission.

For peer review only - http://bmjopen.bmj.com/site/about/guidelines.xhtml

References

1. Fayaz A, Croft P, Langford RM, *et al*. Prevalence of chronic pain in the UK: a systematic review and meta-analysis of population studies. *BMJ Open 2016;* 6: p.e010364. doi:10.1136/bmjopen-2015-010364
2. Dworkin RH, O'Connor AB, Audette J. Recommendations for the Pharmacological Management of Neuropathic Pain: An Overview and Literature Update. *Mayo Clinic Proceedings* 2010*;* 85: S3-S14*.* doi:10.4065/mcp.2009.0649
3. Goebel A, Barker CH, Turner-Stokes L *et al*. Complex regional pain syndrome in adults: UK guidelines for diagnosis, referral and management in primary and secondary care. London: Royal College of Physicians, 2018.
4. Phillips CJ. The cost and burden of chronic pain. *Reviews in pain* 2009; *3*: 2-5. doi: 10.1177/204946370900300102
5. Belsey J. Primary care workload in the management of chronic pain: a retrospective cohort study using a GP database to identify resource implications for UK primary care. *Journal of Medical Economics 2002;* 5: 39-50. doi: 10.3111/200205039050
6. Department of Health. Chief Medical Officer Annual Report [online]. 2009. https://webarchive.nationalarchives.gov.uk/ukgwa/20130105021744/http://www.dh.gov.uk/en/Publicationsandstatistics/Publications/AnnualReports/DH_096206 (Accessed 28 June 2022).
7. Harden RN, Bruehl S, Perez RS, *et al*. Validation of proposed diagnostic criteria (the "Budapest Criteria") for complex regional pain syndrome. *Pain* 2010; 150: 268-274. doi: 10.1016/j.pain.2010.04.030
8. Drummond PD. Sensory disturbances in Complex Regional Pain Syndrome: clinical observations, autonomic interactions, and possible mechanisms. *Pain Medicine* 2010; 11: 1257-1266. doi: 10.1111/j.1526-4637.2010.00912
9. Lewis JS, Kersten P, McCabe CS, *et al*. Body perception disturbance: a contribution to pain in Complex Regional Pain Syndrome. *Pain* 2007. 133 (1-3), 111-119. doi: 10.1016/j.pain.2007.03.013
10. Birklein F, Dimova V. Complex regional pain syndrome–up-to-date. *Pain reports* 2017; 2: e624. doi: 10.1097/PR9.0000000000000624
11. Pleger B, Ragert P, Schwenkreis P, *et al.* Patterns of cortical reorganization parallel impaired tactile discrimination and pain intensity in CRPS. *NeuroImage* 2006; 32: 503-510. doi:10.1016/j.neuroimage.2006.03.045
12. Adamczyk WM, Saulicz O, Saulicz, E, *et al*. Tactile acuity (dys)function in acute nociceptive low back pain: a double-blind experiment. *Pain 2018*; 159: 427-436. doi:10.1097/j.pain.0000000000001110

For peer review only - http://bmjopen.bmj.com/site/about/guidelines.xhtml

13. Bagg MK, Wand BM, Cashin AG, *et al*. Effect of Graded Sensorimotor Retraining on Pain Intensity in Patients With Chronic Low Back Pain: A Randomized Clinical Trial. *JAMA* 2022*;* 328: 430-439. doi:10.1001/jama.2022.9930

14. Flor H, Denke C, Schaefer M, *et al.* Effect of sensory discrimination training on cortical reorganisation and phantom limb pain. *The Lancet* 2001*;* 357: 1763-1764. doi: 10.1016/S0140-6736(00)04890.
15. Moseley GL, Zalucki NM, Wiech K. Tactile discrimination, but not tactile stimulation alone, reduces chronic limb pain. *Pain* 2008; 137:600-608. doi: 10.1016/j.pain.2007.10.021
16. Schmid AC, Schwarz A, Gustin SM, et al. Pain reduction due to novel sensory-motor training in Complex Regional Pain Syndrome I–A pilot study. Scandinavian Journal of Pain 2019; 15: 30-37. doi: 10.1016/j.sjpain.2016.11.003
17. Trapp W, Weinberger M, Erk S, *et al*. A brief intervention utilising visual feedback reduces pain and enhances tactile acuity in CLBP patients. *Journal of back and musculoskeletal rehabilitation* 2015; 28: 651-660. doi: 10.3233/BMR-140561
18. Lewis J, Coales, K, Hall J, *et al.* Now you see it, now you don't. Sensory re-education in Complex Regional Pain Syndrome. *Hand Therapy* 2011; 16: 29-38. doi: 10.1258/ht.2011.011005

19. Packham TL, Spicher CJ, MacDermid JC, *et al.* Somatosensory rehabilitation for allodynia in complex regional pain syndrome of the upper limb: A retrospective cohort study. *Journal of Hand Therapy* 2018; 3: 10-19. doi: 10.1016/j.jht.2017.02.007

20. O'Connell NE, Wand BM, Mcauley J, *et al.* Interventions for treating pain and disability in adults with complex regional pain syndrome- an overview of systematic reviews. *Cochrane Database of Systematic Reviews* 2013. doi: 10.1002/14651858.CD009416.pub2
21. Ryan CG, King R, Robinson V, *et al.* Transcutaneous electrical nerve stimulation using an LTP- like repetitive stimulation protocol for patients with upper limb complex regional pain syndrome: A feasibility study. *Hand Therapy* 2017; *22*: 52-63. doi: 10.1177/1758998316678588
22. Harris ND, Mohan D, Pleydell-Pearce CW, *et al.* Improved sensory discrimination in healthy volunteers following electrosensory discrimination training. *Clinical Rehabilitation 2010*; 24:276-287. doi: 10.1177/0269215509352055
23. McCabe C, Grieve S, Harris N. Electrical Sensory Discrimination Therapy in Complex Regional Pain Syndrome. *European Journal of Pain Supplements* 2001; 5:136. doi: 10.1016/S1754-3207(11)70465-5

For peer review only - http://bmjopen.bmj.com/site/about/guidelines.xhtml

24. Grieve S, Hart D, Harris N, *et al*. A feasibility study of the use of Electrical Sensory Discrimination Therapy for domiciliary treatment of patients with Complex Regional Pain Syndrome. Poster presented at: 5th International Congress on Neuropathic Pain; 2015 May 14-17; Nice.
25. Amtmann DA, Cook KF, Jensen MP, *et al*. Development of a PROMIS item bank to measure pain interference. Pain 2010; 150:173-182. doi:10.1016/j.pain.2010.04.025
26. Lewis J, McCabe C. Body perception disturbance in Complex Regional Pain Syndrome (CRPS). *Practical Pain Management* 2010*;* 10: 60-66.
27. Braun V, Clarke V. Using thematic analysis in psychology. *Qualitative Research in Psychology 2006*; 3: 77-101. doi:10.1191/1478088706qp063oa
28. Legislation.go.uk. Data Protection Act [online]. 2018. https://www.legislation.gov.uk/ukpga/2018/12/contents/enacted (Accessed 23 November 2022)

Figures

1. Figure 1. The STS study design.

For peer review only - http://bmjopen.bmj.com/site/about/guidelines.xhtml

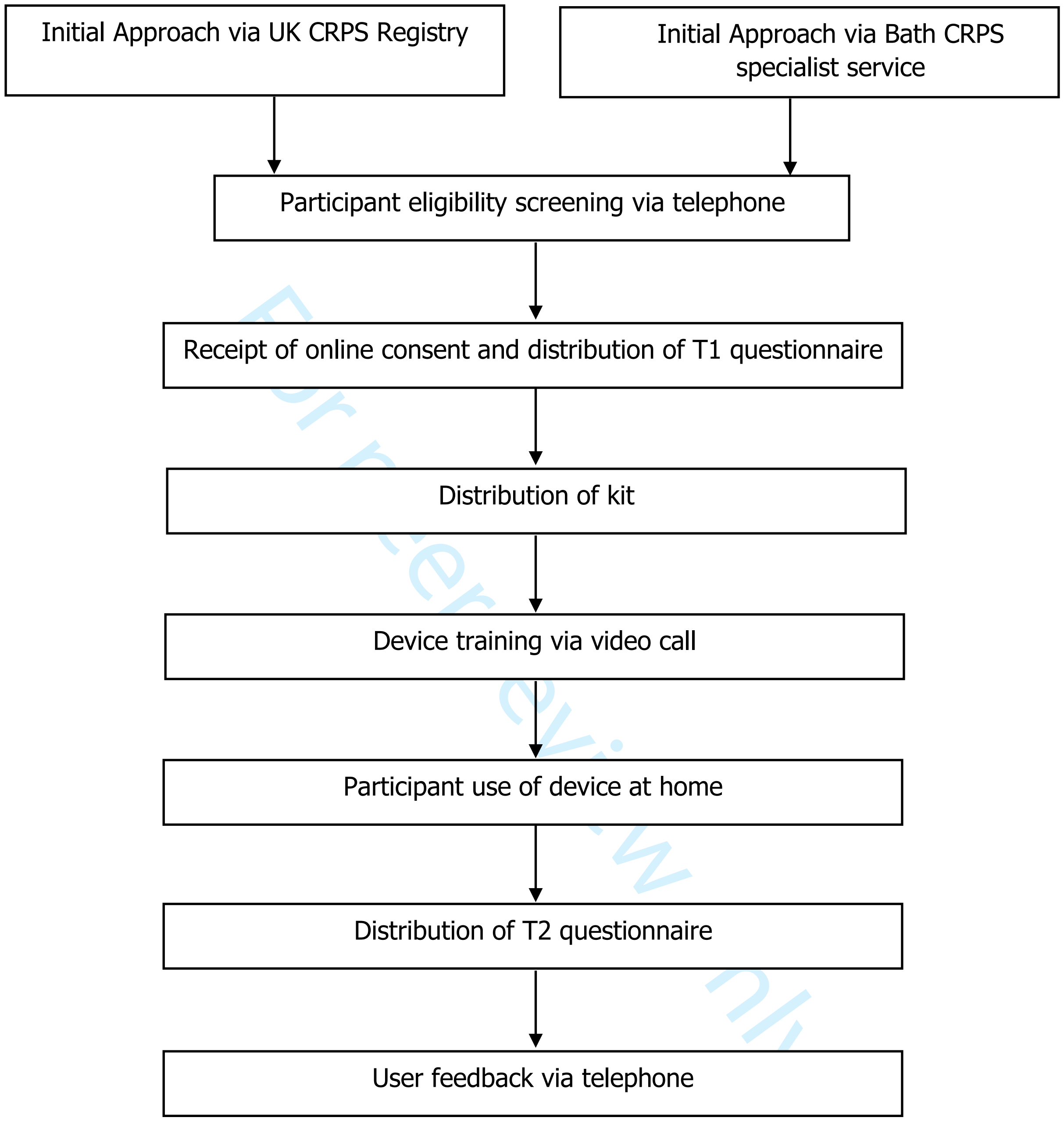

Figure 1. The STS study design.

SPIRIT
STANDARD PROTOCOL ITEMS: RECOMMENDATIONS FOR INTERVENTIONAL TRIALS

SPIRIT 2013 Checklist: Recommended items to address in a clinical trial protocol and related documents*

Section/Item	Item No	Description	Included? Page No
**Administrative information**			
Title	1	Descriptive title identifying the study design, population, interventions, and, if applicable, trial acronym	Yes - page 1
Trial registration	2a	Trial identifier and registry name. If not yet registered, name of intended registry	Yes - page 2
	2b	All items from the World Health Organization Trial Registration Data Set	Yes – throughout
Protocol version	3	Date and version identifier	Yes – page 6
Funding	4	Sources and types of financial, material, and other support	Yes – page 15
Roles and responsibilities	5a	Names, affiliations, and roles of protocol contributors	Yes – page 1
	5b	Name and contact information for the trial sponsor	Yes – page 16
	5c	Role of study sponsor and funders, if any, in study design; collection, management, analysis, and interpretation of data; writing of the report; and the decision to submit the report for publication, including whether they will have ultimate authority over any of these activities	Yes – page 16
	5d	Composition, roles, and responsibilities of the coordinating centre, steering committee, endpoint adjudication committee, data management team, and other individuals or groups overseeing the trial, if applicable (see Item 21a for data monitoring committee)	Yes – page 14
Introduction			
Background and rationale	6a	Description of research question and justification for undertaking the trial, including summary of relevant studies (published and unpublished) examining benefits and harms for each intervention	Yes – page 4

For peer review only

	6b	Explanation for choice of comparators – interviews	
Objectives	7	Specific objectives or hypotheses	Yes – page 6
Trial design	8	Description of trial design including type of trial (eg, parallel group, crossover, factorial, single group), allocation ratio, and framework (eg, superiority, equivalence, noninferiority, exploratory)	Yes – page 6
Methods: Participants, interventions, and outcomes			
Study setting	9	Description of study settings (eg, community clinic, academic hospital) and list of countries where data will be collected. Reference to where list of study sites can be obtained	Yes – page 6
Eligibility criteria	10	Inclusion and exclusion criteria for participants. If applicable, eligibility criteria for study centres and individuals who will perform the interventions (eg, surgeons, psychotherapists)	Yes – page 8 (table 1)
Interventions	11a	Interventions for each group with sufficient detail to allow replication, including how and when they will be administered	Yes – page 9 (one group only)
	11b	Criteria for discontinuing or modifying allocated interventions for a given trial participant (eg, drug dose change in response to harms, participant request, or improving/worsening disease)	Yes – page 9
	11c	Strategies to improve adherence to intervention protocols, and any procedures for monitoring adherence (eg, drug tablet return, laboratory tests)	N/A for this study
	11d	Relevant concomitant care and interventions that are permitted or prohibited during the trial	Yes – page 8 (exclusion criteria)
Outcomes	12	Primary, secondary, and other outcomes, including the specific measurement variable (eg, systolic blood pressure), analysis metric (eg, change from baseline, final value, time to event), method of aggregation (eg, median, proportion), and time point for each outcome. Explanation of the clinical relevance of chosen efficacy and harm outcomes is strongly recommended	Yes – page 11
Participant timeline	13	Time schedule of enrolment, interventions (including any run-ins and	Yes – pages 11-13

For peer review only - http://bmjopen.bmj.com/site/about/guidelines.xhtml

		washouts), assessments, and visits for participants. A schematic diagram is highly recommended (see Figure)	
Sample size	14	Estimated number of participants needed to achieve study objectives and how it was determined, including clinical and statistical assumptions supporting any sample size calculations	Yes – page 6
Recruitment	15	Strategies for achieving adequate participant enrolment to reach target sample size	Yes – page 7
**Methods: Assignment of interventions (for controlled trials)**			
Allocation:			
Sequence generation	16a	Method of generating the allocation sequence (eg, computer-generated random numbers), and list of any factors for stratification. To reduce predictability of a random sequence, details of any planned restriction (eg, blocking) should be provided in a separate document that is unavailable to those who enrol participants or assign interventions	N/A
Allocation concealment mechanism	16b	Mechanism of implementing the allocation sequence (eg, central telephone; sequentially numbered, opaque, sealed envelopes), describing any steps to conceal the sequence until interventions are assigned	N/A
Implementation	16c	Who will generate the allocation sequence, who will enrol participants, and who will assign participants to interventions	N/A
Blinding (masking)	17a	Who will be blinded after assignment to interventions (eg, trial participants, care providers, outcome assessors, data analysts), and how	N/A
	17b	If blinded, circumstances under which unblinding is permissible, and procedure for revealing a participant's allocated intervention during the trial	N/A
**Methods: Data collection, management, and analysis**			
Data collection methods	18a	Plans for assessment and collection of outcome, baseline, and other trial data, including any related processes to promote data quality (eg, duplicate measurements, training of assessors) and a description of study instruments	Yes – pages 11-12

For peer review only - http://bmjopen.bmj.com/site/about/guidelines.xhtml

		(eg, questionnaires, laboratory tests) along with their reliability and validity, if known. Reference to where data collection forms can be found, if not in the protocol	
	18b	Plans to promote participant retention and complete follow-up, including list of any outcome data to be collected for participants who discontinue or deviate from intervention protocols	Yes – page 10 (weekly contact)
Data management	19	Plans for data entry, coding, security, and storage, including any related processes to promote data quality (eg, double data entry; range checks for data values). Reference to where details of data management procedures can be found, if not in the protocol	Yes – page 12 and 14
Statistical methods	20a	Statistical methods for analysing primary and secondary outcomes. Reference to where other details of the statistical analysis plan can be found, if not in the protocol	N/A
	20b	Methods for any additional analyses (eg, subgroup and adjusted analyses)	N/A
	20c	Definition of analysis population relating to protocol non-adherence (eg, as randomised analysis), and any statistical methods to handle missing data (eg, multiple imputation)	N/A
**Methods: Monitoring**			
Data monitoring	21a	Composition of data monitoring committee (DMC); summary of its role and reporting structure; statement of whether it is independent from the sponsor and competing interests; and reference to where further details about its charter can be found, if not in the protocol. Alternatively, an explanation of why a DMC is not needed	N/A
	21b	Description of any interim analyses and stopping guidelines, including who will have access to these interim results and make the final decision to terminate the trial	N/A
Harms	22	Plans for collecting, assessing, reporting, and managing solicited and spontaneously reported adverse events	Yes – page 14

For peer review only - http://bmjopen.bmj.com/site/about/guidelines.xhtml

		and other unintended effects of trial interventions or trial conduct	
Auditing	23	Frequency and procedures for auditing trial conduct, if any, and whether the process will be independent from investigators and the sponsor	Yes – page 14
Ethics and dissemination			
Research ethics approval	24	Plans for seeking research ethics committee/institutional review board (REC/IRB) approval	Yes – page 15
Protocol amendments	25	Plans for communicating important protocol modifications (eg, changes to eligibility criteria, outcomes, analyses) to relevant parties (eg, investigators, REC/IRBs, trial participants, trial registries, journals, regulators)	Yes – page 15
Consent or assent	26a	Who will obtain informed consent or assent from potential trial participants or authorised surrogates, and how (see Item 32)	Yes – page 9
	26b	Additional consent provisions for collection and use of participant data and biological specimens in ancillary studies, if applicable	N/A

*It is strongly recommended that this checklist be read in conjunction with the SPIRIT 2013 Explanation & Elaboration for important clarification on the items. Amendments to the protocol should be tracked and dated. The SPIRIT checklist is copyrighted by the SPIRIT Group under the Creative Commons "Attribution-NonCommercial-NoDerivs 3.0 Unported" license.

For peer review only

BMJ Open

A Sensory Training System (STS) for use at home by people with Complex Regional Pain Syndrome: protocol for a proof-of-concept study

Journal:	*BMJ Open*
Manuscript ID	bmjopen-2022-070920.R1
Article Type:	Protocol
Date Submitted by the Author:	02-Mar-2023
Complete List of Authors:	Coggins, Jessica; University of the West of England, School for Health and Social Wellbeing; Royal United Hospital Bath NHS Trust, CRPS Service<br>Grieve, Sharon; Royal United Hospital Bath NHS Trust, CRPS Service; University of the West of England, School of Health and Social Wellbeing<br>Hart, Darren; Royal United Hospital Bath NHS Trust, Medical Physics & Bioengineering Department<br>Llewellyn, Alison ; University of the West of England Bristol, School of Health and Social Wellbeing; Dorothy House Hospice<br>Palmer, Mark; University of the West of England, Department of Computer Science and Creative Technologies<br>Boichat, Charlotte; The Open University, School of Psychology and Counselling<br>McCabe, Candy; University of the West of England, School of Health and Social Wellbeing; Dorothy House Hospice
**Primary Subject Heading**:	Rehabilitation medicine
Secondary Subject Heading:	Rehabilitation medicine
Keywords:	Electric Stimulation Therapy, PAIN MANAGEMENT, REHABILITATION MEDICINE, Clinical trials < THERAPEUTICS

SCHOLARONE™
Manuscripts

Title page

Title: A Sensory Training System (STS) for use at home by people with Complex Regional Pain Syndrome: protocol for a proof-of-concept study

Corresponding author: Jessica Coggins, University of the West of England, Glenside Campus, Blackberry Hill, Bristol, BS16 1DD, Jessica.Coggins@uwe.ac.uk

Author's institutional affiliations:

Jessica Coggins, School of Health and Social Wellbeing, University of the West of England, Blackberry Hill, Bristol, BS16 1DD and CRPS Service, Royal United Hospitals Bath NHS Foundation Trust, Combe Park, Bath, BA1 3NG

Dr Sharon Grieve, CRPS Service, Royal United Hospitals Bath NHS Foundation Trust, Combe Park, Bath, BA1 3NG and School of Health and Social Wellbeing, University of the West of England, Blackberry Hill, Bristol, BS16 1DD

Dr Darren Hart, Medical Physics & Bioengineering Department, Royal United Hospitals Bath NHS Foundation Trust, Combe Park, Bath, BA1 3NG

Dr Alison Llewellyn, School of Health and Social Wellbeing, University of the West of England, Blackberry Hill, Bristol, BS16 1DD and Dorothy House Hospice Care, Winsley, Bradford on Avon, BA15 2LE

Dr Mark Palmer, Department of Computer Science and Creative Technologies, University of the West of England, Coldharbour Lane, Bristol, BS16 1QY

Dr Charlotte Boichat, School of Psychology and Counselling, The Open University, Walton Hall, Milton Keynes, MK7 6AA

Professor Candida McCabe, School of Health and Social Wellbeing, University of the West of England, Blackberry Hill, Bristol, BS16 1DD and Dorothy House Hospice Care, Winsley, Bradford on Avon, BA15 2LE

Word count (excluding title page, abstract, references, acknowledgements, contributions, figures, and tables): 4438

Keywords: Complex Regional Pain Syndrome, Sensory Training System, Home use, Sensory discrimination, Electrical stimulation, Game-based therapy.

For peer review only - http://bmjopen.bmj.com/site/about/guidelines.xhtml

ABSTRACT

Introduction: Complex Regional Pain Syndrome (CRPS) is a disabling and distressing chronic pain condition characterised by a range of sensory, motor, autonomic and trophic symptoms. UK guidelines recommend therapy interventions to help normalise touch perception through self-administered tactile and thermal desensitisation activities. Interventions have been developed, aiming to help individuals broaden their sensory experience, thereby relieving chronic pain. However, therapy-led interventions often experience practical constraints and poor adherence. In response, a sensory training system (STS) device has been designed for unsupervised independent home-use.

Methods: This proof-of-concept study aims to explore whether people with CRPS use the device at home for 30 minutes a day for 30-days. Secondary aims are to determine whether the STS device will change tactile acuity and perceived levels of pain intensity, pain interference, sensitivity, or feelings towards the affected limb.

We will seek to recruit 20 eligible participants. Participants will be asked to measure tactile acuity using a two-point discrimination assessment, complete an online questionnaire before and after use of the device and complete a daily diary. Upon completion of the 30-day use, participants will be invited to take part in a semi-structured interview to explore their experiences of using the device.

Analysis: Pain intensity and pain interference will be scored using the online Assessment Center Scoring Service or using the look-up table in the PROMIS scoring manual. The remaining questionnaire data, including tactile acuity results, and device-use data, including frequency and duration of use, will be analysed using descriptive statistics. Qualitative data will be thematically analysed.

Ethics and dissemination: London-Stanmore Research Ethics Committee provided a favourable opinion on 19th April 2021 (ref 21/LO/0200). The NHS Health Research Authority, UK approved this study on 7th June 2021. Dissemination will include peer reviewed publications, presentations at conferences, social media, and reports to the funder and patient charities.

Trial registration: The trial was registered with ISRCTN registry on 28th May 2021 (ISRCTN89099843).

For peer review only

Article Summary

Strengths and limitations of this study

- The study team compromises a multidisciplinary team with varying backgrounds.
- Due to the pandemic the study was redesigned to be delivered remotely, broadening the geographic location of participants.
- Public contributors have been involved in all aspects of the study including design and monitoring.
- Medicines and Healthcare products Regulatory Agency approval gained for the clinical investigation of this non-CE marked STS device.
- The remote aspect of the study limits personal contact between researchers and participants, it also excludes individuals with limited or no access to the online technology required to participate, and excludes those who are unable to identify a second individual to complete the two-point discrimination assessment.

review only

For peer review only - http://bmjopen.bmj.com/site/about/guidelines.xhtml

INTRODUCTION

Background and rationale

Complex Regional Pain Syndrome (CRPS) is a chronic pain condition which usually develops after trauma to a limb but can occur spontaneously. CRPS often only affects one limb but can spread to involve additional limbs [1]. The condition is characterised by continuing pain dipropionate to the injury, in addition to abnormal sensory, vasomotor, sudomotor and trophic symptoms. The presence of these symptoms is used in the diagnosis of CRPS in accordance with the validated Budapest Criteria [2]. One striking feature of CRPS is allodynia; pain due to a normally innocuous stimuli, that is experienced in the affected limb. This can result in increased sensitivity to a range of stimuli, for example, clothing cannot be tolerated over the painful region. The degree of sensitivity may vary between individuals. People with CRPS often carefully protect their limb to prevent any physical contact to the allodynic site [3] and they can develop a strong dislike of the CRPS affected limb [4]. The cause of CRPS is unknown, but evidence suggests there are multiple mechanisms involved in the pathophysiology [5,6].

There is limited evidence to estimate the prevalence of CRPS within the United Kingdom, however, a study from 2007 suggests that the European incidence rate of CRPS is 26.2 per 100,000 person years [7]. CRPS occurs in approximately 4-7% of patients who experience limb fractures, injuries, or surgery [6]. For many CRPS patients, symptoms such as swelling, limb discoloration and temperature changes improve within six to 13 months, however, functional limitations persist for some for more than one year [8]. Physical impairment and severe pain continue two years after initial onset for approximately 15% of patients [9]. These populations experience high levels of disability, and care is primarily focused on reducing pain, preserving, or restoring function, and enabling patients to self-manage their condition [9].

Evidence suggests that CRPS patients experience a loss of quality of life due to reduced physical health, even more so than patients with other chronic pain conditions [10]. Alongside individual consequences, the annual economic consequences of CRPS are high [11]. This Swiss study found average treatment costs were 13 times higher, and the number of working days lost 20 times higher within the first two years after the accident in people with CRPS, compared to those without CRPS.

CRPS is associated with a decreased ability to determine the texture, temperature, and location of a stimulus to the painful area [12]. Clinically, the ability to discriminate sensations is assessed using a two-point discrimination test. Two probes

For peer review only - http://bmjopen.bmj.com/site/about/guidelines.xhtml

are applied at the same time to the person's skin, and they are asked to distinguish one or two contact points. The shorter the recorded two-probe distance, the better the sensory discrimination ability. Clinical studies have demonstrated that poor two-point discrimination is correlated with higher intensity of pain experienced and with altered brain representation of the affected body part [13, 14]. Interventions have been developed which aim to improve two-point discrimination ability, and therefore reduce pain, by normalising the brain's representation of the painful area. Effectively these interventions seek to help the individual to broaden their sensory experience, thereby 're-finding' normal sensations and relieving chronic pain [15].

Sensory discrimination training which involves cognitive effort can improve two-point discrimination, therefore, reducing patient-reported pain. This has been evidenced for electrical stimulation in amputee PLP, mechanical stimuli, and touch tasks in CRPS, and touch stimuli in chronic low back pain [15-19]. It has been suggested that sensory discrimination training requiring cognitive effort can also have a positive effect on normalising the brain's representation of the painful area [16]. UK Guidelines for adults with CRPS recommend therapy interventions to help normalise touch perception via self-administered tactile and thermal desensitisation activities [9]. Current sensory discrimination training involves repetitive, manual application of different textiles or stimuli to the 'affected', and an 'unaffected' body site, normally by a therapist or carer [20, 21]. However, therapy is slow to deliver improvements, and adherence is poor with limited evidence for any therapist-led interventions that deliver sustained clinically important differences in the long term [22].

Mindful of the practical constraints of the NHS and poor adherence to sensory discrimination training in practice, a home-based sensory discrimination training intervention has been developed by the research team. It is essential that any device created for this purpose is well-developed, reliable, and suitable for unsupervised independent use to gain the expected benefit. A recent study explored the feasibility and acceptability of a novel home-based sensory perception training game for patients with fibromyalgia [23]. Results from this study suggest that a home-based device with a games element is engaging, satisfying and has high adherence. There are commercially available devices that passively stimulate areas of the body but do not include a training element which actively requires cognitive effort in combination with an electrical stimulus. The training element is suggested to be an essential factor in improving two-point discrimination and pain [17, 24].

Our device consists of a commercially available stimulator, tablet computer and wearable arm and leg bands. The bands were adapted for the study to comfortably house an electrode array against the participants' skin. The electrode array comprises four colour coded electrodes, with five different array sizes to enable the difficult of the sensory-discrimination task to be increased. The electrodes are

For peer review only - http://bmjopen.bmj.com/site/about/guidelines.xhtml

represented on the tablet screen as four colour coded images that match the location of the colour coded electrodes in the array. The participant has the option to play one of two games or use the music player as a relaxation activity.

This current research project builds on 14 years of research and development, throughout which the authors have worked with people with CRPS to design a prototype sensory training system (STS) for use at home to improve tactile acuity [25, 26, 27]. The overall goal is to develop a commercially available, clinically effective STS for independent home use by people who have a persistent limb pain, so they can re-gain normal limb sensation. This protocol describes the clinical testing of the next generation STS, that comprises a customised wearable electrode array and game-based training environment, which provides an easy to use and engaging electrical STS for use in the home. The device has received Medicines & Healthcare products Regulatory Agency (MHRA; ref: CI/2021/0019/GB) approval.

Research aim and objectives

This study aims to collect pilot clinical data on a sample of CRPS patients to determine STS duration of use over a 30-day period, and its potential relationship with two-point discrimination and pain intensity.

The primary objective is to explore whether people with CRPS will use the STS device in their own homes adhering to the treatment plan of 30 minutes a day for 30 days (the therapeutic dose). Electrical stimulation of 30 minutes per day for a four-week period has been shown in a PLP population to deliver measurable improvements in pain intensity and changes on cortical body maps towards normalisation [16]. The secondary objectives are:

- To determine whether use of the STS device at the therapeutic dose changes tactile acuity (measured using two-point discrimination).
- To determine whether use of the STS device at the therapeutic dose affects perceived levels of pain intensity, pain interference, sensitivity, or feelings towards the affected limb.
- To establish whether participants have found using the STS device to be acceptable and feasible in terms of setting up the device, attaching and detaching the electrode array by themselves.
- To establish whether participants have found using the STS device to be sufficiently engaging to encourage adherence to the therapeutic dose.

METHODOLOGY

This protocol is version 5.0 dated 24th February 2022. Recruitment commenced in September 2021 and is due to close in April 2023.

For peer review only - http://bmjopen.bmj.com/site/about/guidelines.xhtml

Study design

This is a proof-of-concept study designed to explore the acceptability and useability of a customised STS.

Study setting

In response to the COVID-19 pandemic, this study will be conducted remotely across the United Kingdom. This is a single centre study based at the Royal United Hospitals Bath NHS Foundation Trust (RUH), in collaboration with the University of the West of England (UWE).

Participants and sample size

The study aims to recruit 20 adult participants who meet the inclusion criteria and who are willing to use the STS in their homes for 30 minutes per day for 30 days. However, due to the severity of symptoms experienced with CRPS and the common occurrence of 'flares' of symptoms, we anticipate a number of withdrawals. All data will be reported, including incomplete data sets and reasons for withdrawal, if provided.

Evidence suggests that the Budapest criteria is the preferred diagnostic tool for adults with CRPS [2, 28] , therefore, adults meeting this criteria will be recruited from the CRPS UK Registry (www.crpsnetworkuk.org) or the national CRPS service at the RUH. The Registry Administrator will email a study invitation letter and the Participant Information Sheet (PIS) to potential participants. If the recruitment target is not met via the CRPS UK Registry, patients of the RUH national CRPS specialist service who have previously consented to be contacted regarding research will be emailed the study invitation letter and the PIS by the researchers. Patients attending the CRPS service will also be provided with the study information by a clinician. Potential participants from the CRPS UK Registry who wish to take part will have their contact details passed to the researcher by the Registry administrator. Potential participants recruited via the national CRPS service will contact the researcher directly by email if they wish to take part. The inclusion and exclusion criteria are detailed in Table 1.

For peer review only - http://bmjopen.bmj.com/site/about/guidelines.xhtml

Table 1. Inclusion and exclusion criteria.

**Inclusion criteria**
• Adults (18+) meeting the Budapest clinical criteria [2] for upper or lower limb CRPS Type I.<br>• An area on their limb where a wearable band with electrode array can be attached above their painful site.<br>• An average pain level in the last 7 days rated as ≥5 at rest on a 0-10 scale.<br>• Access to appropriate technology and willingness to use this technology, that enables full study participation (a smart phone, tablet computer or computer compatible with using the video call function on Microsoft Teams, internet access, an email address, and physical and mental capacity to tolerate use of technology).<br>• A person within their household, carer or a friend to carry out the two-point discrimination measurement.
**Exclusion criteria**
• Diagnosis of any other neurological, motor disorder or major nerve damage (including CRPS Type II).<br>• Any mental health condition which may detrimentally impede study participation, in the judgement of the patient or researcher.<br>• The presence of any other limb pathology or pain on the affected CRPS limb.<br>• Poor skin condition on the area to be stimulated.<br>• Poorly controlled epilepsy.<br>• Receiving intensive CRPS specific multidisciplinary team rehabilitation in an inpatient setting during their participation in the study or within the previous month.<br>• Unable to understand written or verbal English and give informed consent.<br>• Active medical implants such as cardiac pacemakers or other devices.<br>• Exposed orthopaedic metal work in the area of electrical stimulation.<br>• Pregnancy.<br>• Known allergy to acrylates which are present in some device components.<br>• Those living alone who have not formed a ‘support bubble’ with another household *(applies only if social distancing measures, as recommended by the government, are still in place, as there would be no-one able to take the two-point discrimination measurement).*

For peer review only - http://bmjopen.bmj.com/site/about/guidelines.xhtml

Recruitment and consent process

Recruitment will commence with convenience sampling; however, purposeful sampling may be required to try to achieve heterogeneity within the CRPS sample population in terms of; range of reported pain intensity, participants with upper and with lower limb CRPS, and range of CRPS duration.

Once potential participants have expressed an interest after receiving the invitation and PIS, a telephone call will be organised by the research team (JC; SG) in which they will explain the details of the study, answer any questions, confirm eligibility and participant contact details, and explain how to provide online consent using the online consent form, provided using Qualtrics [29]. All participants will be made aware that they are free to decline participation and that doing so will not affect their usual care. If participants would like to continue, a link to the Qualtrics consent form will be emailed to the participant. The consent form will include a copy of the full PIS. Participants will be able to download a PDF copy of the completed consent form for their own records.

Participants may withdraw from the study at any point if they wish. Upon withdrawal they will be invited to provide a reason if they choose, to help inform future development of the device. It will be made clear in the consent process that any data already collected will be included in the study. Study withdrawal will not affect their continuing care. It is possible that some participants may experience local irritation at the point at which the electrodes are applied to the skin, if this continues beyond an hour after use and causes discomfort, the participant will be advised to withdraw from the study. For minor, earlier indications of skin irritation, the usage and skin advice will be reviewed, with the aim that it is resolved without becoming an issue.

Intervention design

Upon receipt of completed consent, the STS will be posted to the participant using a secure courier service and a video-call will be arranged. During the video-call, the researcher will discuss the two-point discrimination assessment with the participant. Participants will also be guided through how to access two films: a short film about the two-point discrimination assessment, and an STS instructions for use video which will show them how to unpack and set up the device and start the game-based training. Both films will be available to access through a private YouTube link. Participants will also have access to paper instructions for the use of the device and how to conduct the two-point discrimination measurements.

For peer review only - http://bmjopen.bmj.com/site/about/guidelines.xhtml

Participants will be asked to use the STS device every day for a minimum of 30 minutes a day. This can be during a single session or divided across multiple sessions over the day as determined by patient preference. To prevent concentration fatigue, it will be recommended that participants do not exceed two hours' use in any one day. To provide support to participants, they will each be contacted weekly by a member of the research team, either by email or telephone, dependent on the participant's preference.

The STS device

The STS device (figure 1) compromises a pattern of four electrodes placed close to, but not on, the area of allodynia using a flexible wearable band. This positioning ensures that the intensity of stimulation is tolerable to the patient and will be less likely to evoke or exacerbate their pain. The electrode array is attached to a stimulator which connects to a tablet computer. The participant uses the computer tablet to set the level of stimulation that each electrodes emits, to the point they can feel the sensation, but it is not painful. The participant can set the current between 1-50mA and the frequency at either 10Hz (slow), 25Hz (medium) and 40Hz (fast) depending on their preference. The burst duration of the stimulation will be between 900-925ms dependent upon the frequency setting. It is anticipated that the participants will be able to attach the electrode array themselves and they will be able to set the intensity of the stimulation when they first use the device and adjust this at the start of each session if required.

The tablet computer provides a game-based training environment which aims to be easy to use and engaging for participants. The STS application has a choice of two training games (Blockbuster and Flower Tower) and one relaxation activity (Music Player). Each electrode is a different colour and participants are asked to respond by selecting the corresponding colour on the tablet computer. Flower Tower will ask the participant to accurately recognise which electrode is being stimulated. Blockbuster includes five separate levels, participants will be asked to identify which electrode is being stimulated and in more challenging levels, accurately recognise different patterns of stimulation across the array. Difficulty in the tasks is provided by the stimulation of more than one electrode at any one time, stimulating electrodes that are located adjacent to each other, or complexity of the pattern of stimulation. Encouraging feedback messages are given by the tablet computer throughout game play. During the relaxation activity, participants can listen to music and experience the beat of the music as a sensation through the electrodes. The Music Player involves less interaction and therefore will only be accessible after 15 minutes of game play.

For peer review only - http://bmjopen.bmj.com/site/about/guidelines.xhtml

Each participant will receive five different sized electrode arrays and will be able to choose which size they wish to use at the start of training after a discussion with the researcher. The choice will be based on personal preference and practicalities of anatomy. The smaller the electrode array, the closer the electrodes are to each other and therefore, the more challenging the sensory discrimination task. Participants will be able to progress through from larger to smaller templates if they are scoring well as indicated by the in-play game feedback. If the participant achieves between 50-70% accuracy, the device will suggest that this is an appropriate level and that they are using an appropriately sized electrode array. If participants achieve above 70% on multiple occasions, the feedback message will encourage them to consider trying a smaller electrode or moving to the next level. Participants will be asked to record which electrode size is used within a daily participant diary. Once the participant has used the device for 30 days, the device will be collected and returned by a courier at the study's cost.

Data collection and analysis

Participants will be asked to complete a Time 1 Questionnaire 48 hours prior to the planned video call. The questionnaire will be hosted by Qualtrics and will include date of birth, gender, which limb(s) is/are affected, duration of CRPS and current medications. The questionnaire will also include five assessments:

1. Assessment of pain intensity. A self-report rating of average pain intensity over the past seven days will be given by participants on an 11-point numerical rating scale (0-10) using the Patient Reported Outcomes Measurement Information System (PROMIS®) Numeric Rating Scale v.1.0 -Pain Intensity 1a.
2. Assessment of pain interference. A self-report rating of pain interference over the past seven days will be given by participants on an 11-point numerical rating scale (0-10). This comprises four items in the PROMIS Item Bank v1.1 – Pain Interference – Short Form 4a [30].
3. Assessment of sensitivity. A self-report rating of average sensitivity over the past seven days will be given by participants on an 11-point numerical rating scale (0-10). In clinical practice, this patient population would commonly be asked about the degree of sensitivity they are experiencing in their affected limb. Patients understanding of this would be how tolerant they are to touch and/or temperature changes within that limb. They would be very familiar with this type of question. Our patient research partners were consulted about all study documentation and approved the wording of this question.

For peer review only - http://bmjopen.bmj.com/site/about/guidelines.xhtml

4. Assessment of emotional feeling about their affected limb. A self-report rating ranging from strongly positive to strongly negative will be given by participants on an 11-point numerical rating scale (0-10). This is informed by the Bath CRPS Body Perception Disturbance scale [31].
5. The result of the sensory discrimination assessment will also be entered by the participant on to the Qualtrics survey at Time 1. This assessment measures the ability to discriminate sensation at two points. The smallest distance the participant can correctly identify on three occasions out of two trials, whether they were touched with one or two points, will be the recorded measurement. The measurement will be carried out by a friend or family member who will be guided through this with a short film created by the research team, and by an information booklet. The participant will be positioned comfortably with the area to be tested exposed and their eyes closed. A two-point discrimination threshold test using a plastic two-point aesthesiometer will be carried out close to the area of allodynia. Each tool has five different coloured marks, each 2cms apart, which determine when the aesthesiometer is at 1cm, 3cm, 5cm, 7cm and 9cm. The person conducting the measurement, starts at 9cm and reduces this according to whether the participant can accurately discriminate between one and two points against their skin. This use of colour codes was designed to simplify the two-point discrimination task for the friend or family member and to reduce the possibility of measurement errors. Ideally, this assessment would have been conducted by a member of the research team but due to COVID-19 requires changes in the study, this pragmatic solution was created. The individual conducting the assessment is also asked to note the anatomical location of the assessment so this can be used again at Time 2.

At the end of the 30 days device use, the five assessments will be repeated. Participants will be sent another link via email to complete the Time 2 Questionnaire online using Qualtrics. There will be four additional assessment questions which will measure overall perception of change of pain intensity, pain interference, sensitivity, and emotional feelings towards the affected limb. All will be measured on a 7-point Likert scale ranging from 'very much improved' to 'very much worse'. The sensory discrimination test will again be carried out by a family member or friend and entered into the final section of the Qualtrics survey by the participant.

The questionnaire data will be exported from Qualtrics into Microsoft Excel for the purposes of analysis. Associations between use of the STS device, changes in participants' sensory discrimination, perceived pain intensity, pain interference, sensitivity, emotional feelings about their affected limb, and self-report questionnaire

For peer review only - http://bmjopen.bmj.com/site/about/guidelines.xhtml

data will be investigated. PROMIS interference items will be scored using the online 'Assessment Center Scoring Service' for assessment of fully anonymised data or using the raw score look-up table in the PROMIS scoring manual (https://www.healthmeasures.net/). This service is provided under the auspices of the USA National Institute for Health. Results will be conservatively interpreted in light of the small sample size in this proof-of-concept study.

The primary research aim of investigating whether participants will adhere to using the STS device for the therapeutic dose of 30 minutes a day for 30 days will be addressed by examining data captured by the device. The frequency and duration of active game use for each participant will be recorded by the device and the research team will be able to view this data once the device has been mailed back to the research team.

Participants will be provided with a simple paper diary in which to record changes in array size throughout the 30 days. This will provide an option for them to record any comments on their experience.

Within two weeks of each participant completing the 30 days STS device use at home, the researcher will conduct a telephone interview to ask them about their experiences of participating in the study and of using the device. These qualitative data will be recorded with the participant's consent so that it can be transcribed verbatim. The interview schedule will be semi-structured with open-ended questions and will aim to generate qualitative data about the usability and acceptability of the device. Participants will be informed they do not have to answer any questions they do not wish to. Qualitative data from interviews with participants, will be analysed using thematic analysis [32] utilising QSR International NVivo software (2018). The summarised study design can be found in figure 2.

For peer review only

Adverse events

Any adverse events (AE) will be reported to the Chief Investigator and documented within the participant's study documentation. If necessary, the AE will be discussed with the research team to decide on the most appropriate action. In the unlikely event of a serious adverse event (SAE) or serious adverse reaction (SAR), the study team will immediately inform the Chief Investigator, sponsor and the MHRA. Any SAE's that are related to the study and unexpected will be reported to the Research Ethics Committee within 15 days of the Chief Investigator becoming aware of the event (as per the Health Research Authority guidance).

Data management

For peer review only - http://bmjopen.bmj.com/site/about/guidelines.xhtml

Participant identifiable information, including contact details, will only be seen by the study team. This information will only be used for the purposes of conducting the online surveys and facilitating study contact.

The e-consent and e-questionnaires will be hosted on the Qualtrics as provided and licensed by the University of the West of England, UK. Data on the tablets, which record the participants' device use, will be transferred from the tablet by the study team. The data will be deleted after each individual's participation in the study is complete. Patient confidentiality will be maintained, and data will be collected and retained in accordance with the Data Protection Act, 2018 [33].

Paper-based data will be kept securely in locked cabinets at the RUH or UWE and only accessible to the research team. All electronic personal data will be stored on RUH/UWE password-protected computer systems and managed by the research team. At the end of the study, these documents will be destroyed within six months.

Anonymised research data in the study database will be stored at UWE or RUH until the study has been analysed and written for publication. All electronic data will be destroyed within 12 months of study end. Only authorised members of the study team will have access to any electronic data. Data may also be examined by regulatory authorities to check that the study is being carried out correctly. Following the creation of the audio recording from the telephone interview, the file will be password protected and uploaded to a UWE Bristol approved and recommended transcription service. Audio recordings from telephone interviews will be password protected. Once transcribed, the audio recording will be deleted. The transcriptions will be stored in the same way as other anonymised research data.

Study monitoring

The Research and Development office at the RUH monitors projects which they sponsor on an annual basis. At UWE, a Research Governance record is audited quarterly. In addition to a UWE ethics application, UWE also require a study risk assessment to be submitted. This is held by UWE.

Patient and public involvement

Patient and public involvement (PPI) has been an integral part of the project at every stage. It was the patients' desire for a more effective and efficient means of improving sensation in their painful limbs which inspired the original idea and design of the research. Patients have continued to shape the direction of our project, and the design of the device through its different developmental stages. Two focus groups have been held and patients strongly endorsed the importance of this device,

For peer review only - http://bmjopen.bmj.com/site/about/guidelines.xhtml

emphasising the considerable impact CRPS has on people's lives and the need for new therapeutic approaches. Public Contributors have provided critical feedback to the study design, including recruitment and data capture processes.

Public contributors will be invited to join the Project Management Group (PMG) and will collaborate with the study team to design any patient-related documents. They will also assist in interpretation of findings and dissemination of results arising from the research. Public Contributors will contribute to the preparation of future funding applications as co-designing the device with patients has been central to this work from the outset.

ETHICAL APPROVAL AND DISSEMINATION

This study has been reviewed and given a favourable ethical opinion by London-Stanmore Research Ethics Committee, UK (ref 21/LO/0200) on 19th April 2021 and approved by the NHS Health Research Authority, UK on 7th June 2021. The Medicines and Healthcare Products Regulatory Agency provided a no grounds for objection decision on 4th June 2021. If an amendment to the study protocol is required, the Chief Investigator will ensure that a valid notice of amendment is submitted, and approval obtained.

On completion of the study a report will be prepared which will be submitted to the Research Ethics Committee and the funders, Versus Arthritis (Award Ref:22029). Dissemination will include peer reviewed publications, presentations at national/international conferences, social media, and reports for relevant patient charities (CRPS UK and Burning Nights). The funder will be acknowledged in the publications and presentations. At the end of the study, the results will be available on the CRPS UK Clinical & Research Network website, and a written summary will be available on request.

Study status: Recruitment and data collection is currently underway and will be completed in April 2023. Data analysis will take place from April 2023 until May 2023. The study is schedule to end in June 2023.

Acknowledgments: We are very grateful to Evey Watson and Krzysztof Chwiolkam, Games Technology students at UWE, who worked with MP to design the STS application used within this study; and to the CRPS UK registry who will be involved in the recruitment of participants.

Competing interests statement: The authors declare no conflicts of interest.

Sources of funding: This work is supported by Versus Arthritis grant number 22029.

For peer review only - http://bmjopen.bmj.com/site/about/guidelines.xhtml

Trial Sponsor: Professor Olena Doran, University of the West of England, Bristol, BS16 1QY, Olena.Doran@uwe.ac.uk.

Role of the trial Sponsor and funder: The sponsor and funder have no role in the study design; collection, management, analysis, and interpretation of data; writing of the report; and the decision to submit the report for publication.

Author contributions: All authors qualify for authorship in accordance with the definition provided by the International Committee of Medical Journal Editors (ICMJE). CM, DH, AL and SG conceptualised and designed the study and were involved with funding acquisition, making substantial contributions. DH and MP developed the software. CB and SG initially drafted the protocol, and all authors were subsequently involved with revising and agreeing the final version. JC wrote the first draft of the manuscript based on the study protocol. All authors approved the final manuscript before submission.

References

1. van Rijn MA, Marinus J, Putter H, *et al*. Spreading of complex regional pain syndrome: Not a random process. *Journal of Neural Transmission* 2011; 118, 1301–1309. DOI 10.1007/s00702-011-0601-1
2. Harden RN, Bruehl S, Perez RS, *et al*. Validation of proposed diagnostic criteria (the "Budapest Criteria") for complex regional pain syndrome. *Pain* 2010; 150: 268-274. doi: 10.1016/j.pain.2010.04.030
3. Drummond PD. Sensory disturbances in Complex Regional Pain Syndrome: clinical observations, autonomic interactions, and possible mechanisms. *Pain Medicine* 2010; 11: 1257-1266. doi: 10.1111/j.1526-4637.2010.00912
4. Lewis JS, Kersten P, McCabe CS, *et al*. Body perception disturbance: a contribution to pain in Complex Regional Pain Syndrome. *Pain* 2007. 133 (1-3), 111-119. doi: 10.1016/j.pain.2007.03.013
5. Marinus J, Moseley GL, Birklein F et al. Clinical features and pathophysiology of complex regional pain syndrome. *Lancet Neurol* 2011;10: 637–48. doi: https://doi.org/10.1016/S1474-4422(11)70106-5
6. Bruehl S. Complex regional pain syndrome. *BMJ* 2015; 351. doi: 10.1136/bmj.h2730
7. de Mos M, de Bruijn AG, Huygen FJ, Dieleman JP, Stricker BH, Sturkenboom MC. The incidence of complex regional pain syndrome: a population-based study. *Pain.* 2007;129: 12–20. doi: 10.1016/j.pain.2006.09.008.
8. Bean, DJ, Johnson, MH, Kydd RR. The outcome of complex regional pain syndrome type 1: A systematic review. *The Journal of Pain 2014; 15: 677-690.*

For peer review only - http://bmjopen.bmj.com/site/about/guidelines.xhtml

9. Goebel A, Barker CH, Turner-Stokes L *et al.* Complex regional pain syndrome in adults: UK guidelines for diagnosis, referral and management in primary and secondary care. London: Royal College of Physicians, 2018.
10. van Velzen GA, Perez RS, van Gestel MA, *et al.* Health-related quality of life in 975 patients with complex regional pain syndrome type 1. PAIN®. 2014; 155:629-34. doi: 10.1016/j.pain.2013.12.017.
11. Scholz-Odermatt SM, Luthi F, Wertli MM, *et al.* Direct Health Care Cost and Work Incapacity Related to Complex Regional Pain Syndrome in Switzerland: A Retrospective Analysis from 2008 to 2015. *Pain Medicine*. 2019; 20: 1559–1569. doi: 10.1093/pm/pnz030
12. Birklein F, Dimova V. Complex regional pain syndrome–up-to-date. *Pain reports* 2017; 2: e624. doi: 10.1097/PR9.0000000000000624
13. Pleger B, Ragert P, Schwenkreis P, *et al.* Patterns of cortical reorganization parallel impaired tactile discrimination and pain intensity in CRPS. *NeuroImage* 2006; 32: 503-510. doi:10.1016/j.neuroimage.2006.03.045
14. Adamczyk WM, Saulicz O, Saulicz E, *et al.* Tactile acuity (dys)function in acute nociceptive low back pain: a double-blind experiment. *Pain 2018*; 159: 427-436. doi:10.1097/j.pain.0000000000001110
15. Bagg MK, Wand BM, Cashin AG, *et al.* Effect of Graded Sensorimotor Retraining on Pain Intensity in Patients With Chronic Low Back Pain: A Randomized Clinical Trial. *JAMA* 2022*;* 328: 430-439. doi:10.1001/jama.2022.9930
16. Flor H, Denke C, Schaefer M, *et al.* Effect of sensory discrimination training on cortical reorganisation and phantom limb pain. *The Lancet* 2001*;* 357: 1763-1764. doi: 10.1016/S0140-6736(00)04890.
17. Moseley GL, Zalucki NM, Wiech K. Tactile discrimination, but not tactile stimulation alone, reduces chronic limb pain. *Pain* 2008; 137:600-608. doi: 10.1016/j.pain.2007.10.021
18. Schmid AC, Schwarz A, Gustin SM, *et al.* Pain reduction due to novel sensory-motor training in Complex Regional Pain Syndrome I–A pilot study. *Scandinavian Journal of Pain* 2019; 15: 30-37. doi: 10.1016/j.sjpain.2016.11.003
19. Trapp W, Weinberger M, Erk S, *et al.* A brief intervention utilising visual feedback reduces pain and enhances tactile acuity in CLBP patients. *Journal of back and musculoskeletal rehabilitation* 2015; 28: 651-660. doi: 10.3233/BMR-140561
20. Lewis J, Coales, K, Hall J, *et al.* Now you see it, now you don't. Sensory re-education in Complex Regional Pain Syndrome. *Hand Therapy* 2011; 16: 29-38. doi: 10.1258/ht.2011.011005
21. Packham TL, Spicher CJ, MacDermid JC, *et al.* Somatosensory rehabilitation for allodynia in complex regional pain syndrome of the upper limb: A retrospective cohort study. *Journal of Hand Therapy* 2018; 3: 10-19. doi: 10.1016/j.jht.2017.02.007
22. O'Connell NE, Wand BM, Mcauley J, *et al.* Interventions for treating pain and disability in adults with complex regional pain syndrome- an overview of

For peer review only - http://bmjopen.bmj.com/site/about/guidelines.xhtml

systematic reviews. *Cochrane Database of Systematic Reviews* 2013; 4. doi: 10.1002/14651858.CD009416.pub2
23. Demoulin C, Labory C, Marcon C, *et al*. Feasibility and Acceptability of a Home-Based Sensory Perception Training Game for Patients with Fibromyalgia: A Pilot Study. *Games for Health Journal* 2023; 12. doi: 10.1089/g4h.2022.0079
24. Ryan CG, King R, Robinson V, *et al.* Transcutaneous electrical nerve stimulation using an LTP- like repetitive stimulation protocol for patients with upper limb complex regional pain syndrome: A feasibility study. *Hand Therapy* 2017; *22*: 52-63. doi: 10.1177/1758998316678588
25. Harris ND, Mohan D, Pleydell-Pearce CW, et al. Improved sensory discrimination in healthy volunteers following electrosensory discrimination training. *Clinical Rehabilitation* 2010; 24:276-287. doi: 10.1177/0269215509352055
26. McCabe C, Grieve S, Harris N. Electrical Sensory Discrimination Therapy in Complex Regional Pain Syndrome. *European Journal of Pain Supplements* 2001; 5:136. doi: 10.1016/S1754-3207(11)70465-5
27. Grieve S, Hart D, Harris N, *et al*. Electrical sensory discrimination therapy in Complex Regional Pain Syndrome. Poster presented at: 5th International Congress on Neuropathic Pain; 2015 May 14-17; Nice.
28. Mesaroli G, Hundert A, Birnie KA, *et al.* Screening and diagnostic tools for complex regional pain syndrome: a systematic review. *Pain* 2021; 162: 1295. doi: 10.1097/j.pain.0000000000002146
29. Qualtrics software *Copyright © 2022 Qualtrics. Qualtrics and all other Qualtrics product or service names are registered trademarks or trademarks of Qualtrics*. Provo, UT, USA. https://www.qualtrics.com
30. Amtmann DA, Cook KF, Jensen MP, *et al*. Development of a PROMIS item bank to measure pain interference. Pain 2010; 150:173-182. doi:10.1016/j.pain.2010.04.025
31. Lewis J, McCabe C. Body perception disturbance in Complex Regional Pain Syndrome (CRPS). *Practical Pain Management* 2010*;* 10: 60-66.
32. Braun V, Clarke V. Using thematic analysis in psychology. *Qualitative Research in Psychology 2006*; 3: 77-101. doi:10.1191/1478088706qp063oa
33. Legislation.go.uk. Data Protection Act [online]. 2018. https://www.legislation.gov.uk/ukpga/2018/12/contents/enacted (Accessed 23 November 2022)

Figures

1. Figure 1. The STS device in use by a member of the research team.
2. Figure 2. The STS study design.

For peer review only - http://bmjopen.bmj.com/site/about/guidelines.xhtml

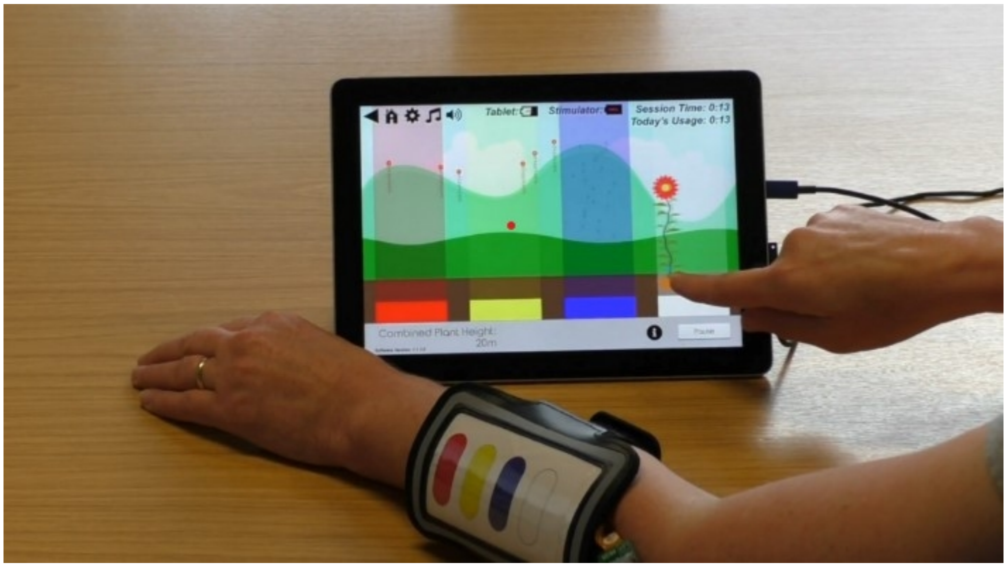

Figure 1. The STS device in use by a member of the research team.

125x69mm (144 x 144 DPI)

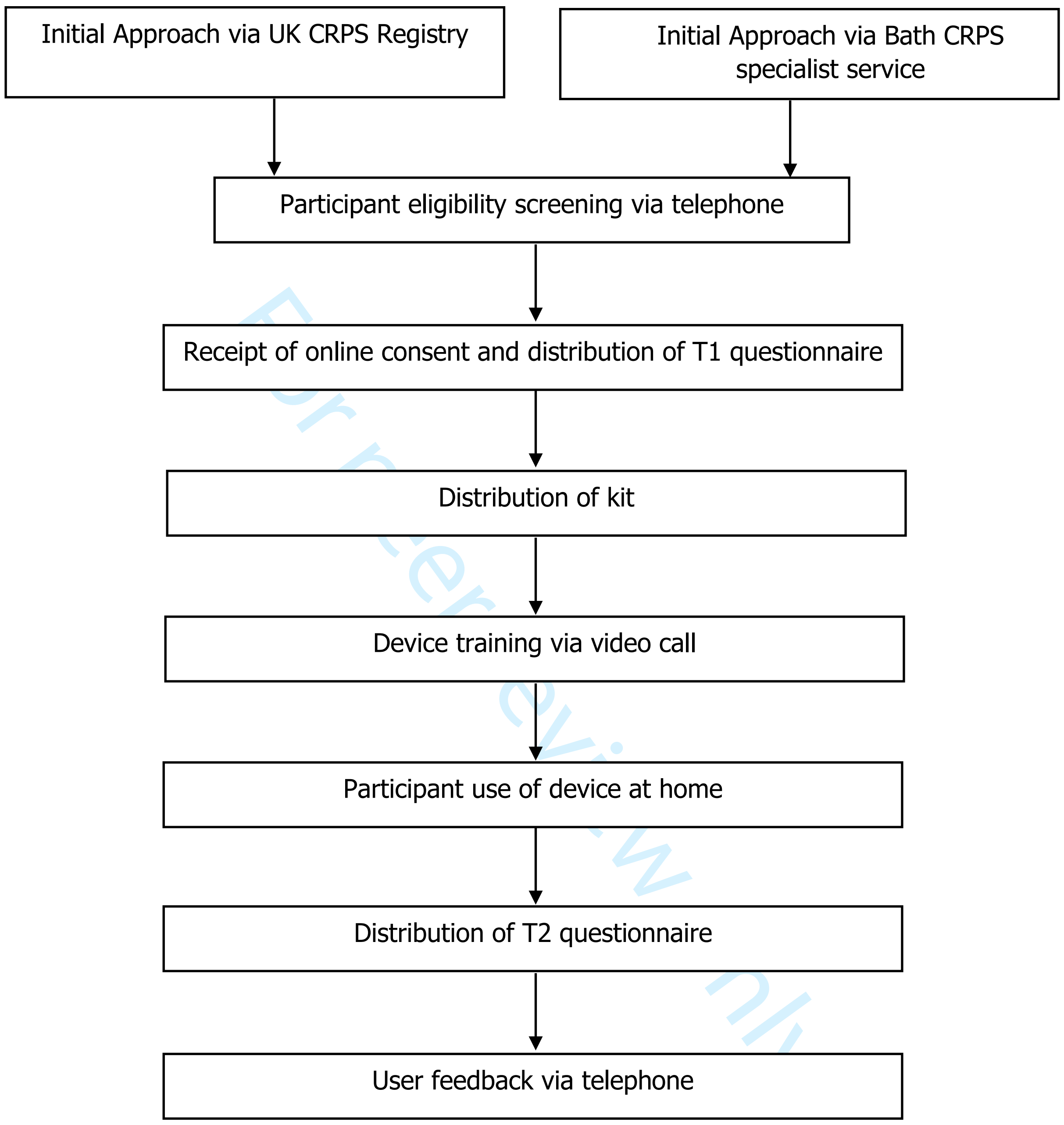

Figure 2. The STS study design.

For peer review only - http://bmjopen.bmj.com/site/about/guidelines.xhtml

SPIRIT

STANDARD PROTOCOL ITEMS: RECOMMENDATIONS FOR INTERVENTIONAL TRIALS

SPIRIT 2013 Checklist: Recommended items to address in a clinical trial protocol and related documents*

Section/Item	Item No	Description	Included? Page No
**Administrative information**			
Title	1	Descriptive title identifying the study design, population, interventions, and, if applicable, trial acronym	Yes - page 1
Trial registration	2a	Trial identifier and registry name. If not yet registered, name of intended registry	Yes - page 2
	2b	All items from the World Health Organization Trial Registration Data Set	Yes – throughout
Protocol version	3	Date and version identifier	Yes – page 6
Funding	4	Sources and types of financial, material, and other support	Yes – page 15
Roles and responsibilities	5a	Names, affiliations, and roles of protocol contributors	Yes – page 1
	5b	Name and contact information for the trial sponsor	Yes – page 16
	5c	Role of study sponsor and funders, if any, in study design; collection, management, analysis, and interpretation of data; writing of the report; and the decision to submit the report for publication, including whether they will have ultimate authority over any of these activities	Yes – page 16
	5d	Composition, roles, and responsibilities of the coordinating centre, steering committee, endpoint adjudication committee, data management team, and other individuals or groups overseeing the trial, if applicable (see Item 21a for data monitoring committee)	Yes – page 14
Introduction			
Background and rationale	6a	Description of research question and justification for undertaking the trial, including summary of relevant studies (published and unpublished) examining benefits and harms for each intervention	Yes – page 4

For peer review only

	6b	Explanation for choice of comparators – interviews	
Objectives	7	Specific objectives or hypotheses	Yes – page 6
Trial design	8	Description of trial design including type of trial (eg, parallel group, crossover, factorial, single group), allocation ratio, and framework (eg, superiority, equivalence, noninferiority, exploratory)	Yes – page 6
Methods: Participants, interventions, and outcomes			
Study setting	9	Description of study settings (eg, community clinic, academic hospital) and list of countries where data will be collected. Reference to where list of study sites can be obtained	Yes – page 6
Eligibility criteria	10	Inclusion and exclusion criteria for participants. If applicable, eligibility criteria for study centres and individuals who will perform the interventions (eg, surgeons, psychotherapists)	Yes – page 8 (table 1)
Interventions	11a	Interventions for each group with sufficient detail to allow replication, including how and when they will be administered	Yes – page 9 (one group only)
	11b	Criteria for discontinuing or modifying allocated interventions for a given trial participant (eg, drug dose change in response to harms, participant request, or improving/worsening disease)	Yes – page 9
	11c	Strategies to improve adherence to intervention protocols, and any procedures for monitoring adherence (eg, drug tablet return, laboratory tests)	N/A for this study
	11d	Relevant concomitant care and interventions that are permitted or prohibited during the trial	Yes – page 8 (exclusion criteria)
Outcomes	12	Primary, secondary, and other outcomes, including the specific measurement variable (eg, systolic blood pressure), analysis metric (eg, change from baseline, final value, time to event), method of aggregation (eg, median, proportion), and time point for each outcome. Explanation of the clinical relevance of chosen efficacy and harm outcomes is strongly recommended	Yes – page 11
Participant timeline	13	Time schedule of enrolment, interventions (including any run-ins and	Yes – pages 11-13

		washouts), assessments, and visits for participants. A schematic diagram is highly recommended (see Figure)	
Sample size	14	Estimated number of participants needed to achieve study objectives and how it was determined, including clinical and statistical assumptions supporting any sample size calculations	Yes – page 6
Recruitment	15	Strategies for achieving adequate participant enrolment to reach target sample size	Yes – page 7
**Methods: Assignment of interventions (for controlled trials)**			
Allocation:			
Sequence generation	16a	Method of generating the allocation sequence (eg, computer-generated random numbers), and list of any factors for stratification. To reduce predictability of a random sequence, details of any planned restriction (eg, blocking) should be provided in a separate document that is unavailable to those who enrol participants or assign interventions	N/A
Allocation concealment mechanism	16b	Mechanism of implementing the allocation sequence (eg, central telephone; sequentially numbered, opaque, sealed envelopes), describing any steps to conceal the sequence until interventions are assigned	N/A
Implementation	16c	Who will generate the allocation sequence, who will enrol participants, and who will assign participants to interventions	N/A
Blinding (masking)	17a	Who will be blinded after assignment to interventions (eg, trial participants, care providers, outcome assessors, data analysts), and how	N/A
	17b	If blinded, circumstances under which unblinding is permissible, and procedure for revealing a participant's allocated intervention during the trial	N/A
**Methods: Data collection, management, and analysis**			
Data collection methods	18a	Plans for assessment and collection of outcome, baseline, and other trial data, including any related processes to promote data quality (eg, duplicate measurements, training of assessors) and a description of study instruments	Yes – pages 11-12

For peer review only - http://bmjopen.bmj.com/site/about/guidelines.xhtml

		(eg, questionnaires, laboratory tests) along with their reliability and validity, if known. Reference to where data collection forms can be found, if not in the protocol	
	18b	Plans to promote participant retention and complete follow-up, including list of any outcome data to be collected for participants who discontinue or deviate from intervention protocols	Yes – page 10 (weekly contact)
Data management	19	Plans for data entry, coding, security, and storage, including any related processes to promote data quality (eg, double data entry; range checks for data values). Reference to where details of data management procedures can be found, if not in the protocol	Yes – page 12 and 14
Statistical methods	20a	Statistical methods for analysing primary and secondary outcomes. Reference to where other details of the statistical analysis plan can be found, if not in the protocol	N/A
	20b	Methods for any additional analyses (eg, subgroup and adjusted analyses)	N/A
	20c	Definition of analysis population relating to protocol non-adherence (eg, as randomised analysis), and any statistical methods to handle missing data (eg, multiple imputation)	N/A
**Methods: Monitoring**			
Data monitoring	21a	Composition of data monitoring committee (DMC); summary of its role and reporting structure; statement of whether it is independent from the sponsor and competing interests; and reference to where further details about its charter can be found, if not in the protocol. Alternatively, an explanation of why a DMC is not needed	N/A
	21b	Description of any interim analyses and stopping guidelines, including who will have access to these interim results and make the final decision to terminate the trial	N/A
Harms	22	Plans for collecting, assessing, reporting, and managing solicited and spontaneously reported adverse events	Yes – page 14

		and other unintended effects of trial interventions or trial conduct	
Auditing	23	Frequency and procedures for auditing trial conduct, if any, and whether the process will be independent from investigators and the sponsor	Yes – page 14
Ethics and dissemination			
Research ethics approval	24	Plans for seeking research ethics committee/institutional review board (REC/IRB) approval	Yes – page 15
Protocol amendments	25	Plans for communicating important protocol modifications (eg, changes to eligibility criteria, outcomes, analyses) to relevant parties (eg, investigators, REC/IRBs, trial participants, trial registries, journals, regulators)	Yes – page 15
Consent or assent	26a	Who will obtain informed consent or assent from potential trial participants or authorised surrogates, and how (see Item 32)	Yes – page 9
	26b	Additional consent provisions for collection and use of participant data and biological specimens in ancillary studies, if applicable	N/A

*It is strongly recommended that this checklist be read in conjunction with the SPIRIT 2013 Explanation & Elaboration for important clarification on the items. Amendments to the protocol should be tracked and dated. The SPIRIT checklist is copyrighted by the SPIRIT Group under the Creative Commons "Attribution-NonCommercial-NoDerivs 3.0 Unported" license.

For peer review only

BMJ Open

A Sensory Training System (STS) for use at home by people with Complex Regional Pain Syndrome in England: protocol for a proof-of-concept study

Journal:	*BMJ Open*
Manuscript ID	bmjopen-2022-070920.R2
Article Type:	Protocol
Date Submitted by the Author:	17-Apr-2023
Complete List of Authors:	Coggins, Jessica; University of the West of England, School for Health and Social Wellbeing; Royal United Hospital Bath NHS Trust, CRPS Service<br>Grieve, Sharon; Royal United Hospital Bath NHS Trust, CRPS Service; University of the West of England, School of Health and Social Wellbeing<br>Hart, Darren; Royal United Hospital Bath NHS Trust, Medical Physics & Bioengineering Department<br>Llewellyn, Alison ; University of the West of England Bristol, School of Health and Social Wellbeing; Dorothy House Hospice<br>Palmer, Mark; University of the West of England, Department of Computer Science and Creative Technologies<br>Boichat, Charlotte; The Open University, School of Psychology and Counselling<br>McCabe, Candy; University of the West of England, School of Health and Social Wellbeing; Dorothy House Hospice
**Primary Subject Heading**:	Rehabilitation medicine
Secondary Subject Heading:	Rehabilitation medicine
Keywords:	Electric Stimulation Therapy, PAIN MANAGEMENT, REHABILITATION MEDICINE, Clinical trials < THERAPEUTICS

SCHOLARONE™ Manuscripts

Title page

Title: A Sensory Training System (STS) for use at home by people with Complex Regional Pain Syndrome in England: protocol for a proof-of-concept study

Corresponding author: Jessica Coggins, University of the West of England, Glenside Campus, Blackberry Hill, Bristol, BS16 1DD, Jessica.Coggins@uwe.ac.uk

Author's institutional affiliations:

Jessica Coggins, School of Health and Social Wellbeing, University of the West of England, Blackberry Hill, Bristol, BS16 1DD and CRPS Service, Royal United Hospitals Bath NHS Foundation Trust, Combe Park, Bath, BA1 3NG

Dr Sharon Grieve, CRPS Service, Royal United Hospitals Bath NHS Foundation Trust, Combe Park, Bath, BA1 3NG and School of Health and Social Wellbeing, University of the West of England, Blackberry Hill, Bristol, BS16 1DD

Dr Darren Hart, Medical Physics & Bioengineering Department, Royal United Hospitals Bath NHS Foundation Trust, Combe Park, Bath, BA1 3NG

Dr Alison Llewellyn, School of Health and Social Wellbeing, University of the West of England, Blackberry Hill, Bristol, BS16 1DD and Dorothy House Hospice Care, Winsley, Bradford on Avon, BA15 2LE

Dr Mark Palmer, Department of Computer Science and Creative Technologies, University of the West of England, Coldharbour Lane, Bristol, BS16 1QY

Dr Charlotte Boichat, School of Psychology and Counselling, The Open University, Walton Hall, Milton Keynes, MK7 6AA

Professor Candida McCabe, School of Health and Social Wellbeing, University of the West of England, Blackberry Hill, Bristol, BS16 1DD and Dorothy House Hospice Care, Winsley, Bradford on Avon, BA15 2LE

Word count (excluding title page, abstract, references, acknowledgements, contributions, figures, and tables): 4529

Keywords: Complex Regional Pain Syndrome, Sensory Training System, Home use, Sensory discrimination, Electrical stimulation, Game-based therapy.

For peer review only - http://bmjopen.bmj.com/site/about/guidelines.xhtml

ABSTRACT

Introduction: Complex Regional Pain Syndrome (CRPS) is a disabling and distressing chronic pain condition characterised by a range of sensory, motor, autonomic and trophic symptoms. UK guidelines recommend therapy interventions to help normalise touch perception through self-administered tactile and thermal desensitisation activities. Interventions have been developed, aiming to help individuals broaden their sensory experience, thereby relieving chronic pain. However, therapy-led interventions often experience practical constraints and poor adherence. In response, a sensory training system (STS) device has been designed for unsupervised independent home-use.

Methods: This proof-of-concept study aims to explore whether people with CRPS use the device at home for 30 minutes a day for 30-days. Secondary aims are to determine whether the STS device will change tactile acuity and perceived levels of pain intensity, pain interference, sensitivity, or feelings towards the affected limb.

We will seek to recruit 20 eligible participants. Participants will be asked to measure tactile acuity using a two-point discrimination assessment, complete an online questionnaire before and after use of the device and complete a daily diary. Upon completion of the 30-day use, participants will be invited to take part in a semi-structured interview to explore their experiences of using the device.

Analysis: Pain intensity and pain interference will be scored using the online Assessment Center Scoring Service or using the look-up table in the PROMIS scoring manual. The remaining questionnaire data, including tactile acuity results, and device-use data, including frequency and duration of use, will be analysed using descriptive statistics. Qualitative data will be thematically analysed.

Ethics and dissemination: London-Stanmore Research Ethics Committee provided a favourable opinion on 19th April 2021 (ref 21/LO/0200). The NHS Health Research Authority, UK approved this study on 7th June 2021. Dissemination will include peer reviewed publications, presentations at conferences, social media, and reports to the funder and patient charities.

Trial registration: The trial was registered with ISRCTN registry on 28th May 2021 (ISRCTN89099843).

For peer review only

For peer review only - http://bmjopen.bmj.com/site/about/guidelines.xhtml

Article Summary

Strengths and limitations of this study

- The study team compromises a multidisciplinary team with varying backgrounds.
- Due to the pandemic the study was redesigned to be delivered remotely, broadening the geographic location of participants.
- Public contributors have been involved in all aspects of the study including design and monitoring.
- Medicines and Healthcare products Regulatory Agency approval gained for the clinical investigation of this non-CE marked STS device.
- Limitations of the study arise from its modest sample size and remote conduct which limits personal contact between researchers and participants; excludes individuals with limited or no access to the online technology; excludes those who are unable to identify a family member or friend to complete the two-point discrimination assessment; and also means the fidelity of this two-point discrimination assessment cannot be guaranteed.

ew only

For peer review only - http://bmjopen.bmj.com/site/about/guidelines.xhtml

INTRODUCTION

Background and rationale

Complex Regional Pain Syndrome (CRPS) is a chronic pain condition which usually develops after trauma to a limb but can occur spontaneously. CRPS often only affects one limb but can spread to involve additional limbs [1]. The condition is characterised by continuing pain disproportionate to the injury, in addition to abnormal sensory, vasomotor, sudomotor and trophic symptoms. The presence of these symptoms is used in the diagnosis of CRPS in accordance with the validated Budapest Criteria [2]. One striking feature of CRPS is allodynia; pain due to a normally innocuous stimuli, that is experienced in the affected limb. This can result in increased sensitivity to a range of stimuli, for example, clothing cannot be tolerated over the painful region. The degree of sensitivity may vary between individuals. People with CRPS often carefully protect their limb to prevent any physical contact to the allodynic site [3] and they can develop a strong dislike of the CRPS affected limb [4]. The cause of CRPS is unknown, but evidence suggests there are multiple mechanisms involved in the pathophysiology [5,6].

There is limited evidence to estimate the prevalence of CRPS within the United Kingdom, however, a study from 2007 suggests that the European incidence rate of CRPS is 26.2 per 100,000 person years [7]. CRPS occurs in approximately 4-7% of patients who experience limb fractures, injuries, or surgery [6]. For many CRPS patients, symptoms such as swelling, limb discoloration and temperature changes improve within six to 13 months, however, functional limitations persist for some for more than one year [8]. Physical impairment and severe pain continue two years after initial onset for approximately 15% of patients [9]. These populations experience high levels of disability, and care is primarily focused on reducing pain, preserving, or restoring function, and enabling patients to self-manage their condition [9].

Evidence suggests that CRPS patients experience a loss of quality of life due to reduced physical health, even more so than patients with other chronic pain conditions [10]. Alongside individual consequences, the annual economic consequences of CRPS are high [11]. This Swiss study found average treatment costs were 13 times higher, and the number of working days lost 20 times higher within the first two years after the accident in people with CRPS, compared to those without CRPS.

CRPS is associated with a decreased ability to determine the texture, temperature, and location of a stimulus to the painful area [12]. Clinically, the ability to discriminate sensations can be assessed with several methods including a two-point

For peer review only - http://bmjopen.bmj.com/site/about/guidelines.xhtml

discrimination test. Two probes are applied at the same time to the person's skin, and they are asked to distinguish one or two contact points. The shorter the recorded two-probe distance, the better the sensory discrimination ability. Clinical studies have demonstrated that poor two-point discrimination is correlated with higher intensity of pain experienced and with altered brain representation of the affected body part [13, 14]. Interventions have been developed which aim to improve two-point discrimination ability, and therefore reduce pain, by normalising the brain's representation of the painful area. Effectively these interventions seek to help the individual to broaden their sensory experience, thereby 're-finding' normal sensations and relieving chronic pain [15].

Sensory discrimination training which involves cognitive effort can improve two-point discrimination, therefore, reducing patient-reported pain. This has been evidenced for electrical stimulation in amputee PLP, mechanical stimuli, and touch tasks in CRPS, and touch stimuli in chronic low back pain [15-19]. It has been suggested that sensory discrimination training requiring cognitive effort can also have a positive effect on normalising the brain's representation of the painful area [16]. UK Guidelines for adults with CRPS recommend therapy interventions to help normalise touch perception via self-administered tactile and thermal desensitisation activities [9]. Current sensory discrimination training involves repetitive, manual application of different textiles or stimuli to the 'affected', and an 'unaffected' body site, normally by a therapist or carer [20, 21]. However, therapy is slow to deliver improvements, and adherence is poor with limited evidence for any therapist-led interventions that deliver sustained clinically important differences in the long term [22].

Mindful of the practical constraints of the NHS and poor adherence to sensory discrimination training in practice, a home-based sensory discrimination training intervention has been developed by the research team. It is essential that any device created for this purpose is well-developed, reliable, and suitable for unsupervised independent use to gain the expected benefit. A recent study explored the feasibility and acceptability of a novel home-based sensory perception training game for patients with fibromyalgia [23]. Results from this study suggest that a home-based device with a games element is engaging, satisfying and has high adherence. There are commercially available devices that passively stimulate areas of the body but do not include a training element which actively requires cognitive effort in combination with an electrical stimulus. The training element is suggested to be an essential factor in improving two-point discrimination and pain [17, 24].

Our device consists of a commercially available stimulator (Hasomed, RehaMove3), tablet computer (Microsoft Surface Go tablet device) and wearable arm and leg bands (adapted neoprene sports band, commonly used to hold smartphones) see Figure 1. The bands were adapted for the study to comfortably house an electrode

For peer review only - http://bmjopen.bmj.com/site/about/guidelines.xhtml

array against the participants' skin. The electrode array comprises four colour coded electrodes, with five different array sizes to enable the difficultly of the sensory-discrimination task to be increased. The electrodes are represented on the tablet screen as four colour coded images that match the location of the colour coded electrodes in the array. The participant has the option to play one of two games or use the music player as a relaxation activity.

This current research project builds on 14 years of research and development, throughout which the authors have worked with people with CRPS to design a prototype sensory training system (STS) for use at home to improve tactile acuity [25, 26, 27]. The overall goal is to develop a commercially available, clinically effective STS for independent home use by people who have a persistent limb pain, so they can re-gain normal limb sensation. This protocol describes the clinical testing of the next generation STS, that comprises a customised wearable electrode array and game-based training environment, which provides an easy to use and engaging electrical STS for use in the home. The device has received Medicines & Healthcare products Regulatory Agency (MHRA; ref: CI/2021/0019/GB) approval.

Research aim and objectives

This study aims to collect pilot clinical data on a sample of CRPS patients to determine STS duration of use over a 30-day period, and its potential relationship with two-point discrimination and pain intensity.

The primary objective is to explore whether people with CRPS will use the STS device in their own homes adhering to the treatment plan of a minimum of 30 minutes a day for 30 days (the therapeutic dose). Electrical stimulation of 30 minutes per day for a four-week period has been shown in a PLP population to deliver measurable improvements in pain intensity and changes on cortical body maps towards normalisation [16]. The secondary objectives are:

- To determine whether use of the STS device at the therapeutic dose changes tactile acuity (measured using two-point discrimination).
- To determine whether use of the STS device at the therapeutic dose affects perceived levels of pain intensity, pain interference, sensitivity, or feelings towards the affected limb.
- To establish whether participants have found using the STS device to be acceptable and feasible in terms of setting up the device, attaching and detaching the electrode array by themselves.
- To establish whether participants have found using the STS device to be sufficiently engaging to encourage adherence to the therapeutic dose.

For peer review only - http://bmjopen.bmj.com/site/about/guidelines.xhtml

METHODOLOGY

This protocol is version 5.0 dated 24th February 2022. Recruitment commenced in September 2021 and is due to close in April 2023.

Study design

This is a proof-of-concept study designed to explore the acceptability and useability of a customised STS.

Study setting

In response to the COVID-19 pandemic, this study will be conducted remotely across the United Kingdom. This is a single centre study based at the Royal United Hospitals Bath NHS Foundation Trust (RUH), in collaboration with the University of the West of England (UWE).

Participants and sample size

The study aims to recruit 20 adult participants who meet the inclusion criteria and who are willing to use the STS in their homes for 30 minutes per day for 30 days. This target has been based on prior work by the study team and is a pragmatic sample size based on the scale of funding awarded. Due to the severity of symptoms experienced with CRPS and the common occurrence of 'flares' of symptoms, we nevertheless anticipate a number of withdrawals. All data will be reported, including incomplete data sets and reasons for withdrawal, if provided.

Evidence suggests that the Budapest criteria is the preferred diagnostic tool for adults with CRPS [2, 28] , therefore, adults meeting this criteria will be recruited from the CRPS UK Registry (www.crpsnetworkuk.org) or the national CRPS service at the RUH. The Registry Administrator will email a study invitation letter and the Participant Information Sheet (PIS) to potential participants. If the recruitment target is not met via the CRPS UK Registry, patients of the RUH national CRPS specialist service who have previously consented to be contacted regarding research will be emailed the study invitation letter and the PIS by the researchers. Patients attending the CRPS service will also be provided with the study information by a clinician. Potential participants from the CRPS UK Registry who wish to take part will have their contact details passed to the researcher by the Registry administrator. Potential participants recruited via the national CRPS service will contact the researcher directly by email if they wish to take part. The inclusion and exclusion criteria are detailed in Table 1.

For peer review only - http://bmjopen.bmj.com/site/about/guidelines.xhtml

Table 1. Inclusion and exclusion criteria.

**Inclusion criteria**
• Adults (18+) meeting the Budapest clinical criteria [2] for upper or lower limb CRPS Type I.<br>• An area on their limb where a wearable band with electrode array can be attached above their painful site.<br>• An average pain level in the last 7 days rated as ≥5 at rest on a 0-10 scale.<br>• Access to appropriate technology and willingness to use this technology, that enables full study participation (a smart phone, tablet computer or computer compatible with using the video call function on Microsoft Teams, internet access, an email address, and physical and mental capacity to tolerate use of technology).<br>• A person within their household, carer or a friend to carry out the two-point discrimination measurement.
**Exclusion criteria**
• Diagnosis of any other neurological, motor disorder or major nerve damage (including CRPS Type II).<br>• Any mental health condition which may detrimentally impede study participation, in the judgement of the patient or researcher.<br>• The presence of any other limb pathology or pain on the affected CRPS limb.<br>• Poor skin condition on the area to be stimulated.<br>• Poorly controlled epilepsy.<br>• Receiving intensive CRPS specific multidisciplinary team rehabilitation in an inpatient setting during their participation in the study or within the previous month.<br>• Unable to understand written or verbal English and give informed consent.<br>• Active medical implants such as cardiac pacemakers or other devices.<br>• Exposed orthopaedic metal work in the area of electrical stimulation.<br>• Pregnancy.<br>• Known allergy to acrylates which are present in some device components.<br>• Those living alone who have not formed a 'support bubble' with another household *(applies only if social distancing measures, as recommended by the government, are still in place, as there would be no-one able to take the two-point discrimination measurement).*

Recruitment and consent process

For peer review only - http://bmjopen.bmj.com/site/about/guidelines.xhtml

Recruitment will commence with convenience sampling; however, purposeful sampling may be required to try to achieve heterogeneity within the CRPS sample population in terms of; range of reported pain intensity, participants with upper and with lower limb CRPS, and range of CRPS duration.

Once potential participants have expressed an interest after receiving the invitation and PIS, a telephone call will be organised by the research team (JC; SG) in which they will explain the details of the study, answer any questions, confirm eligibility and participant contact details, and explain how to provide online consent using the online consent form, provided using Qualtrics [29]. All participants will be made aware that they are free to decline participation and that doing so will not affect their usual care. If participants would like to continue, a link to the Qualtrics consent form will be emailed to the participant. The consent form will include a copy of the full PIS. Participants will be able to download a PDF copy of the completed consent form for their own records.

Participants may withdraw from the study at any point if they wish. Upon withdrawal they will be invited to provide a reason if they choose, to help inform future development of the device. It will be made clear in the consent process that any data already collected will be included in the study. Study withdrawal will not affect their continuing care. It is possible that some participants may experience local irritation at the point at which the electrodes are applied to the skin, if this continues beyond an hour after use and causes discomfort, the participant will be advised to withdraw from the study. For minor, earlier indications of skin irritation, the usage and skin advice will be reviewed, with the aim that it is resolved without becoming an issue.

Intervention design

Upon receipt of completed consent, the STS will be posted to the participant using a secure courier service and a video-call will be arranged. During the video-call, the researcher will discuss the two-point discrimination assessment with the participant. Participants will also be guided through how to access two films: a short film about the two-point discrimination assessment, and an STS instructions for use video which will show them how to unpack and set up the device and start the game-based training. Both films will be available to access through a private YouTube link. Participants will also have access to paper instructions for the use of the device and how to conduct the two-point discrimination measurements.

Participants will be asked to use the STS device every day for a minimum of 30 minutes a day. This can be during a single session or divided across multiple sessions over the day as determined by patient preference. To prevent concentration

For peer review only - http://bmjopen.bmj.com/site/about/guidelines.xhtml

fatigue, it will be recommended that participants do not exceed two hours' use in any one day. To provide support to participants, they will each be contacted weekly by a member of the research team, either by email or telephone, dependent on the participant's preference.

The STS device

The STS device (figure 2) compromises a pattern of four electrodes placed close to, but not on, the area of allodynia using a flexible wearable band. This positioning ensures that the intensity of stimulation is tolerable to the patient and will be less likely to evoke or exacerbate their pain. The electrode array is attached to a stimulator which connects to a tablet computer. The participant uses the computer tablet to set the level of stimulation that each electrodes emits, to the point they can feel the sensation, but it is not painful. The participant can set the current between 1-50mA and the frequency at either 10Hz (slow), 25Hz (medium) and 40Hz (fast) depending on their preference. The burst duration of the stimulation will be between 900-925ms dependent upon the frequency setting. It is anticipated that the participants will be able to attach the electrode array themselves and they will be able to set the intensity of the stimulation when they first use the device and adjust this at the start of each session if required.

The tablet computer provides a game-based training environment which aims to be easy to use and engaging for participants. The STS application has a choice of two training games (Blockbuster and Flower Tower) and one relaxation activity (Music Player), see figure 3. Participants can select which training game they wish to use and/or in which order. Each electrode is a different colour and participants are asked to respond by selecting the corresponding colour on the tablet computer. Flower Tower will ask the participant to accurately recognise which electrode is being stimulated. Blockbuster includes five separate levels, participants will be asked to identify which electrode is being stimulated and in more challenging levels, accurately recognise different patterns of stimulation across the array. Difficulty in the tasks is provided by the stimulation of more than one electrode at any one time, stimulating electrodes that are located adjacent to each other, or complexity of the pattern of stimulation. Encouraging feedback messages are given by the tablet computer throughout game play. During the relaxation activity, participants can listen to music and experience the beat of the music as a sensation through the electrodes. The Music Player involves less interaction and therefore will only be accessible after 15 minutes of game play.

Each participant will receive five different sized electrode arrays and will be able to choose which size they wish to use at the start of training after a discussion with the researcher. The choice will be based on personal preference and practicalities of

For peer review only - http://bmjopen.bmj.com/site/about/guidelines.xhtml

anatomy. The smaller the electrode array, the closer the electrodes are to each other and therefore, the more challenging the sensory discrimination task. Participants will be able to progress through from larger to smaller templates if they are scoring well as indicated by the in-play game feedback. If the participant achieves between 50-70% accuracy, the device will suggest that this is an appropriate level and that they are using an appropriately sized electrode array. If participants achieve above 70% on multiple occasions, the feedback message will encourage them to consider trying a smaller electrode or moving to the next level. Participants will be asked to record which electrode size is used within a daily participant diary. Once the participant has used the device for 30 days, the device will be collected and returned by a courier at the study's cost.

Data collection and analysis

Participants will be asked to complete a Time 1 Questionnaire 48 hours prior to the planned video call. The questionnaire will be hosted by Qualtrics and will include date of birth, gender, which limb(s) is/are affected, duration of CRPS and current medications. The questionnaire will also include five assessments:

1. Assessment of pain intensity. A self-report rating of average pain intensity over the past seven days will be given by participants on an 11-point numerical rating scale (0-10) using the Patient Reported Outcomes Measurement Information System (PROMIS®) Numeric Rating Scale v.1.0 -Pain Intensity 1a.
2. Assessment of pain interference. A self-report rating of pain interference over the past seven days will be given by participants on an 11-point numerical rating scale (0-10). This comprises four items in the PROMIS Item Bank v1.1 – Pain Interference – Short Form 4a [30].
3. Assessment of sensitivity. A self-report rating of average sensitivity over the past seven days will be given by participants on an 11-point numerical rating scale (0-10). In clinical practice, this patient population would commonly be asked about the degree of sensitivity they are experiencing in their affected limb. Patients understanding of this would be how tolerant they are to touch and/or temperature changes within that limb. They would be very familiar with this type of question. Our patient research partners were consulted about all study documentation and approved the wording of this question.
4. Assessment of emotional feeling about their affected limb. A self-report rating ranging from strongly positive to strongly negative will be given by participants on an 11-point numerical rating scale (0-10). This is informed by the Bath CRPS Body Perception Disturbance scale [31].

For peer review only - http://bmjopen.bmj.com/site/about/guidelines.xhtml

5. The result of the sensory discrimination assessment will also be entered by the participant on to the Qualtrics survey at Time 1. Based on prior work [14], this assessment measures the ability to discriminate sensation at two points. The smallest distance the participant can correctly identify on three occasions out of two trials, whether they were touched with one or two points, will be the recorded measurement. Upon recruitment to the study, participants will be required to identify a friend or family member to conduct this measurement. The participant and their nominated family member/friend will be guided through this with a short film created by the research team, and by an information booklet. The participant will be positioned comfortably with the area to be tested exposed and their eyes closed. A two-point discrimination threshold test using a plastic two-point aesthesiometer will be carried out close to the area of allodynia. Each tool has five different coloured marks, each 2cms apart, which determine when the aesthesiometer is at 1cm, 3cm, 5cm, 7cm and 9cm (figure 4). The person conducting the measurement, starts at 9cm and reduces this according to whether the participant can accurately discriminate between one and two points against their skin. This use of colour codes was designed to simplify the two-point discrimination task for the friend or family member and to reduce the possibility of measurement errors. Ideally, this assessment would have been conducted by a member of the research team but due to COVID-19 requires changes in the study, this pragmatic solution was created. The individual conducting the assessment is also asked to note the anatomical location of the assessment so this can be used again at Time 2.

At the end of the 30 days device use, the five assessments will be repeated. Participants will be sent another link via email to complete the Time 2 Questionnaire online using Qualtrics. There will be four additional assessment questions which will measure overall perception of change of pain intensity, pain interference, sensitivity, and emotional feelings towards the affected limb. All will be measured on a 7-point Likert scale ranging from 'very much improved' to 'very much worse'. The sensory discrimination test will again be carried out by a family member or friend and entered into the final section of the Qualtrics survey by the participant.

The questionnaire data will be exported from Qualtrics into Microsoft Excel for the purposes of analysis. Associations between use of the STS device, changes in participants' sensory discrimination, perceived pain intensity, pain interference, sensitivity, emotional feelings about their affected limb, and self-report questionnaire data will be investigated. PROMIS interference items will be scored using the online 'Assessment Center Scoring Service' for assessment of fully anonymised data or using

For peer review only - http://bmjopen.bmj.com/site/about/guidelines.xhtml

the raw score look-up table in the PROMIS scoring manual (https://www.healthmeasures.net/). This service is provided under the auspices of the USA National Institute for Health. Results will be conservatively interpreted in light of the small sample size in this proof-of-concept study.

The primary research aim of investigating whether participants will adhere to using the STS device for the therapeutic dose of 30 minutes a day for 30 days will be addressed by examining data captured by the device. The frequency and duration of active game use for each participant will be recorded by the device and the research team will be able to view this data once the device has been mailed back to the research team.

Participants will be provided with a simple paper diary in which to record changes in array size throughout the 30 days. This will provide an option for them to record any comments on their experience.

Within two weeks of each participant completing the 30 days STS device use at home, the researcher will conduct a telephone interview to ask them about their experiences of participating in the study and of using the device. These qualitative data will be recorded with the participant's consent so that it can be transcribed verbatim. The interview schedule will be semi-structured with open-ended questions and will aim to generate qualitative data about the usability and acceptability of the device. Participants will be informed they do not have to answer any questions they do not wish to. Qualitative data from interviews with participants, will be analysed using thematic analysis [32] utilising QSR International NVivo software (2018). The summarised study design can be found in figure 5.

For peer review

Adverse events

Any adverse events (AE) will be reported to the Chief Investigator and documented within the participant's study documentation. If necessary, the AE will be discussed with the research team to decide on the most appropriate action. In the unlikely event of a serious adverse event (SAE) or serious adverse reaction (SAR), the study team will immediately inform the Chief Investigator, sponsor and the MHRA. Any SAE's that are related to the study and unexpected will be reported to the Research Ethics Committee within 15 days of the Chief Investigator becoming aware of the event (as per the Health Research Authority guidance).

Data management

Participant identifiable information, including contact details, will only be seen by the study team. This information will only be used for the purposes of conducting the online surveys and facilitating study contact.

For peer review only - http://bmjopen.bmj.com/site/about/guidelines.xhtml

The e-consent and e-questionnaires will be hosted on the Qualtrics as provided and licensed by the University of the West of England, UK. Data on the tablets, which record the participants' device use, will be transferred from the tablet by the study team. The data will be deleted after each individual's participation in the study is complete. Patient confidentiality will be maintained, and data will be collected and retained in accordance with the Data Protection Act, 2018 [33].

Paper-based data will be kept securely in locked cabinets at the RUH or UWE and only accessible to the research team. All electronic personal data will be stored on RUH/UWE password-protected computer systems and managed by the research team. At the end of the study, these documents will be destroyed within six months.

Anonymised research data in the study database will be stored at UWE or RUH until the study has been analysed and written for publication. All electronic data will be destroyed within 12 months of study end. Only authorised members of the study team will have access to any electronic data. Data may also be examined by regulatory authorities to check that the study is being carried out correctly. Following the creation of the audio recording from the telephone interview, the file will be password protected and uploaded to a UWE Bristol approved and recommended transcription service. Audio recordings from telephone interviews will be password protected. Once transcribed, the audio recording will be deleted. The transcriptions will be stored in the same way as other anonymised research data.

Study monitoring

The Research and Development office at the RUH monitors projects which they sponsor on an annual basis. At UWE, a Research Governance record is audited quarterly. In addition to a UWE ethics application, UWE also require a study risk assessment to be submitted. This is held by UWE.

Patient and public involvement

For peer review only - http://bmjopen.bmj.com/site/about/guidelines.xhtml

Patient and public involvement (PPI) has been an integral part of the project at every stage. It was the patients' desire for a more effective and efficient means of improving sensation in their painful limbs which inspired the original idea and design of the research. Patients have continued to shape the direction of our project, and the design of the device through its different developmental stages. Two focus groups have been held and patients strongly endorsed the importance of this device, emphasising the considerable impact CRPS has on people's lives and the need for new therapeutic approaches. Public Contributors have provided critical feedback to the study design, including recruitment and data capture processes.

Public contributors will be invited to join the Project Management Group (PMG) and will collaborate with the study team to design any patient-related documents. They will also assist in interpretation of findings and dissemination of results arising from the research. Public Contributors will contribute to the preparation of future funding applications as co-designing the device with patients has been central to this work from the outset.

ETHICAL APPROVAL AND DISSEMINATION

This study has been reviewed and given a favourable ethical opinion by London-Stanmore Research Ethics Committee, UK (ref 21/LO/0200) on 19th April 2021 and approved by the NHS Health Research Authority, UK on 7th June 2021. The Medicines and Healthcare Products Regulatory Agency provided a no grounds for objection decision on 4th June 2021. If an amendment to the study protocol is required, the Chief Investigator will ensure that a valid notice of amendment is submitted, and approval obtained.

On completion of the study a report will be prepared which will be submitted to the Research Ethics Committee and the funders, Versus Arthritis (Award Ref:22029). Dissemination will include peer reviewed publications, presentations at national/international conferences, social media, and reports for relevant patient charities (CRPS UK and Burning Nights). The funder will be acknowledged in the publications and presentations. At the end of the study, the results will be available on the CRPS UK Clinical & Research Network website, and a written summary will be available on request.

Study status: Recruitment and data collection is currently underway and will be completed in April 2023. Data analysis will take place from April 2023 until May 2023. The study is schedule to end in June 2023.

Acknowledgments: We are very grateful to Evey Watson and Krzysztof Chwiolkam, Games Technology students at UWE, who worked with MP to design the

For peer review only - http://bmjopen.bmj.com/site/about/guidelines.xhtml

STS application used within this study; and to the CRPS UK registry who will be involved in the recruitment of participants.

Competing interests statement: The authors declare no conflicts of interest.

Sources of funding: This work is supported by Versus Arthritis grant number 22029.

Trial Sponsor: Professor Olena Doran, University of the West of England, Bristol, BS16 1QY, Olena.Doran@uwe.ac.uk.

Role of the trial Sponsor and funder: The sponsor and funder have no role in the study design; collection, management, analysis, and interpretation of data; writing of the report; and the decision to submit the report for publication.

Author contributions: All authors qualify for authorship in accordance with the definition provided by the International Committee of Medical Journal Editors (ICMJE). CM, DH, AL and SG conceptualised and designed the study and were involved with funding acquisition, making substantial contributions. DH and MP developed the software. CB and SG initially drafted the protocol, and all authors were subsequently involved with revising and agreeing the final version. JC wrote the first draft of the manuscript based on the study protocol. All authors approved the final manuscript before submission.

References

1. van Rijn MA, Marinus J, Putter H, *et al*. Spreading of complex regional pain syndrome: Not a random process. *Journal of Neural Transmission* 2011; 118, 1301–1309. DOI 10.1007/s00702-011-0601-1
2. Harden RN, Bruehl S, Perez RS, *et al*. Validation of proposed diagnostic criteria (the "Budapest Criteria") for complex regional pain syndrome. *Pain* 2010; 150: 268-274. doi: 10.1016/j.pain.2010.04.030
3. Drummond PD. Sensory disturbances in Complex Regional Pain Syndrome: clinical observations, autonomic interactions, and possible mechanisms. *Pain Medicine* 2010; 11: 1257-1266. doi: 10.1111/j.1526-4637.2010.00912
4. Lewis JS, Kersten P, McCabe CS, *et al*. Body perception disturbance: a contribution to pain in Complex Regional Pain Syndrome. *Pain* 2007. 133 (1-3), 111-119. doi: 10.1016/j.pain.2007.03.013
5. Marinus J, Moseley GL, Birklein F et al. Clinical features and pathophysiology of complex regional pain syndrome. *Lancet Neurol* 2011;10: 637–48. doi: https://doi.org/10.1016/S1474-4422(11)70106-5
6. Bruehl S. Complex regional pain syndrome. *BMJ* 2015; 351. doi: 10.1136/bmj.h2730

For peer review only - http://bmjopen.bmj.com/site/about/guidelines.xhtml

7. de Mos M, de Bruijn AG, Huygen FJ, Dieleman JP, Stricker BH, Sturkenboom MC. The incidence of complex regional pain syndrome: a population-based study. *Pain.* 2007;129: 12–20. doi: 10.1016/j.pain.2006.09.008.
8. Bean, DJ, Johnson, MH, Kydd RR. The outcome of complex regional pain syndrome type 1: A systematic review. *The Journal of Pain 2014; 15: 677-690.*
9. Goebel A, Barker CH, Turner-Stokes L *et al*. Complex regional pain syndrome in adults: UK guidelines for diagnosis, referral and management in primary and secondary care. London: Royal College of Physicians, 2018.
10. van Velzen GA, Perez RS, van Gestel MA, *et al*. Health-related quality of life in 975 patients with complex regional pain syndrome type 1. PAIN®. 2014; 155:629-34. doi: 10.1016/j.pain.2013.12.017.
11. Scholz-Odermatt SM, Luthi F, Wertli MM, *et al.* Direct Health Care Cost and Work Incapacity Related to Complex Regional Pain Syndrome in Switzerland: A Retrospective Analysis from 2008 to 2015. *Pain Medicine*. 2019; 20: 1559–1569. doi: 10.1093/pm/pnz030
12. Birklein F, Dimova V. Complex regional pain syndrome–up-to-date. *Pain reports* 2017; 2: e624. doi: 10.1097/PR9.0000000000000624
13. Pleger B, Ragert P, Schwenkreis P, *et al.* Patterns of cortical reorganization parallel impaired tactile discrimination and pain intensity in CRPS. *NeuroImage* 2006; 32: 503-510. doi:10.1016/j.neuroimage.2006.03.045
14. Adamczyk WM, Saulicz O, Saulicz E, *et al*. Tactile acuity (dys)function in acute nociceptive low back pain: a double-blind experiment. *Pain 2018*; 159: 427-436. doi:10.1097/j.pain.0000000000001110
15. Bagg MK, Wand BM, Cashin AG, *et al*. Effect of Graded Sensorimotor Retraining on Pain Intensity in Patients With Chronic Low Back Pain: A Randomized Clinical Trial. *JAMA* 2022*;* 328: 430-439. doi:10.1001/jama.2022.9930
16. Flor H, Denke C, Schaefer M, *et al.* Effect of sensory discrimination training on cortical reorganisation and phantom limb pain. *The Lancet* 2001*;* 357: 1763-1764. doi: 10.1016/S0140-6736(00)04890.
17. Moseley GL, Zalucki NM, Wiech K. Tactile discrimination, but not tactile stimulation alone, reduces chronic limb pain. *Pain* 2008; 137:600-608. doi: 10.1016/j.pain.2007.10.021
18. Schmid AC, Schwarz A, Gustin SM, *et al*. Pain reduction due to novel sensory-motor training in Complex Regional Pain Syndrome I–A pilot study. *Scandinavian Journal of Pain* 2019; 15: 30-37. doi: 10.1016/j.sjpain.2016.11.003
19. Trapp W, Weinberger M, Erk S, *et al*. A brief intervention utilising visual feedback reduces pain and enhances tactile acuity in CLBP patients. *Journal of back and musculoskeletal rehabilitation* 2015; 28: 651-660. doi: 10.3233/BMR-140561
20. Lewis J, Coales, K, Hall J, *et al.* Now you see it, now you don't. Sensory re-education in Complex Regional Pain Syndrome. *Hand Therapy* 2011; 16: 29-38. doi: 10.1258/ht.2011.011005

For peer review only - http://bmjopen.bmj.com/site/about/guidelines.xhtml

21. Packham TL, Spicher CJ, MacDermid JC, *et al.* Somatosensory rehabilitation for allodynia in complex regional pain syndrome of the upper limb: A retrospective cohort study. *Journal of Hand Therapy* 2018; 3: 10-19. doi: 10.1016/j.jht.2017.02.007

22. O'Connell NE, Wand BM, Mcauley J, *et al.* Interventions for treating pain and disability in adults with complex regional pain syndrome- an overview of systematic reviews. *Cochrane Database of Systematic Reviews* 2013; 4. doi: 10.1002/14651858.CD009416.pub2

23. Demoulin C, Labory C, Marcon C, *et al*. Feasibility and Acceptability of a Home-Based Sensory Perception Training Game for Patients with Fibromyalgia: A Pilot Study. *Games for Health Journal* 2023; 12. doi: 10.1089/g4h.2022.0079

24. Ryan CG, King R, Robinson V, *et al.* Transcutaneous electrical nerve stimulation using an LTP- like repetitive stimulation protocol for patients with upper limb complex regional pain syndrome: A feasibility study. *Hand Therapy* 2017; *22*: 52-63. doi: 10.1177/1758998316678588

25. Harris ND, Mohan D, Pleydell-Pearce CW, et al. Improved sensory discrimination in healthy volunteers following electrosensory discrimination training. *Clinical Rehabilitation* 2010; 24:276-287. doi: 10.1177/0269215509352055

26. McCabe C, Grieve S, Harris N. Electrical Sensory Discrimination Therapy in Complex Regional Pain Syndrome. *European Journal of Pain Supplements* 2001; 5:136. doi: 10.1016/S1754-3207(11)70465-5

27. Grieve S, Hart D, Harris N, *et al*. Electrical sensory discrimination therapy in Complex Regional Pain Syndrome. Poster presented at: 5th International Congress on Neuropathic Pain; 2015 May 14-17; Nice.

28. Mesaroli G, Hundert A, Birnie KA, *et al.* Screening and diagnostic tools for complex regional pain syndrome: a systematic review. *Pain* 2021; 162: 1295. doi: 10.1097/j.pain.0000000000002146

29. Qualtrics software *Copyright © 2022 Qualtrics. Qualtrics and all other Qualtrics product or service names are registered trademarks or trademarks of Qualtrics*. Provo, UT, USA. https://www.qualtrics.com

30. Amtmann DA, Cook KF, Jensen MP, *et al*. Development of a PROMIS item bank to measure pain interference. Pain 2010; 150:173-182. doi:10.1016/j.pain.2010.04.025

31. Lewis J, McCabe C. Body perception disturbance in Complex Regional Pain Syndrome (CRPS). *Practical Pain Management* 2010*;* 10: 60-66.

32. Braun V, Clarke V. Using thematic analysis in psychology. *Qualitative Research in Psychology 2006*; 3: 77-101. doi:10.1191/1478088706qp063oa

33. Legislation.go.uk. Data Protection Act [online]. 2018. https://www.legislation.gov.uk/ukpga/2018/12/contents/enacted (Accessed 23 November 2022)

For peer review only - http://bmjopen.bmj.com/site/about/guidelines.xhtml

Figures

1. The STS device.
2. The STS device in use by a member of the research team.
3. The STS games and music player. From left to right; Blockbuster Single Point all, Flower Tower and the Music Player.
4. The marked two-point discrimination tool.
5. The study design.

For peer review only

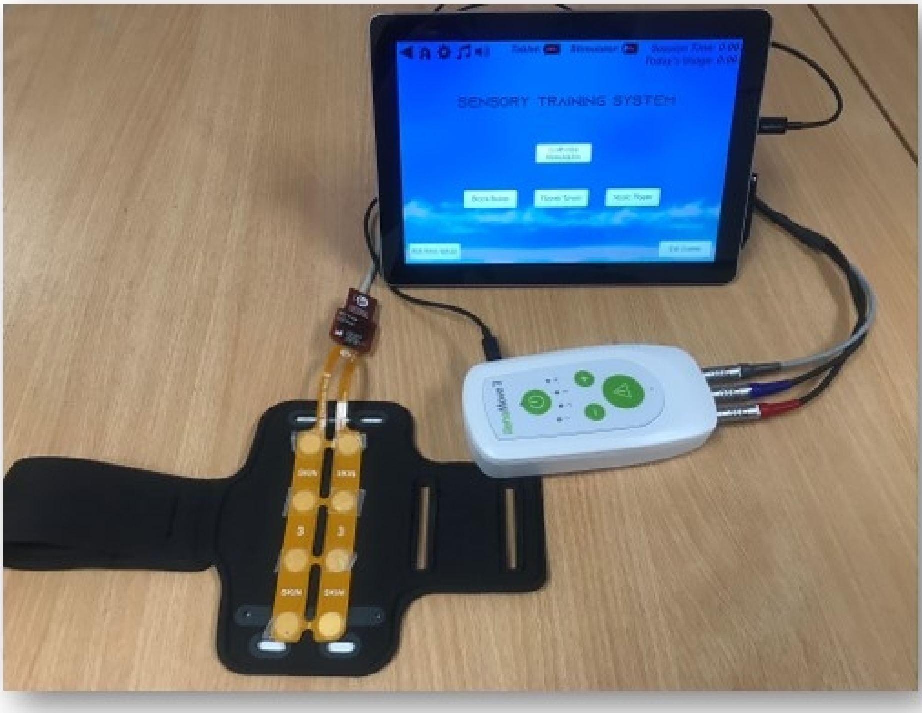

Figure 1. The STS device.

193x149mm (72 x 72 DPI)

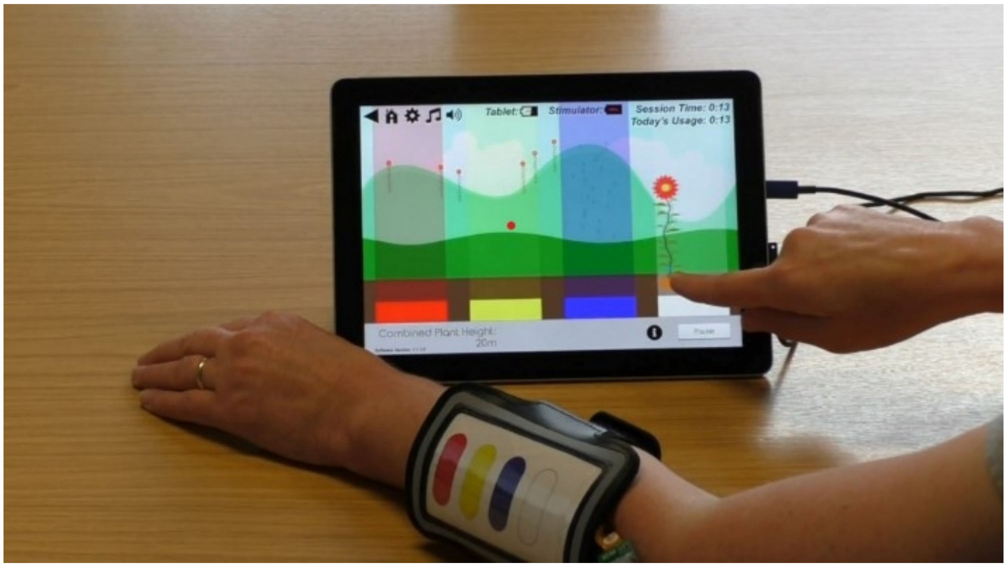

Figure 2. The STS device in use by a member of the research team.

125x69mm (144 x 144 DPI)

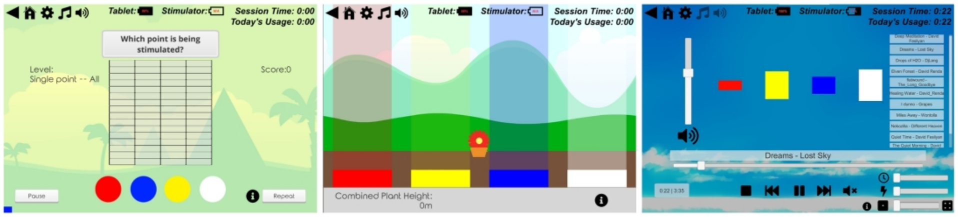

Figure 3. The STS games and music player. From left to right; Blockbuster Single Point all, Flower Tower and the Music Player.

338x190mm (300 x 300 DPI)

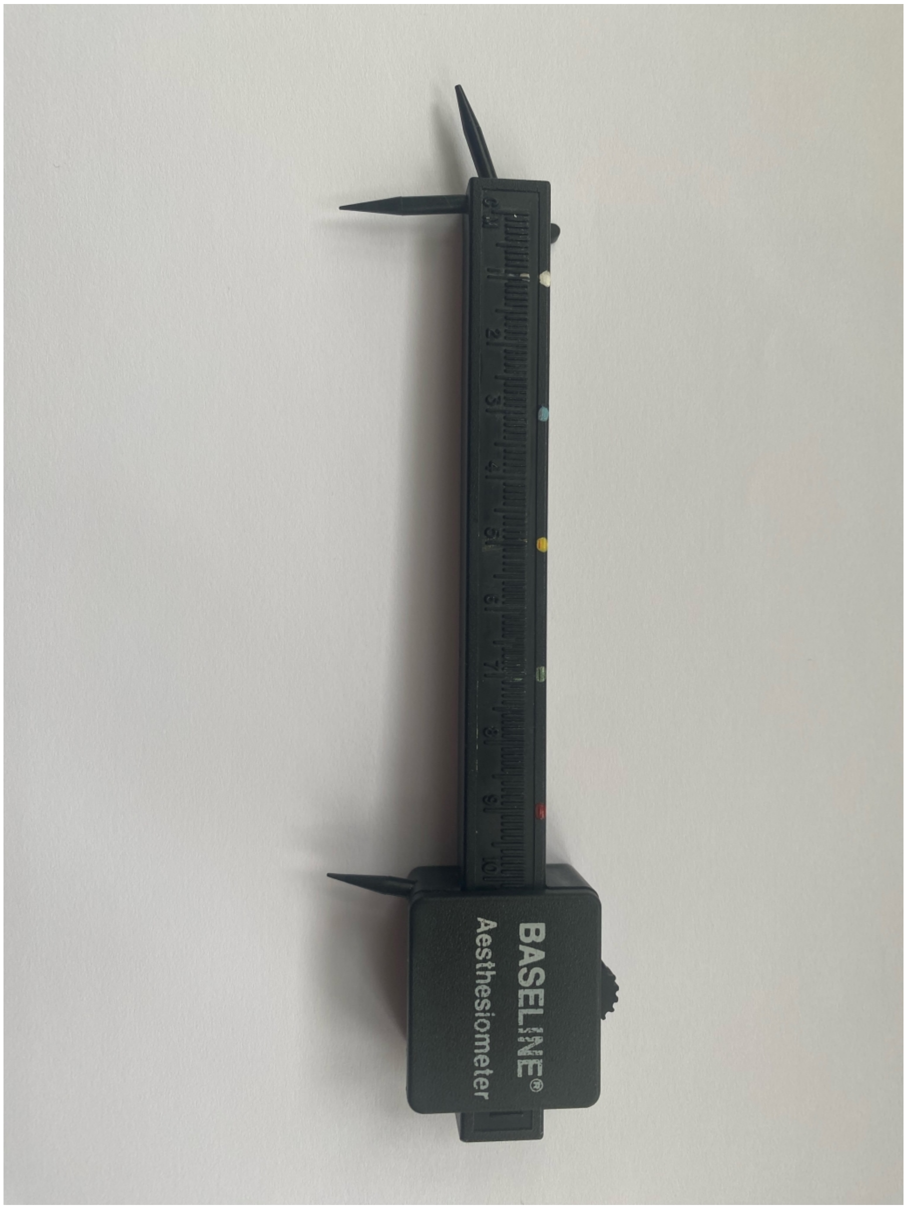

Figure 4. The marked two-point discrimination tool.

1066x1422mm (72 x 72 DPI)

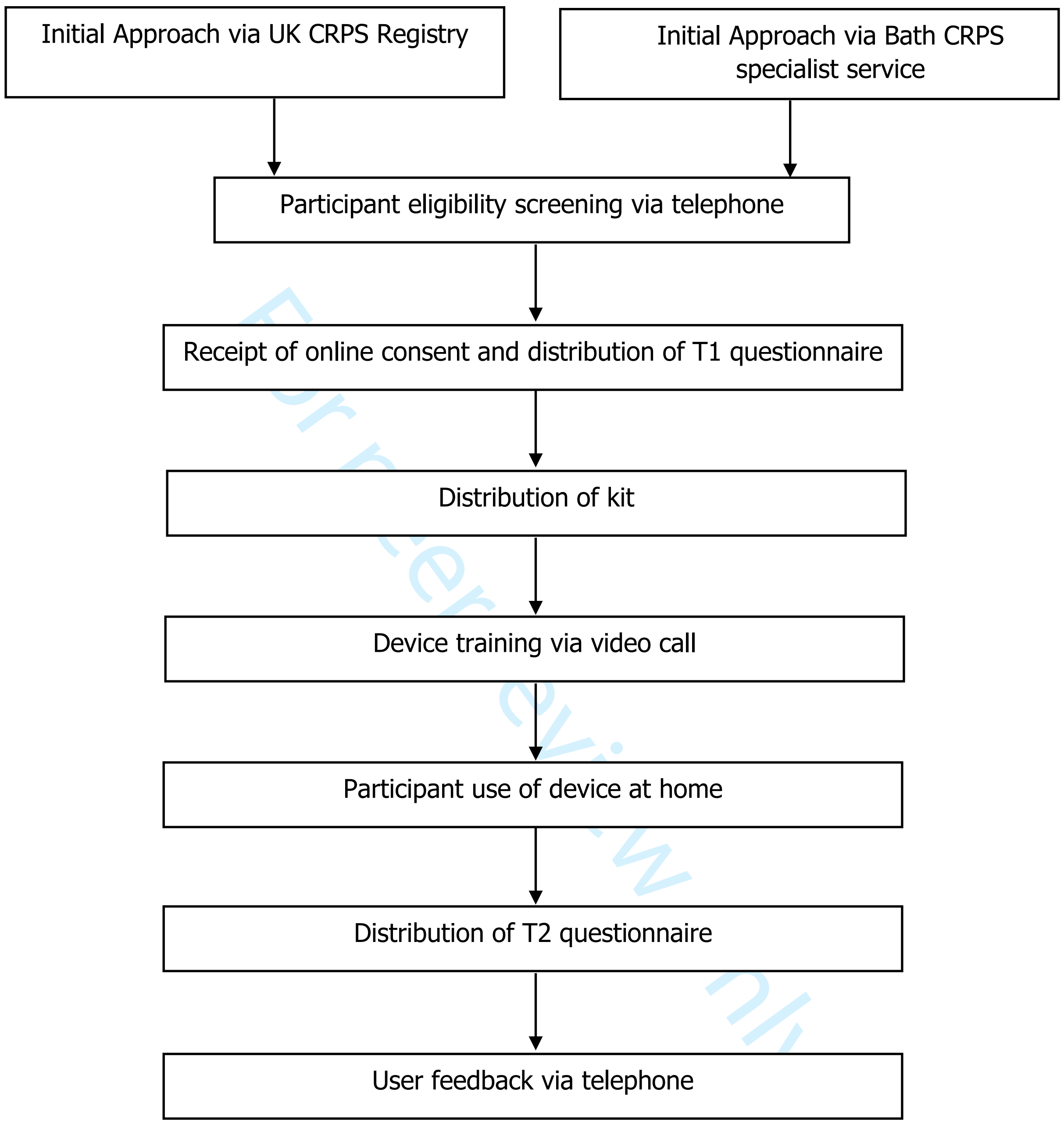

Figure 2. The STS study design.

SPIRIT

STANDARD PROTOCOL ITEMS: RECOMMENDATIONS FOR INTERVENTIONAL TRIALS

SPIRIT 2013 Checklist: Recommended items to address in a clinical trial protocol and related documents*

Section/Item	Item No	Description	Included? Page No
**Administrative information**			
Title	1	Descriptive title identifying the study design, population, interventions, and, if applicable, trial acronym	Yes - page 1
Trial registration	2a	Trial identifier and registry name. If not yet registered, name of intended registry	Yes - page 2
	2b	All items from the World Health Organization Trial Registration Data Set	Yes – throughout
Protocol version	3	Date and version identifier	Yes – page 6
Funding	4	Sources and types of financial, material, and other support	Yes – page 15
Roles and responsibilities	5a	Names, affiliations, and roles of protocol contributors	Yes – page 1
	5b	Name and contact information for the trial sponsor	Yes – page 16
	5c	Role of study sponsor and funders, if any, in study design; collection, management, analysis, and interpretation of data; writing of the report; and the decision to submit the report for publication, including whether they will have ultimate authority over any of these activities	Yes – page 16
	5d	Composition, roles, and responsibilities of the coordinating centre, steering committee, endpoint adjudication committee, data management team, and other individuals or groups overseeing the trial, if applicable (see Item 21a for data monitoring committee)	Yes – page 14
Introduction			
Background and rationale	6a	Description of research question and justification for undertaking the trial, including summary of relevant studies (published and unpublished) examining benefits and harms for each intervention	Yes – page 4

For peer review only

	6b	Explanation for choice of comparators – interviews	
Objectives	7	Specific objectives or hypotheses	Yes – page 6
Trial design	8	Description of trial design including type of trial (eg, parallel group, crossover, factorial, single group), allocation ratio, and framework (eg, superiority, equivalence, noninferiority, exploratory)	Yes – page 6
Methods: Participants, interventions, and outcomes			
Study setting	9	Description of study settings (eg, community clinic, academic hospital) and list of countries where data will be collected. Reference to where list of study sites can be obtained	Yes – page 6
Eligibility criteria	10	Inclusion and exclusion criteria for participants. If applicable, eligibility criteria for study centres and individuals who will perform the interventions (eg, surgeons, psychotherapists)	Yes – page 8 (table 1)
Interventions	11a	Interventions for each group with sufficient detail to allow replication, including how and when they will be administered	Yes – page 9 (one group only)
	11b	Criteria for discontinuing or modifying allocated interventions for a given trial participant (eg, drug dose change in response to harms, participant request, or improving/worsening disease)	Yes – page 9
	11c	Strategies to improve adherence to intervention protocols, and any procedures for monitoring adherence (eg, drug tablet return, laboratory tests)	N/A for this study
	11d	Relevant concomitant care and interventions that are permitted or prohibited during the trial	Yes – page 8 (exclusion criteria)
Outcomes	12	Primary, secondary, and other outcomes, including the specific measurement variable (eg, systolic blood pressure), analysis metric (eg, change from baseline, final value, time to event), method of aggregation (eg, median, proportion), and time point for each outcome. Explanation of the clinical relevance of chosen efficacy and harm outcomes is strongly recommended	Yes – page 11
Participant timeline	13	Time schedule of enrolment, interventions (including any run-ins and	Yes – pages 11-13

		washouts), assessments, and visits for participants. A schematic diagram is highly recommended (see Figure)	
Sample size	14	Estimated number of participants needed to achieve study objectives and how it was determined, including clinical and statistical assumptions supporting any sample size calculations	Yes – page 6
Recruitment	15	Strategies for achieving adequate participant enrolment to reach target sample size	Yes – page 7
**Methods: Assignment of interventions (for controlled trials)**			
Allocation:			
Sequence generation	16a	Method of generating the allocation sequence (eg, computer-generated random numbers), and list of any factors for stratification. To reduce predictability of a random sequence, details of any planned restriction (eg, blocking) should be provided in a separate document that is unavailable to those who enrol participants or assign interventions	N/A
Allocation concealment mechanism	16b	Mechanism of implementing the allocation sequence (eg, central telephone; sequentially numbered, opaque, sealed envelopes), describing any steps to conceal the sequence until interventions are assigned	N/A
Implementation	16c	Who will generate the allocation sequence, who will enrol participants, and who will assign participants to interventions	N/A
Blinding (masking)	17a	Who will be blinded after assignment to interventions (eg, trial participants, care providers, outcome assessors, data analysts), and how	N/A
	17b	If blinded, circumstances under which unblinding is permissible, and procedure for revealing a participant's allocated intervention during the trial	N/A
**Methods: Data collection, management, and analysis**			
Data collection methods	18a	Plans for assessment and collection of outcome, baseline, and other trial data, including any related processes to promote data quality (eg, duplicate measurements, training of assessors) and a description of study instruments	Yes – pages 11-12

For peer review only - http://bmjopen.bmj.com/site/about/guidelines.xhtml

		(eg, questionnaires, laboratory tests) along with their reliability and validity, if known. Reference to where data collection forms can be found, if not in the protocol	
	18b	Plans to promote participant retention and complete follow-up, including list of any outcome data to be collected for participants who discontinue or deviate from intervention protocols	Yes – page 10 (weekly contact)
Data management	19	Plans for data entry, coding, security, and storage, including any related processes to promote data quality (eg, double data entry; range checks for data values). Reference to where details of data management procedures can be found, if not in the protocol	Yes – page 12 and 14
Statistical methods	20a	Statistical methods for analysing primary and secondary outcomes. Reference to where other details of the statistical analysis plan can be found, if not in the protocol	N/A
	20b	Methods for any additional analyses (eg, subgroup and adjusted analyses)	N/A
	20c	Definition of analysis population relating to protocol non-adherence (eg, as randomised analysis), and any statistical methods to handle missing data (eg, multiple imputation)	N/A
**Methods: Monitoring**			
Data monitoring	21a	Composition of data monitoring committee (DMC); summary of its role and reporting structure; statement of whether it is independent from the sponsor and competing interests; and reference to where further details about its charter can be found, if not in the protocol. Alternatively, an explanation of why a DMC is not needed	N/A
	21b	Description of any interim analyses and stopping guidelines, including who will have access to these interim results and make the final decision to terminate the trial	N/A
Harms	22	Plans for collecting, assessing, reporting, and managing solicited and spontaneously reported adverse events	Yes – page 14

		and other unintended effects of trial interventions or trial conduct	
Auditing	23	Frequency and procedures for auditing trial conduct, if any, and whether the process will be independent from investigators and the sponsor	Yes – page 14
Ethics and dissemination			
Research ethics approval	24	Plans for seeking research ethics committee/institutional review board (REC/IRB) approval	Yes – page 15
Protocol amendments	25	Plans for communicating important protocol modifications (eg, changes to eligibility criteria, outcomes, analyses) to relevant parties (eg, investigators, REC/IRBs, trial participants, trial registries, journals, regulators)	Yes – page 15
Consent or assent	26a	Who will obtain informed consent or assent from potential trial participants or authorised surrogates, and how (see Item 32)	Yes – page 9
	26b	Additional consent provisions for collection and use of participant data and biological specimens in ancillary studies, if applicable	N/A

*It is strongly recommended that this checklist be read in conjunction with the SPIRIT 2013 Explanation & Elaboration for important clarification on the items. Amendments to the protocol should be tracked and dated. The SPIRIT checklist is copyrighted by the SPIRIT Group under the Creative Commons "Attribution-NonCommercial-NoDerivs 3.0 Unported" license.

For peer review only

BMJ Open

A Sensory Training System (STS) for use at home by people with Complex Regional Pain Syndrome in England: protocol for a proof-of-concept study

Journal:	*BMJ Open*
Manuscript ID	bmjopen-2022-070920.R3
Article Type:	Protocol
Date Submitted by the Author:	17-May-2023

Complete List of Authors:	Coggins, Jessica; University of the West of England, School for Health and Social Wellbeing; Royal United Hospital Bath NHS Trust, CRPS Service<br>Grieve, Sharon; Royal United Hospital Bath NHS Trust, CRPS Service; University of the West of England, School of Health and Social Wellbeing<br>Hart, Darren; Royal United Hospital Bath NHS Trust, Medical Physics & Bioengineering Department<br>Llewellyn, Alison ; University of the West of England Bristol, School of Health and Social Wellbeing; Dorothy House Hospice<br>Palmer, Mark; University of the West of England, Department of Computer Science and Creative Technologies<br>Boichat, Charlotte; The Open University, School of Psychology and Counselling<br>McCabe, Candy; University of the West of England, School of Health and Social Wellbeing; Dorothy House Hospice

**Primary Subject Heading**:	Rehabilitation medicine
Secondary Subject Heading:	Rehabilitation medicine
Keywords:	Electric Stimulation Therapy, PAIN MANAGEMENT, REHABILITATION MEDICINE, Clinical trials < THERAPEUTICS

SCHOLARONE™
Manuscripts

Title page

Title: A Sensory Training System (STS) for use at home by people with Complex Regional Pain Syndrome in England: protocol for a proof-of-concept study

Corresponding author: Jessica Coggins, University of the West of England, Glenside Campus, Blackberry Hill, Bristol, BS16 1DD, Jessica.Coggins@uwe.ac.uk

Author's institutional affiliations:

Jessica Coggins, School of Health and Social Wellbeing, University of the West of England, Blackberry Hill, Bristol, BS16 1DD and CRPS Service, Royal United Hospitals Bath NHS Foundation Trust, Combe Park, Bath, BA1 3NG

Dr Sharon Grieve, CRPS Service, Royal United Hospitals Bath NHS Foundation Trust, Combe Park, Bath, BA1 3NG and School of Health and Social Wellbeing, University of the West of England, Blackberry Hill, Bristol, BS16 1DD

Dr Darren Hart, Medical Physics & Bioengineering Department, Royal United Hospitals Bath NHS Foundation Trust, Combe Park, Bath, BA1 3NG

Dr Alison Llewellyn, School of Health and Social Wellbeing, University of the West of England, Blackberry Hill, Bristol, BS16 1DD and Dorothy House Hospice Care, Winsley, Bradford on Avon, BA15 2LE

Dr Mark Palmer, Department of Computer Science and Creative Technologies, University of the West of England, Coldharbour Lane, Bristol, BS16 1QY

Dr Charlotte Boichat, School of Psychology and Counselling, The Open University, Walton Hall, Milton Keynes, MK7 6AA

Professor Candida McCabe, School of Health and Social Wellbeing, University of the West of England, Blackberry Hill, Bristol, BS16 1DD and Dorothy House Hospice Care, Winsley, Bradford on Avon, BA15 2LE

Word count (excluding title page, abstract, references, acknowledgements, contributions, figures, and tables): 4593

Keywords: Complex Regional Pain Syndrome, Sensory Training System, Home use, Sensory discrimination, Electrical stimulation, Game-based therapy.

For peer review only - http://bmjopen.bmj.com/site/about/guidelines.xhtml

ABSTRACT

Introduction: Complex Regional Pain Syndrome (CRPS) is a disabling and distressing chronic pain condition characterised by a range of sensory, motor, autonomic and trophic symptoms. UK guidelines recommend therapy interventions to help normalise touch perception through self-administered tactile and thermal desensitisation activities. Interventions have been developed, aiming to help individuals broaden their sensory experience, thereby relieving chronic pain. However, therapy-led interventions often experience practical constraints and poor adherence. In response, a sensory training system (STS) device has been designed for unsupervised independent home-use.

Methods: This proof-of-concept study aims to explore whether people with CRPS use the device at home for 30 minutes a day for 30-days. Secondary aims are to determine whether the STS device will change tactile acuity and perceived levels of pain intensity, pain interference, sensitivity, or feelings towards the affected limb.

We will seek to recruit 20 eligible participants. Participants will be asked to measure tactile acuity using a two-point discrimination assessment, complete an online questionnaire before and after use of the device and complete a daily diary. Upon completion of the 30-day use, participants will be invited to take part in a semi-structured interview to explore their experiences of using the device.

Analysis: Pain intensity and pain interference will be scored using the online Assessment Center Scoring Service or using the look-up table in the PROMIS scoring manual. The remaining questionnaire data, including tactile acuity results, and device-use data, including frequency and duration of use, will be analysed using descriptive statistics. Qualitative data will be thematically analysed.

Ethics and dissemination: London-Stanmore Research Ethics Committee provided a favourable opinion on 19th April 2021 (ref 21/LO/0200). The NHS Health Research Authority, UK approved this study on 7th June 2021. Dissemination will include peer reviewed publications, presentations at conferences, social media, and reports to the funder and patient charities.

Trial registration: The trial was registered with ISRCTN registry on 28th May 2021 (ISRCTN89099843).

For peer review only - http://bmjopen.bmj.com/site/about/guidelines.xhtml

Article Summary

Strengths and limitations of this study

- The study team compromises a multidisciplinary team with varying backgrounds.
- Due to the Covid-19 pandemic the study was redesigned to be delivered remotely, broadening the geographic location of participants.
- Public contributors have been involved in all aspects of the study including design and monitoring.
- Medicines and Healthcare products Regulatory Agency approval gained for the clinical investigation of this non-CE marked STS device.
- Limitations of the study arise from its modest sample size and remote conduct which limits personal contact between researchers and participants; excludes individuals with limited or no access to the online technology; excludes those who are unable to identify a family member or friend to complete the two-point discrimination assessment; and also means the fidelity of this two-point discrimination assessment cannot be guaranteed.

ew only

INTRODUCTION

Background and rationale

Complex Regional Pain Syndrome (CRPS) is a chronic pain condition which usually develops after trauma to a limb but can occur spontaneously. CRPS often only affects one limb but can spread to involve additional limbs [1]. The condition is characterised by continuing pain disproportionate to the injury, in addition to abnormal sensory, vasomotor, sudomotor and trophic symptoms. The presence of these symptoms is used in the diagnosis of CRPS in accordance with the validated Budapest Criteria [2]. One striking feature of CRPS is allodynia; pain due to a normally innocuous stimuli, that is experienced in the affected limb. This can result in increased sensitivity to a range of stimuli, for example, clothing cannot be tolerated over the painful region. The degree of sensitivity may vary between individuals. People with CRPS often carefully protect their limb to prevent any physical contact to the allodynic site [3] and they can develop a strong dislike of the CRPS affected limb [4]. The cause of CRPS is unknown, but evidence suggests there are multiple mechanisms involved in the pathophysiology [5,6].

There is limited evidence to estimate the prevalence of CRPS within the United Kingdom, however, a study from 2007 suggests that the European incidence rate of CRPS is 26.2 per 100,000 person years [7]. CRPS occurs in approximately 4-7% of patients who experience limb fractures, injuries, or surgery [6]. For many CRPS patients, symptoms such as swelling, limb discoloration and temperature changes improve within six to 13 months, however, functional limitations persist for some for more than one year [8]. Physical impairment and severe pain continue two years after initial onset for approximately 15% of patients [9]. These populations experience high levels of disability, and care is primarily focused on reducing pain, preserving, or restoring function, and enabling patients to self-manage their condition [9].

For peer review only

Evidence suggests that CRPS patients experience a loss of quality of life due to reduced physical health, even more so than patients with other chronic pain conditions [10]. Alongside individual consequences, the annual economic consequences of CRPS are high [11]. This Swiss study found average treatment costs were 13 times higher, and the number of working days lost 20 times higher within the first two years after the accident in people with CRPS, compared to those without CRPS.

CRPS is associated with a decreased ability to determine the texture, temperature, and location of a stimulus to the painful area [12]. Clinically, the ability to discriminate sensations can be assessed with several methods including a two-point

For peer review only - http://bmjopen.bmj.com/site/about/guidelines.xhtml

discrimination test. Two probes are applied at the same time to the person's skin, and they are asked to distinguish one or two contact points. The shorter the recorded two-probe distance, the better the sensory discrimination ability. Clinical studies have demonstrated that poor two-point discrimination is correlated with higher intensity of pain experienced and with altered brain representation of the affected body part [13, 14]. Interventions have been developed which aim to improve two-point discrimination ability, and therefore reduce pain, by normalising the brain's representation of the painful area. Effectively these interventions seek to help the individual to broaden their sensory experience, thereby 're-finding' normal sensations and relieving chronic pain [15].

Sensory discrimination training which involves cognitive effort can improve two-point discrimination, therefore, reducing patient-reported pain. This has been evidenced for electrical stimulation in amputee PLP, mechanical stimuli, and touch tasks in CRPS, and touch stimuli in chronic low back pain [15-19]. It has been suggested that sensory discrimination training requiring cognitive effort can also have a positive effect on normalising the brain's representation of the painful area [16]. UK Guidelines for adults with CRPS recommend therapy interventions to help normalise touch perception via self-administered tactile and thermal desensitisation activities [9]. Current sensory discrimination training involves repetitive, manual application of different textiles or stimuli to the 'affected', and an 'unaffected' body site, normally by a therapist or carer [20, 21]. However, therapy is slow to deliver improvements, and adherence is poor with limited evidence for any therapist-led interventions that deliver sustained clinically important differences in the long term [22].

Mindful of the practical constraints of the NHS and poor adherence to sensory discrimination training in practice, a home-based sensory discrimination training intervention has been developed by the research team. It is essential that any device created for this purpose is well-developed, reliable, and suitable for unsupervised independent use to gain the expected benefit. A recent study explored the feasibility and acceptability of a novel home-based sensory perception training game for patients with fibromyalgia [23]. Results from this study suggest that a home-based device with a games element is engaging, satisfying and has high adherence. There are commercially available devices that passively stimulate areas of the body but do not include a training element which actively requires cognitive effort in combination with an electrical stimulus. The training element is suggested to be an essential factor in improving two-point discrimination and pain [17, 24].

Our device consists of a commercially available stimulator (RehaMove3, Hasomed, Germany), tablet computer (Surface Go, Microsoft, United States) and wearable arm and leg bands (adapted neoprene sports band, commonly used to hold smartphones) see Figure 1. The bands were adapted for the study to comfortably

For peer review only - http://bmjopen.bmj.com/site/about/guidelines.xhtml

house an electrode array against the participants' skin. The electrode array comprises four colour coded electrodes, with five different array sizes to enable the difficultly of the sensory-discrimination task to be increased. The electrodes are represented on the tablet screen as four colour coded images that match the location of the colour coded electrodes in the array. The participant has the option to play one of two games or use the music player as a relaxation activity.

This current research project builds on 14 years of research and development, throughout which the authors have worked with people with CRPS to design a prototype sensory training system (STS) for use at home to improve tactile acuity [25, 26, 27]. The overall goal is to develop a commercially available, clinically effective STS for independent home use by people who have a persistent limb pain, so they can re-gain normal limb sensation. This protocol describes the clinical testing of the next generation STS, that comprises a customised wearable electrode array and game-based training environment, which provides an easy to use and engaging electrical STS for use in the home. The device has received Medicines & Healthcare products Regulatory Agency (MHRA; ref: CI/2021/0019/GB) approval.

Research aim and objectives

This study aims to collect pilot clinical data on a sample of CRPS patients to determine STS duration of use over a 30-day period, and its potential relationship with two-point discrimination and pain intensity.

The primary objective is to explore whether people with CRPS will use the STS device in their own homes adhering to the treatment plan of a minimum of 30 minutes a day for 30 days (the therapeutic dose). Electrical stimulation of 30 minutes per day for a four-week period has been shown in a PLP population to deliver measurable improvements in pain intensity and changes on cortical body maps towards normalisation [16]. The secondary objectives are:

- To determine whether use of the STS device at the therapeutic dose changes tactile acuity (measured using two-point discrimination).
- To determine whether use of the STS device at the therapeutic dose affects perceived levels of pain intensity, pain interference, sensitivity, or feelings towards the affected limb.
- To establish whether participants have found using the STS device to be acceptable and feasible in terms of setting up the device, attaching and detaching the electrode array by themselves.
- To establish whether participants have found using the STS device to be sufficiently engaging to encourage adherence to the therapeutic dose.

For peer review only - http://bmjopen.bmj.com/site/about/guidelines.xhtml

METHODOLOGY

This protocol is version 5.0 dated 24th February 2022. Recruitment commenced in September 2021 and is due to close in April 2023.

Study design

This is a proof-of-concept study designed to explore the acceptability and useability of a customised STS.

Study setting

In response to the COVID-19 pandemic, this study will be conducted remotely across the United Kingdom. This is a single centre study based at the Royal United Hospitals Bath NHS Foundation Trust (RUH), in collaboration with the University of the West of England (UWE). A collaboration agreement is in place between the RUH and UWE and any intellectual property is owned by these organisations.

Participants and sample size

The study aims to recruit 20 adult participants who meet the inclusion criteria and who are willing to use the STS in their homes for 30 minutes per day for 30 days. This target has been based on prior work by the study team and is a pragmatic sample size based on the scale of funding awarded. Due to the severity of symptoms experienced with CRPS and the common occurrence of 'flares' of symptoms, we nevertheless anticipate a number of withdrawals. All data will be reported, including incomplete data sets and reasons for withdrawal, if provided.

Evidence suggests that the Budapest criteria is the preferred diagnostic tool for adults with CRPS [2, 28] , therefore, adults meeting this criteria will be recruited from the CRPS UK Registry (www.crpsnetworkuk.org) or the national CRPS service at the RUH. The Registry Administrator will email a study invitation letter and the Participant Information Sheet (PIS) to potential participants. If the recruitment target is not met via the CRPS UK Registry, patients of the RUH national CRPS specialist service who have previously consented to be contacted regarding research will be emailed the study invitation letter and the PIS by the researchers. Patients attending the CRPS service will also be provided with the study information by a clinician. Potential participants from the CRPS UK Registry who wish to take part will have their contact details passed to the researcher by the Registry administrator. Potential participants recruited via the national CRPS service will contact the researcher directly by email if they wish to take part. The inclusion and exclusion criteria are detailed in Table 1.

For peer review only - http://bmjopen.bmj.com/site/about/guidelines.xhtml

Table 1. Inclusion and exclusion criteria.

**Inclusion criteria**
• Adults (18+) meeting the Budapest clinical criteria [2] for upper or lower limb CRPS Type I.<br>• An area on their limb where a wearable band with electrode array can be attached above their painful site.<br>• An average pain level in the last 7 days rated as ≥5 at rest on a 0-10 scale.<br>• Access to appropriate technology and willingness to use this technology, that enables full study participation (a smart phone, tablet computer or computer compatible with using the video call function on Microsoft Teams, internet access, an email address, and physical and mental capacity to tolerate use of technology).<br>• A person within their household, carer or a friend to carry out the two-point discrimination measurement.
**Exclusion criteria**
• Diagnosis of any other neurological, motor disorder or major nerve damage (including CRPS Type II).<br>• Any mental health condition which may detrimentally impede study participation, in the judgement of the patient or researcher.<br>• The presence of any other limb pathology or pain on the affected CRPS limb.<br>• Poor skin condition on the area to be stimulated.<br>• Poorly controlled epilepsy.<br>• Receiving intensive CRPS specific multidisciplinary team rehabilitation in an inpatient setting during their participation in the study or within the previous month.<br>• Unable to understand written or verbal English and give informed consent.<br>• Active medical implants such as cardiac pacemakers or other devices.<br>• Exposed orthopaedic metal work in the area of electrical stimulation.<br>• Pregnancy.<br>• Known allergy to acrylates which are present in some device components.<br>• Those living alone who have not formed a 'support bubble' with another household *(applies only if social distancing measures, as recommended by the government, are still in place, as there would be no-one able to take the two-point discrimination measurement).*

Recruitment and consent process

For peer review only - http://bmjopen.bmj.com/site/about/guidelines.xhtml

Recruitment will commence with convenience sampling; however, purposeful sampling may be required to try to achieve heterogeneity within the CRPS sample population in terms of; range of reported pain intensity, participants with upper and with lower limb CRPS, and range of CRPS duration.

Once potential participants have expressed an interest after receiving the invitation and PIS, a telephone call will be organised by the research team (JC; SG) in which they will explain the details of the study, answer any questions, confirm eligibility and participant contact details, and explain how to provide online consent using the online consent form, provided using Qualtrics [29]. All participants will be made aware that they are free to decline participation and that doing so will not affect their usual care. If participants would like to continue, a link to the Qualtrics consent form will be emailed to the participant. The consent form will include a copy of the full PIS. Participants will be able to download a PDF copy of the completed consent form for their own records.

Participants may withdraw from the study at any point if they wish. Upon withdrawal they will be invited to provide a reason if they choose, to help inform future development of the device. It will be made clear in the consent process that any data already collected will be included in the study. Study withdrawal will not affect their continuing care. It is possible that some participants may experience local irritation at the point at which the electrodes are applied to the skin, if this continues beyond an hour after use and causes discomfort, the participant will be advised to withdraw from the study. For minor, earlier indications of skin irritation, the usage and skin advice will be reviewed, with the aim that it is resolved without becoming an issue.

Intervention design

Upon receipt of completed consent, the STS will be posted to the participant using a secure courier service and a video-call will be arranged. During the video-call, the researcher will discuss the two-point discrimination assessment with the participant. Participants will also be guided through how to access two films: a short film about the two-point discrimination assessment, and an STS instructions for use video which will show them how to unpack and set up the device and start the game-based training. Both films will be available to access through a private YouTube link. Participants will also have access to paper instructions for the use of the device and how to conduct the two-point discrimination measurements.

Participants will be asked to use the STS device every day for a minimum of 30 minutes a day. This can be during a single session or divided across multiple sessions over the day as determined by patient preference. To prevent concentration

For peer review only - http://bmjopen.bmj.com/site/about/guidelines.xhtml

fatigue, it will be recommended that participants do not exceed two hours' use in any one day. To provide support to participants, they will each be contacted weekly by a member of the research team, either by email or telephone, dependent on the participant's preference.

The STS device

The STS device (figure 2) compromises a pattern of four electrodes placed close to, but not on, the area of allodynia using a flexible wearable band. This positioning ensures that the intensity of stimulation is tolerable to the patient and will be less likely to evoke or exacerbate their pain. The electrode array is attached to a stimulator which connects to a tablet computer. The participant uses the computer tablet to set the level of stimulation intensity that each electrodes emits, to the point they can feel the sensation, but it is not painful. Intensity can be controlled within set increments of pulse width between 0% (50μs) and 100% (350μs). The participant can set the current between 1-50mA and the frequency at either 10Hz (slow), 25Hz (medium) and 40Hz (fast) depending on their preference. The burst duration of the spatial stimulation will be between 900-925ms dependent upon the frequency setting. It is anticipated that the participants will be able to attach the electrode array themselves and they will be able to set the intensity of the stimulation when they first use the device and adjust this at the start of each session if required.

The tablet computer provides a game-based training environment which aims to be easy to use and engaging for participants. The STS application has a choice of two training games (Blockbuster and Flower Tower) and one relaxation activity (Music Player), see figure 3. Participants can select which training game they wish to use and/or in which order. Each electrode is a different colour and participants are asked to respond by selecting the corresponding colour on the tablet computer. Flower Tower will ask the participant to accurately recognise which electrode is being stimulated. Blockbuster includes five separate levels, participants will be asked to identify which electrode is being stimulated and in more challenging levels, accurately recognise different patterns of stimulation across the array. Participants can repeat the stimulation burst if they wish. Difficulty in the tasks is provided by the stimulation of more than one electrode at any one time, stimulating electrodes that are located adjacent to each other, or complexity of the pattern of stimulation. Encouraging feedback messages are given by the tablet computer throughout game play. During the relaxation activity, participants can listen to music and experience the beat of the music as a sensation through the electrodes. The Music Player involves less interaction and therefore will only be accessible after 15 minutes of game play.

For peer review only - http://bmjopen.bmj.com/site/about/guidelines.xhtml

Each participant will receive five different sized electrode arrays and will be able to choose which size they wish to use at the start of training after a discussion with the researcher. The choice will be based on personal preference and practicalities of anatomy. The smaller the electrode array, the closer the electrodes are to each other and therefore, the more challenging the sensory discrimination task. Participants will be able to progress through from larger to smaller templates if they are scoring well as indicated by the in-play game feedback. If the participant achieves between 50-70% accuracy, the device will suggest that this is an appropriate level and that they are using an appropriately sized electrode array. If participants achieve above 70% on multiple occasions, the feedback message will encourage them to consider trying a smaller electrode or moving to the next level. Participants will be asked to record which electrode size is used within a daily participant diary. Once the participant has used the device for 30 days, the device will be collected and returned by a courier at the study's cost. The training protocols are proprietary and devised by UWE and the RUH.

Data collection and analysis

Participants will be asked to complete a Time 1 Questionnaire 48 hours prior to the planned video call. The questionnaire will be hosted by Qualtrics and will include date of birth, gender, which limb(s) is/are affected, duration of CRPS and current medications. The questionnaire will also include five assessments:

1. Assessment of pain intensity. A self-report rating of average pain intensity over the past seven days will be given by participants on an 11-point numerical rating scale (0-10) using the Patient Reported Outcomes Measurement Information System (PROMIS®) Numeric Rating Scale v.1.0 -Pain Intensity 1a.
2. Assessment of pain interference. A self-report rating of pain interference over the past seven days will be given by participants on an 11-point numerical rating scale (0-10). This comprises four items in the PROMIS Item Bank v1.1 – Pain Interference – Short Form 4a [30].
3. Assessment of sensitivity. A self-report rating of average sensitivity over the past seven days will be given by participants on an 11-point numerical rating scale (0-10). In clinical practice, this patient population would commonly be asked about the degree of sensitivity they are experiencing in their affected limb. Patients understanding of this would be how tolerant they are to touch and/or temperature changes within that limb. They would be very familiar with this type of question. Our patient research partners were consulted about all study documentation and approved the wording of this question.

For peer review only - http://bmjopen.bmj.com/site/about/guidelines.xhtml

4. Assessment of emotional feeling about their affected limb. A self-report rating ranging from strongly positive to strongly negative will be given by participants on an 11-point numerical rating scale (0-10). This is informed by the Bath CRPS Body Perception Disturbance scale [31].

5. The result of the sensory discrimination assessment will also be entered by the participant on to the Qualtrics survey at Time 1. Based on prior work [14], this assessment measures the ability to discriminate sensation at two points. The smallest distance the participant can correctly identify on three occasions out of two trials, whether they were touched with one or two points, will be the recorded measurement. Upon recruitment to the study, participants will be required to identify a friend or family member to conduct this measurement. The participant and their nominated family member/friend will be guided through this with a short film created by the research team, and by an information booklet. The participant will be positioned comfortably with the area to be tested exposed and their eyes closed. A two-point discrimination threshold test using a plastic two-point aesthesiometer will be carried out close to the area of allodynia. Each tool has five different coloured marks, each 2cms apart, which determine when the aesthesiometer is at 1cm, 3cm, 5cm, 7cm and 9cm (figure 4). The person conducting the measurement, starts at 9cm and reduces this according to whether the participant can accurately discriminate between one and two points against their skin. This use of colour codes was designed to simplify the two-point discrimination task for the friend or family member and to reduce the possibility of measurement errors. Ideally, this assessment would have been conducted by a member of the research team but due to COVID-19 requires changes in the study, this pragmatic solution was created. The individual conducting the assessment is also asked to note the anatomical location of the assessment so this can be used again at Time 2.

At the end of the 30 days device use, the five assessments will be repeated. Participants will be sent another link via email to complete the Time 2 Questionnaire online using Qualtrics. There will be four additional assessment questions which will measure overall perception of change of pain intensity, pain interference, sensitivity, and emotional feelings towards the affected limb. All will be measured on a 7-point Likert scale ranging from 'very much improved' to 'very much worse'. The sensory discrimination test will again be carried out by a family member or friend and entered into the final section of the Qualtrics survey by the participant.

The questionnaire data will be exported from Qualtrics into Microsoft Excel for the purposes of analysis. Associations between use of the STS device, changes in

For peer review only - http://bmjopen.bmj.com/site/about/guidelines.xhtml

participants' sensory discrimination, perceived pain intensity, pain interference, sensitivity, emotional feelings about their affected limb, and self-report questionnaire data will be investigated. PROMIS interference items will be scored using the online 'Assessment Center Scoring Service' for assessment of fully anonymised data or using the raw score look-up table in the PROMIS scoring manual (https://www.healthmeasures.net/). This service is provided under the auspices of the USA National Institute for Health. Results will be conservatively interpreted in light of the small sample size in this proof-of-concept study.

The primary research aim of investigating whether participants will adhere to using the STS device for the therapeutic dose of 30 minutes a day for 30 days will be addressed by examining data captured by the device. The frequency and duration of active game use for each participant will be recorded by the device and the research team will be able to view this data once the device has been mailed back to the research team.

Participants will be provided with a simple paper diary in which to record changes in array size throughout the 30 days. This will provide an option for them to record any comments on their experience.

Within two weeks of each participant completing the 30 days STS device use at home, the researcher will conduct a telephone interview to ask them about their experiences of participating in the study and of using the device. These qualitative data will be recorded with the participant's consent so that it can be transcribed verbatim. The interview schedule will be semi-structured with open-ended questions and will aim to generate qualitative data about the usability and acceptability of the device. Participants will be informed they do not have to answer any questions they do not wish to. Qualitative data from interviews with participants, will be analysed using thematic analysis [32] utilising QSR International NVivo software (2018). The summarised study design can be found in figure 5.

Adverse events

Any adverse events (AE) will be reported to the Chief Investigator and documented within the participant's study documentation. If necessary, the AE will be discussed with the research team to decide on the most appropriate action. In the unlikely event of a serious adverse event (SAE) or serious adverse reaction (SAR), the study team will immediately inform the Chief Investigator, sponsor and the MHRA. Any SAE's that are related to the study and unexpected will be reported to the Research Ethics Committee within 15 days of the Chief Investigator becoming aware of the event (as per the Health Research Authority guidance).

Data management

For peer review only - http://bmjopen.bmj.com/site/about/guidelines.xhtml

Participant identifiable information, including contact details, will only be seen by the study team. This information will only be used for the purposes of conducting the online surveys and facilitating study contact.

The e-consent and e-questionnaires will be hosted on the Qualtrics as provided and licensed by the University of the West of England, UK. Data on the tablets, which record the participants' device use, will be transferred from the tablet by the study team. The data will be deleted after each individual's participation in the study is complete. Patient confidentiality will be maintained, and data will be collected and retained in accordance with the Data Protection Act, 2018 [33].

Paper-based data will be kept securely in locked cabinets at the RUH or UWE and only accessible to the research team. All electronic personal data will be stored on RUH/UWE password-protected computer systems and managed by the research team. At the end of the study, these documents will be destroyed within six months.

Anonymised research data in the study database will be stored at UWE or RUH until the study has been analysed and written for publication. All electronic data will be destroyed within 12 months of study end. Only authorised members of the study team will have access to any electronic data. Data may also be examined by regulatory authorities to check that the study is being carried out correctly. Following the creation of the audio recording from the telephone interview, the file will be password protected and uploaded to a UWE Bristol approved and recommended transcription service. Audio recordings from telephone interviews will be password protected. Once transcribed, the audio recording will be deleted. The transcriptions will be stored in the same way as other anonymised research data.

Study monitoring

The Research and Development office at the RUH monitors projects which they sponsor on an annual basis. At UWE, a Research Governance record is audited quarterly. In addition to a UWE ethics application, UWE also require a study risk assessment to be submitted. This is held by UWE.

Patient and public involvement

Patient and public involvement (PPI) has been an integral part of the project at every stage. It was the patients' desire for a more effective and efficient means of improving sensation in their painful limbs which inspired the original idea and design of the research. Patients have continued to shape the direction of our project, and the design of the device through its different developmental stages. Two focus groups have been held and patients strongly endorsed the importance of this device,

For peer review only - http://bmjopen.bmj.com/site/about/guidelines.xhtml

emphasising the considerable impact CRPS has on people's lives and the need for new therapeutic approaches. Public Contributors have provided critical feedback to the study design, including recruitment and data capture processes.

Public contributors will be invited to join the Project Management Group (PMG) and will collaborate with the study team to design any patient-related documents. They will also assist in interpretation of findings and dissemination of results arising from the research. Public Contributors will contribute to the preparation of future funding applications as co-designing the device with patients has been central to this work from the outset.

ETHICAL APPROVAL AND DISSEMINATION

This study has been reviewed and given a favourable ethical opinion by London-Stanmore Research Ethics Committee, UK (ref 21/LO/0200) on 19th April 2021 and approved by the NHS Health Research Authority, UK on 7th June 2021. The Medicines and Healthcare Products Regulatory Agency provided a no grounds for objection decision on 4th June 2021. If an amendment to the study protocol is required, the Chief Investigator will ensure that a valid notice of amendment is submitted, and approval obtained.

On completion of the study a report will be prepared which will be submitted to the Research Ethics Committee and the funders, Versus Arthritis (Award Ref:22029). Dissemination will include peer reviewed publications, presentations at national/international conferences, social media, and reports for relevant patient charities (CRPS UK and Burning Nights). The funder will be acknowledged in the publications and presentations. At the end of the study, the results will be available on the CRPS UK Clinical & Research Network website, and a written summary will be available on request.

Study status: Recruitment and data collection is currently underway and will be completed in April 2023. Data analysis will take place from April 2023 until May 2023. The study is schedule to end in June 2023.

Acknowledgments: We are very grateful to Evey Watson and Krzysztof Chwiolkam, Games Technology students at UWE, who worked with MP to design the STS application used within this study; and to the CRPS UK registry who will be involved in the recruitment of participants.

Competing interests statement: The authors declare no conflicts of interest.

Sources of funding: This work is supported by Versus Arthritis grant number 22029.

For peer review only - http://bmjopen.bmj.com/site/about/guidelines.xhtml

Trial Sponsor: Professor Olena Doran, University of the West of England, Bristol, BS16 1QY, Olena.Doran@uwe.ac.uk.

Role of the trial Sponsor and funder: The sponsor and funder have no role in the study design; collection, management, analysis, and interpretation of data; writing of the report; and the decision to submit the report for publication.

Author contributions: All authors qualify for authorship in accordance with the definition provided by the International Committee of Medical Journal Editors (ICMJE). CM, DH, AL and SG conceptualised and designed the study and were involved with funding acquisition, making substantial contributions. DH and MP developed the software. CB and SG initially drafted the protocol, and all authors were subsequently involved with revising and agreeing the final version. JC wrote the first draft of the manuscript based on the study protocol. All authors approved the final manuscript before submission.

References

1. van Rijn MA, Marinus J, Putter H, *et al*. Spreading of complex regional pain syndrome: Not a random process. *Journal of Neural Transmission* 2011; 118, 1301–1309. DOI 10.1007/s00702-011-0601-1
2. Harden RN, Bruehl S, Perez RS, *et al*. Validation of proposed diagnostic criteria (the "Budapest Criteria") for complex regional pain syndrome. *Pain* 2010; 150: 268-274. doi: 10.1016/j.pain.2010.04.030
3. Drummond PD. Sensory disturbances in Complex Regional Pain Syndrome: clinical observations, autonomic interactions, and possible mechanisms. *Pain Medicine* 2010; 11: 1257-1266. doi: 10.1111/j.1526-4637.2010.00912
4. Lewis JS, Kersten P, McCabe CS, *et al*. Body perception disturbance: a contribution to pain in Complex Regional Pain Syndrome. *Pain* 2007. 133 (1-3), 111-119. doi: 10.1016/j.pain.2007.03.013
5. Marinus J, Moseley GL, Birklein F et al. Clinical features and pathophysiology of complex regional pain syndrome. *Lancet Neurol* 2011;10: 637–48. doi: https://doi.org/10.1016/S1474-4422(11)70106-5
6. Bruehl S. Complex regional pain syndrome. *BMJ* 2015; 351. doi: 10.1136/bmj.h2730
7. de Mos M, de Bruijn AG, Huygen FJ, Dieleman JP, Stricker BH, Sturkenboom MC. The incidence of complex regional pain syndrome: a population-based study. *Pain.* 2007;129: 12–20. doi: 10.1016/j.pain.2006.09.008.
8. Bean, DJ, Johnson, MH, Kydd RR. The outcome of complex regional pain syndrome type 1: A systematic review. *The Journal of Pain 2014; 15: 677-690.*

For peer review only - http://bmjopen.bmj.com/site/about/guidelines.xhtml

9. Goebel A, Barker CH, Turner-Stokes L *et al*. Complex regional pain syndrome in adults: UK guidelines for diagnosis, referral and management in primary and secondary care. London: Royal College of Physicians, 2018.
10. van Velzen GA, Perez RS, van Gestel MA, *et al*. Health-related quality of life in 975 patients with complex regional pain syndrome type 1. PAIN®. 2014; 155:629-34. doi: 10.1016/j.pain.2013.12.017.
11. Scholz-Odermatt SM, Luthi F, Wertli MM, *et al*. Direct Health Care Cost and Work Incapacity Related to Complex Regional Pain Syndrome in Switzerland: A Retrospective Analysis from 2008 to 2015. *Pain Medicine*. 2019; 20: 1559–1569. doi: 10.1093/pm/pnz030
12. Birklein F, Dimova V. Complex regional pain syndrome–up-to-date. *Pain reports* 2017; 2: e624. doi: 10.1097/PR9.0000000000000624
13. Pleger B, Ragert P, Schwenkreis P, *et al*. Patterns of cortical reorganization parallel impaired tactile discrimination and pain intensity in CRPS. *NeuroImage* 2006; 32: 503-510. doi:10.1016/j.neuroimage.2006.03.045
14. Adamczyk WM, Saulicz O, Saulicz E, *et al*. Tactile acuity (dys)function in acute nociceptive low back pain: a double-blind experiment. *Pain 2018*; 159: 427-436. doi:10.1097/j.pain.0000000000001110
15. Bagg MK, Wand BM, Cashin AG, *et al*. Effect of Graded Sensorimotor Retraining on Pain Intensity in Patients With Chronic Low Back Pain: A Randomized Clinical Trial. *JAMA* 2022*;* 328: 430-439. doi:10.1001/jama.2022.9930
16. Flor H, Denke C, Schaefer M, *et al*. Effect of sensory discrimination training on cortical reorganisation and phantom limb pain. *The Lancet* 2001*;* 357: 1763-1764. doi: 10.1016/S0140-6736(00)04890.
17. Moseley GL, Zalucki NM, Wiech K. Tactile discrimination, but not tactile stimulation alone, reduces chronic limb pain. *Pain* 2008; 137:600-608. doi: 10.1016/j.pain.2007.10.021
18. Schmid AC, Schwarz A, Gustin SM, *et al*. Pain reduction due to novel sensory-motor training in Complex Regional Pain Syndrome I–A pilot study. *Scandinavian Journal of Pain* 2019; 15: 30-37. doi: 10.1016/j.sjpain.2016.11.003
19. Trapp W, Weinberger M, Erk S, *et al*. A brief intervention utilising visual feedback reduces pain and enhances tactile acuity in CLBP patients. *Journal of back and musculoskeletal rehabilitation* 2015; 28: 651-660. doi: 10.3233/BMR-140561
20. Lewis J, Coales, K, Hall J, *et al*. Now you see it, now you don't. Sensory re-education in Complex Regional Pain Syndrome. *Hand Therapy* 2011; 16: 29-38. doi: 10.1258/ht.2011.011005
21. Packham TL, Spicher CJ, MacDermid JC, *et al*. Somatosensory rehabilitation for allodynia in complex regional pain syndrome of the upper limb: A retrospective cohort study. *Journal of Hand Therapy* 2018; 3: 10-19. doi: 10.1016/j.jht.2017.02.007
22. O'Connell NE, Wand BM, Mcauley J, *et al*. Interventions for treating pain and disability in adults with complex regional pain syndrome- an overview of

For peer review only - http://bmjopen.bmj.com/site/about/guidelines.xhtml

systematic reviews. *Cochrane Database of Systematic Reviews* 2013; 4. doi: 10.1002/14651858.CD009416.pub2

23. Demoulin C, Labory C, Marcon C, *et al*. Feasibility and Acceptability of a Home-Based Sensory Perception Training Game for Patients with Fibromyalgia: A Pilot Study. *Games for Health Journal* 2023; 12. doi: 10.1089/g4h.2022.0079
24. Ryan CG, King R, Robinson V, *et al.* Transcutaneous electrical nerve stimulation using an LTP- like repetitive stimulation protocol for patients with upper limb complex regional pain syndrome: A feasibility study. *Hand Therapy* 2017; *22*: 52-63. doi: 10.1177/1758998316678588
25. Harris ND, Mohan D, Pleydell-Pearce CW, et al. Improved sensory discrimination in healthy volunteers following electrosensory discrimination training. *Clinical Rehabilitation* 2010; 24:276-287. doi: 10.1177/0269215509352055
26. McCabe C, Grieve S, Harris N. Electrical Sensory Discrimination Therapy in Complex Regional Pain Syndrome. *European Journal of Pain Supplements* 2001; 5:136. doi: 10.1016/S1754-3207(11)70465-5
27. Grieve S, Hart D, Harris N, *et al*. Electrical sensory discrimination therapy in Complex Regional Pain Syndrome. Poster presented at: 5th International Congress on Neuropathic Pain; 2015 May 14-17; Nice.
28. Mesaroli G, Hundert A, Birnie KA, *et al.* Screening and diagnostic tools for complex regional pain syndrome: a systematic review. *Pain* 2021; 162: 1295. doi: 10.1097/j.pain.0000000000002146
29. Qualtrics software *Copyright © 2022 Qualtrics. Qualtrics and all other Qualtrics product or service names are registered trademarks or trademarks of Qualtrics*. Provo, UT, USA. https://www.qualtrics.com
30. Amtmann DA, Cook KF, Jensen MP, *et al*. Development of a PROMIS item bank to measure pain interference. Pain 2010; 150:173-182. doi:10.1016/j.pain.2010.04.025
31. Lewis J, McCabe C. Body perception disturbance in Complex Regional Pain Syndrome (CRPS). *Practical Pain Management* 2010*;* 10: 60-66.
32. Braun V, Clarke V. Using thematic analysis in psychology. *Qualitative Research in Psychology 2006*; 3: 77-101. doi:10.1191/1478088706qp063oa
33. Legislation.go.uk. Data Protection Act [online]. 2018. https://www.legislation.gov.uk/ukpga/2018/12/contents/enacted (Accessed 23 November 2022)

Figures

1. The STS device.
2. The STS device in use by a member of the research team.
3. The STS games and music player. From left to right; Blockbuster Single Point all, Flower Tower and the Music Player.
4. The marked two-point discrimination tool.

For peer review only - http://bmjopen.bmj.com/site/about/guidelines.xhtml

5. The study design.

For peer review only

For peer review only - http://bmjopen.bmj.com/site/about/guidelines.xhtml

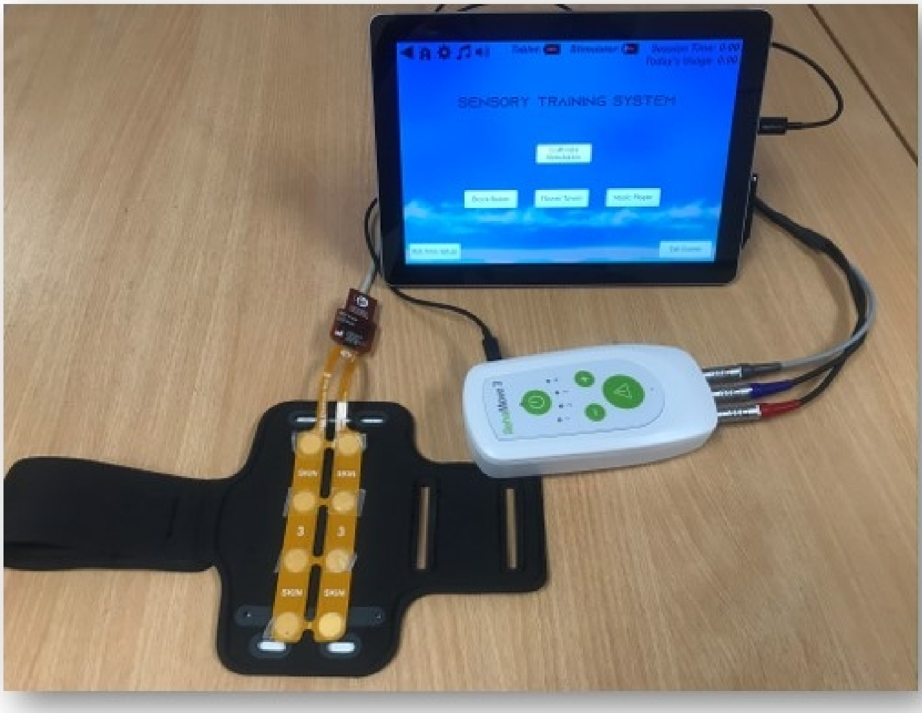

Figure 1. The STS device.

193x149mm (72 x 72 DPI)

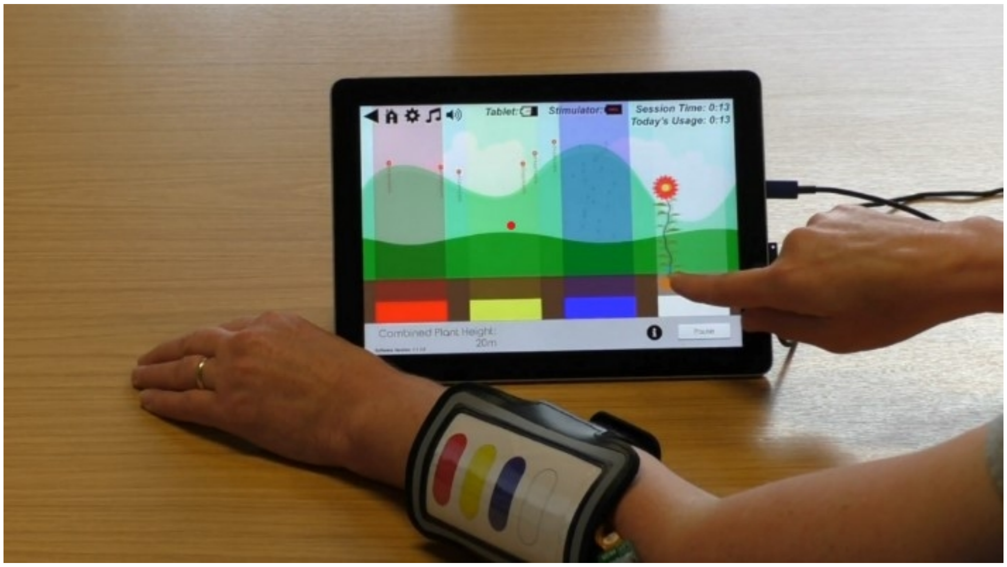

Figure 2. The STS device in use by a member of the research team.

125x69mm (144 x 144 DPI)

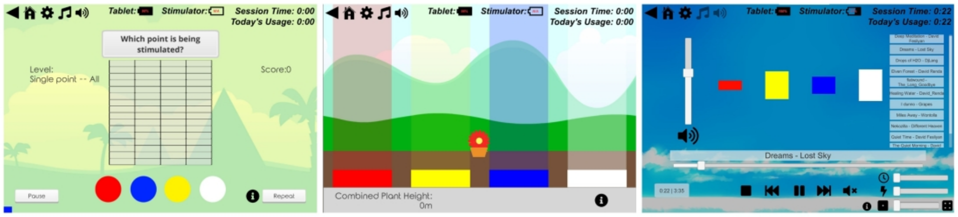

Figure 3. The STS games and music player. From left to right; Blockbuster Single Point all, Flower Tower and the Music Player.

338x190mm (300 x 300 DPI)

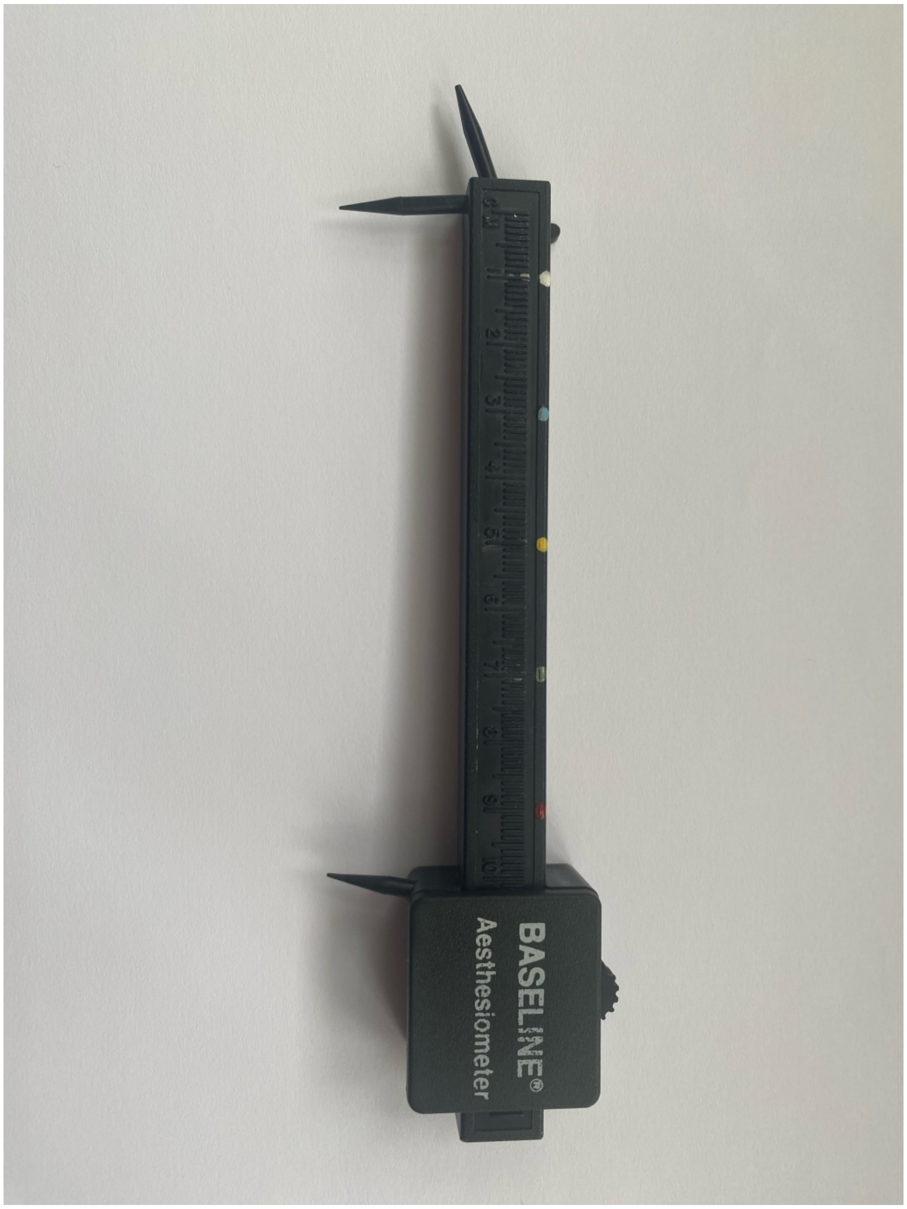

Figure 4. The marked two-point discrimination tool.

1066x1422mm (72 x 72 DPI)

For peer review only - http://bmjopen.bmj.com/site/about/guidelines.xhtml

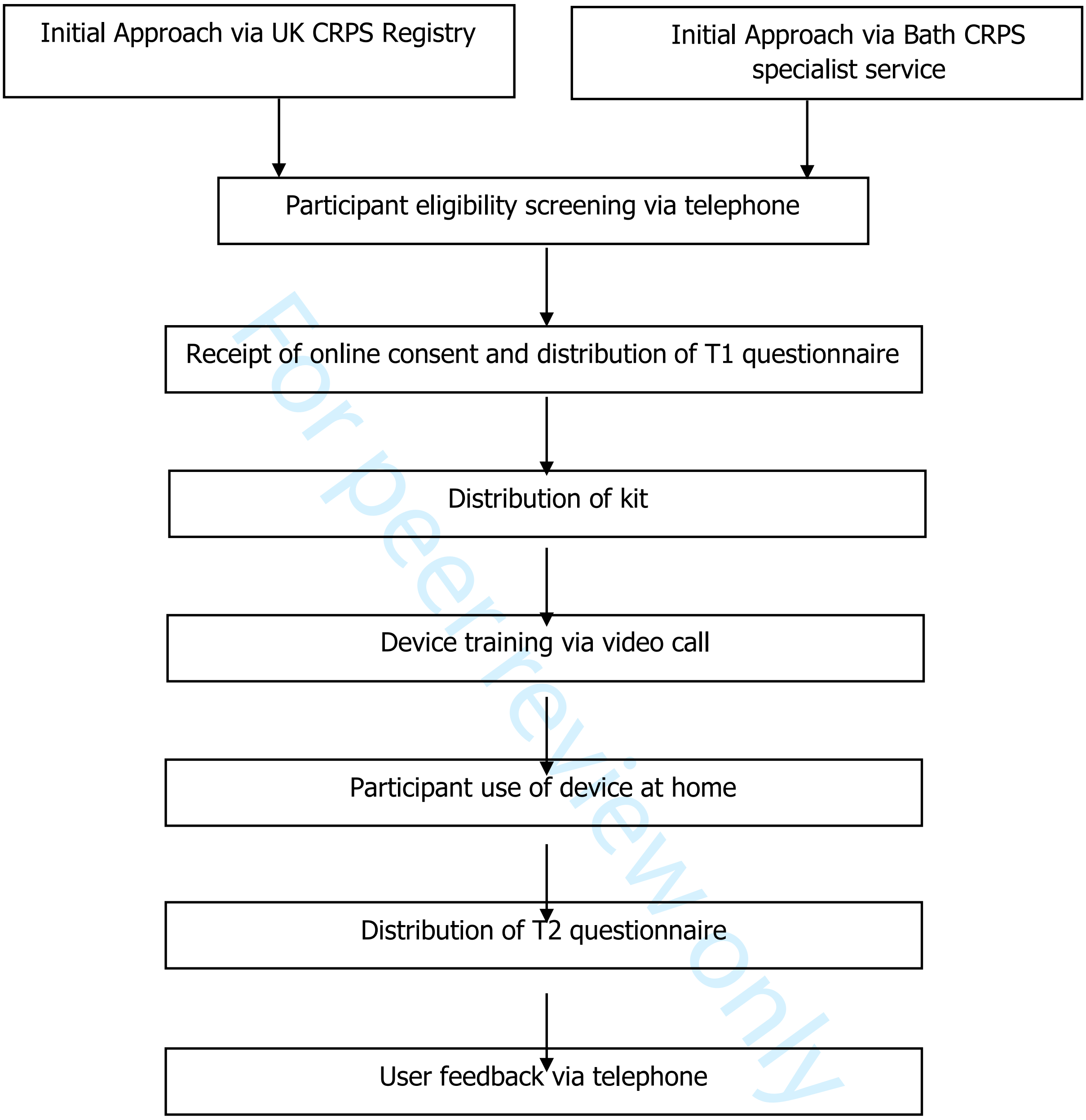

Figure 5. The STS study design.

SPIRIT

STANDARD PROTOCOL ITEMS: RECOMMENDATIONS FOR INTERVENTIONAL TRIALS

SPIRIT 2013 Checklist: Recommended items to address in a clinical trial protocol and related documents*

Section/Item	Item No	Description	Included? Page No
**Administrative information**			
Title	1	Descriptive title identifying the study design, population, interventions, and, if applicable, trial acronym	Yes - page 1
Trial registration	2a	Trial identifier and registry name. If not yet registered, name of intended registry	Yes - page 2
	2b	All items from the World Health Organization Trial Registration Data Set	Yes – throughout
Protocol version	3	Date and version identifier	Yes – page 6
Funding	4	Sources and types of financial, material, and other support	Yes – page 15
Roles and responsibilities	5a	Names, affiliations, and roles of protocol contributors	Yes – page 1
	5b	Name and contact information for the trial sponsor	Yes – page 16
	5c	Role of study sponsor and funders, if any, in study design; collection, management, analysis, and interpretation of data; writing of the report; and the decision to submit the report for publication, including whether they will have ultimate authority over any of these activities	Yes – page 16
	5d	Composition, roles, and responsibilities of the coordinating centre, steering committee, endpoint adjudication committee, data management team, and other individuals or groups overseeing the trial, if applicable (see Item 21a for data monitoring committee)	Yes – page 14
Introduction			
Background and rationale	6a	Description of research question and justification for undertaking the trial, including summary of relevant studies (published and unpublished) examining benefits and harms for each intervention	Yes – page 4

For peer review only

	6b	Explanation for choice of comparators – interviews	
Objectives	7	Specific objectives or hypotheses	Yes – page 6
Trial design	8	Description of trial design including type of trial (eg, parallel group, crossover, factorial, single group), allocation ratio, and framework (eg, superiority, equivalence, noninferiority, exploratory)	Yes – page 6
Methods: Participants, interventions, and outcomes			
Study setting	9	Description of study settings (eg, community clinic, academic hospital) and list of countries where data will be collected. Reference to where list of study sites can be obtained	Yes – page 6
Eligibility criteria	10	Inclusion and exclusion criteria for participants. If applicable, eligibility criteria for study centres and individuals who will perform the interventions (eg, surgeons, psychotherapists)	Yes – page 8 (table 1)
Interventions	11a	Interventions for each group with sufficient detail to allow replication, including how and when they will be administered	Yes – page 9 (one group only)
	11b	Criteria for discontinuing or modifying allocated interventions for a given trial participant (eg, drug dose change in response to harms, participant request, or improving/worsening disease)	Yes – page 9
	11c	Strategies to improve adherence to intervention protocols, and any procedures for monitoring adherence (eg, drug tablet return, laboratory tests)	N/A for this study
	11d	Relevant concomitant care and interventions that are permitted or prohibited during the trial	Yes – page 8 (exclusion criteria)
Outcomes	12	Primary, secondary, and other outcomes, including the specific measurement variable (eg, systolic blood pressure), analysis metric (eg, change from baseline, final value, time to event), method of aggregation (eg, median, proportion), and time point for each outcome. Explanation of the clinical relevance of chosen efficacy and harm outcomes is strongly recommended	Yes – page 11
Participant timeline	13	Time schedule of enrolment, interventions (including any run-ins and	Yes – pages 11-13

For peer review only - http://bmjopen.bmj.com/site/about/guidelines.xhtml

		washouts), assessments, and visits for participants. A schematic diagram is highly recommended (see Figure)	
Sample size	14	Estimated number of participants needed to achieve study objectives and how it was determined, including clinical and statistical assumptions supporting any sample size calculations	Yes – page 6
Recruitment	15	Strategies for achieving adequate participant enrolment to reach target sample size	Yes – page 7
**Methods: Assignment of interventions (for controlled trials)**			
Allocation:			
Sequence generation	16a	Method of generating the allocation sequence (eg, computer-generated random numbers), and list of any factors for stratification. To reduce predictability of a random sequence, details of any planned restriction (eg, blocking) should be provided in a separate document that is unavailable to those who enrol participants or assign interventions	N/A
Allocation concealment mechanism	16b	Mechanism of implementing the allocation sequence (eg, central telephone; sequentially numbered, opaque, sealed envelopes), describing any steps to conceal the sequence until interventions are assigned	N/A
Implementation	16c	Who will generate the allocation sequence, who will enrol participants, and who will assign participants to interventions	N/A
Blinding (masking)	17a	Who will be blinded after assignment to interventions (eg, trial participants, care providers, outcome assessors, data analysts), and how	N/A
	17b	If blinded, circumstances under which unblinding is permissible, and procedure for revealing a participant's allocated intervention during the trial	N/A
**Methods: Data collection, management, and analysis**			
Data collection methods	18a	Plans for assessment and collection of outcome, baseline, and other trial data, including any related processes to promote data quality (eg, duplicate measurements, training of assessors) and a description of study instruments	Yes – pages 11-12

For peer review only - http://bmjopen.bmj.com/site/about/guidelines.xhtml

		(eg, questionnaires, laboratory tests) along with their reliability and validity, if known. Reference to where data collection forms can be found, if not in the protocol	
	18b	Plans to promote participant retention and complete follow-up, including list of any outcome data to be collected for participants who discontinue or deviate from intervention protocols	Yes – page 10 (weekly contact)
Data management	19	Plans for data entry, coding, security, and storage, including any related processes to promote data quality (eg, double data entry; range checks for data values). Reference to where details of data management procedures can be found, if not in the protocol	Yes – page 12 and 14
Statistical methods	20a	Statistical methods for analysing primary and secondary outcomes. Reference to where other details of the statistical analysis plan can be found, if not in the protocol	N/A
	20b	Methods for any additional analyses (eg, subgroup and adjusted analyses)	N/A
	20c	Definition of analysis population relating to protocol non-adherence (eg, as randomised analysis), and any statistical methods to handle missing data (eg, multiple imputation)	N/A
**Methods: Monitoring**			
Data monitoring	21a	Composition of data monitoring committee (DMC); summary of its role and reporting structure; statement of whether it is independent from the sponsor and competing interests; and reference to where further details about its charter can be found, if not in the protocol. Alternatively, an explanation of why a DMC is not needed	N/A
	21b	Description of any interim analyses and stopping guidelines, including who will have access to these interim results and make the final decision to terminate the trial	N/A
Harms	22	Plans for collecting, assessing, reporting, and managing solicited and spontaneously reported adverse events	Yes – page 14

		and other unintended effects of trial interventions or trial conduct	
Auditing	23	Frequency and procedures for auditing trial conduct, if any, and whether the process will be independent from investigators and the sponsor	Yes – page 14
Ethics and dissemination			
Research ethics approval	24	Plans for seeking research ethics committee/institutional review board (REC/IRB) approval	Yes – page 15
Protocol amendments	25	Plans for communicating important protocol modifications (eg, changes to eligibility criteria, outcomes, analyses) to relevant parties (eg, investigators, REC/IRBs, trial participants, trial registries, journals, regulators)	Yes – page 15
Consent or assent	26a	Who will obtain informed consent or assent from potential trial participants or authorised surrogates, and how (see Item 32)	Yes – page 9
	26b	Additional consent provisions for collection and use of participant data and biological specimens in ancillary studies, if applicable	N/A

*It is strongly recommended that this checklist be read in conjunction with the SPIRIT 2013 Explanation & Elaboration for important clarification on the items. Amendments to the protocol should be tracked and dated. The SPIRIT checklist is copyrighted by the SPIRIT Group under the Creative Commons “Attribution-NonCommercial-NoDerivs 3.0 Unported” license.

For peer review only
